# Systematic Review and Spatiotemporal Assessment of Mercury Concentration in Fish from the Tapajós River Basin: Implications for Environmental and Human Health

**DOI:** 10.1021/acsenvironau.4c00053

**Published:** 2024-10-30

**Authors:** Karen L. Auzier Guimarães, Sarah J. do Nascimento Andrade, Ahieska A. Liscano-Carreño, Ricardo B. de Oliveira, Luís R. Ribeiro Rodrigues

**Affiliations:** † Programa de Pós-Graduação em Biodiversidade e Biotecnologia (REDE BIONORTE), Instituto de Saúde Coletiva (ISCO), Universidade Federal do Oeste do Pará (UFOPA), Rua Vera Paz, s/no, Salé, CEP, 68040-255 Santarém, Pará, Brazil; ‡ Laboratório de Genética & Biodiversidade (LGBio), Instituto de Ciências da Educação (ICED), Universidade Federal do Oeste do Pará (UFOPA), Rua Vera Paz, s/no, Salé, CEP: 68040-255 Santarém, Pará, Brazil; § Departamento de Biología, Universidad de Oriente (UDO), Avenida Universidad, s/no, 6101 Cumaná, Sucre, Venezuela; ∥ Laboratório de Bioprospecção e Biologia Experimental, Instituto de Ciências da Educação (ICED), Universidade Federal do Oeste do Pará (UFOPA), Rua Vera Paz, s/no, Salé, CEP, 68040-255 Santarém, Pará, Brazil

**Keywords:** bioaccumulation, environmental pollution, gold mining, health risks, mercury contamination

## Abstract

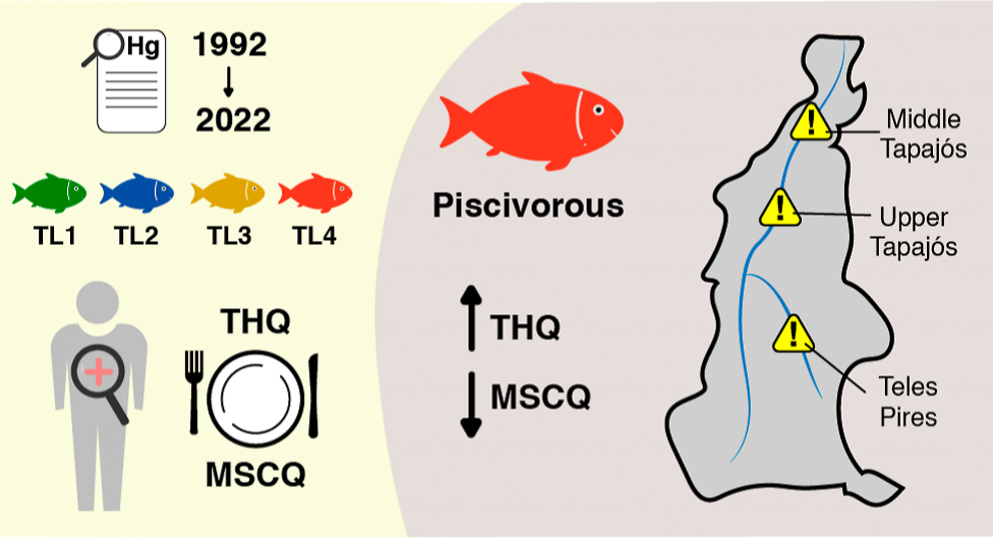

This study reviews the literature on mercury (Hg) pollution in the Tapajós River basin from 1992 to 2022, focusing on the bioaccumulation in fish and the associated health risks to humans via ingesting contaminated species. Variability in Hg bioaccumulation was analyzed from both spatial (sub-basins) and ecological (trophic levels) perspectives. Mercury concentrations in fish muscle tissue and spatial differences in Hg levels were analyzed using nonparametric Kruskal–Wallis ANOVA and mapped with Inverse Distance Weighting. Additionally, a risk assessment of mercury contamination was conducted using the Target Hazard Quotient (THQ) and Maximum Safe Consuming Quantity (MSCQ) indices. Results indicate that Hg contamination is pervasive across the basin, with piscivorous fish showing the highest Hg levels, particularly in the middle Tapajós, upper Tapajs óand Teles Pires sub-basins, identified as contamination hotspots. Piscivorous species exhibited high Target Hazard Quotients (THQ), suggesting health risks for local consumers. The MSCQ values indicated that 75% of the fish species analyzed should be consumed in quantities lower than the current consumption daily average to avoid health risks.

## 1 Introduction

Mercury (Hg) is a toxic heavy metal that, in aquatic environments, can be transformed into the organic methylmercury form through microbial activity. This transformation enables Hg to accumulate in organisms and undergo biomagnification along the food chain.^1,2^

In the Amazon basin, the Tapajós River has been affected by the disposal of mercury linked to artisanal gold mining for several decades.^3−7^ Furthermore, deforestation in the Amazon, extensive biomass burning, the expansion of agricultural areas, and the existence of dams contribute to the emission and mobilization of Hg in soils and water bodies,^8,9^ making the Tapajós River a hotspot for mercury pollution.^1^

Gold mining, known in the Amazon as “garimpo”, has aroused growing concern due to its environmental impacts and implications for human health linked to mercury contamination. While the scientific literature reports many studies on human exposure and bioaccumulation of mercury in fish, there is a scarcity or virtual absence of studies on assessing the effects of Hg pollution on Amazonian biodiversity. The mercury pollution in aquatic ecosystems represents a serious environmental and public health problem in many regions of the world.^10−12^ Despite the diversification of food sources due to agricultural expansion, pisciculture, and large wholesale networks, local fish remains an important part of the diet for many Amazonian populations. Consequently, these populations are inherently exposed to the risk of mercury contamination by consuming contaminated fish.^5,13−16^

The mining region of the middle and upper Tapajós is home to several Conservation Units (CU) and Indigenous Lands (IL), which are important for composing the southern ecological corridor of the Amazon, and therefore, have an immeasurable and strategic value for conservation and balance of the ecosystems of the Amazon biome.^17,18^ Gold exploration in the Tapajós basin advanced within the limits of these important protected areas, such as in the CU: APA Tapajós (516 km^2^), FLONA Amana (79 km^2^), FLONA Crepori (23 km^2^) and PARNA Rio Novo (23 km^2^); and in the IL: Kayapó (137 km^2^), Munduruku (55 km^2^) and Sai-Cinza (3.7 km^2^).^19^ Protected areas are considered essential for biodiversity conservation.^18^ The degradation of these areas by gold mining in the Tapajós basin implies the generation of biodiversity loss processes in a part of the Amazon recognized as a center of endemism—Tapajós Endemism Area.^20^ Furthermore, the Tapajós basin is considered one of the areas of endemism in the Amazon that remains poorly studied.^21^

The finding of mercury levels above permissible limits [e.g., Agência Nacional de Vigilância Sanitária (ANVISA) or World Health Organization (WHO) limits] in various fish species in the Tapajós River basin is firmly established in extensive scientific literature.^22^ This has recently raised serious concerns regarding the food safety and health of riverside communities and traditional peoples.^4,14,23,24^ However, it is essential to consider not only the mercury levels in fish but also the quantities of fish consumed. High fish consumption can lead to high mercury intake, making the amount of fish consumed an important factor in assessing health risks.^25^ Most studies on mercury pollution in the Tapajós River have focused on commercially important fish species and covering a short period.^26,27^ The last comprehensive review specifically addressing mercury in fish in this basin was published over ten years ago and includes only data extracted from 11 articles published between 1995 and 2008 (see ref (22)).

In the present study, a comprehensive systematic review was conducted to establish a database for assessing the distribution and variation of mercury contamination in different fish species and trophic levels along the Tapajós River basin. Additionally, the data were used to evaluate the risk of exposure to mercury contamination through the consumption of contaminated fish.

## 2 Material and Methods

### 2.1 Study Area

**Figure 1:**
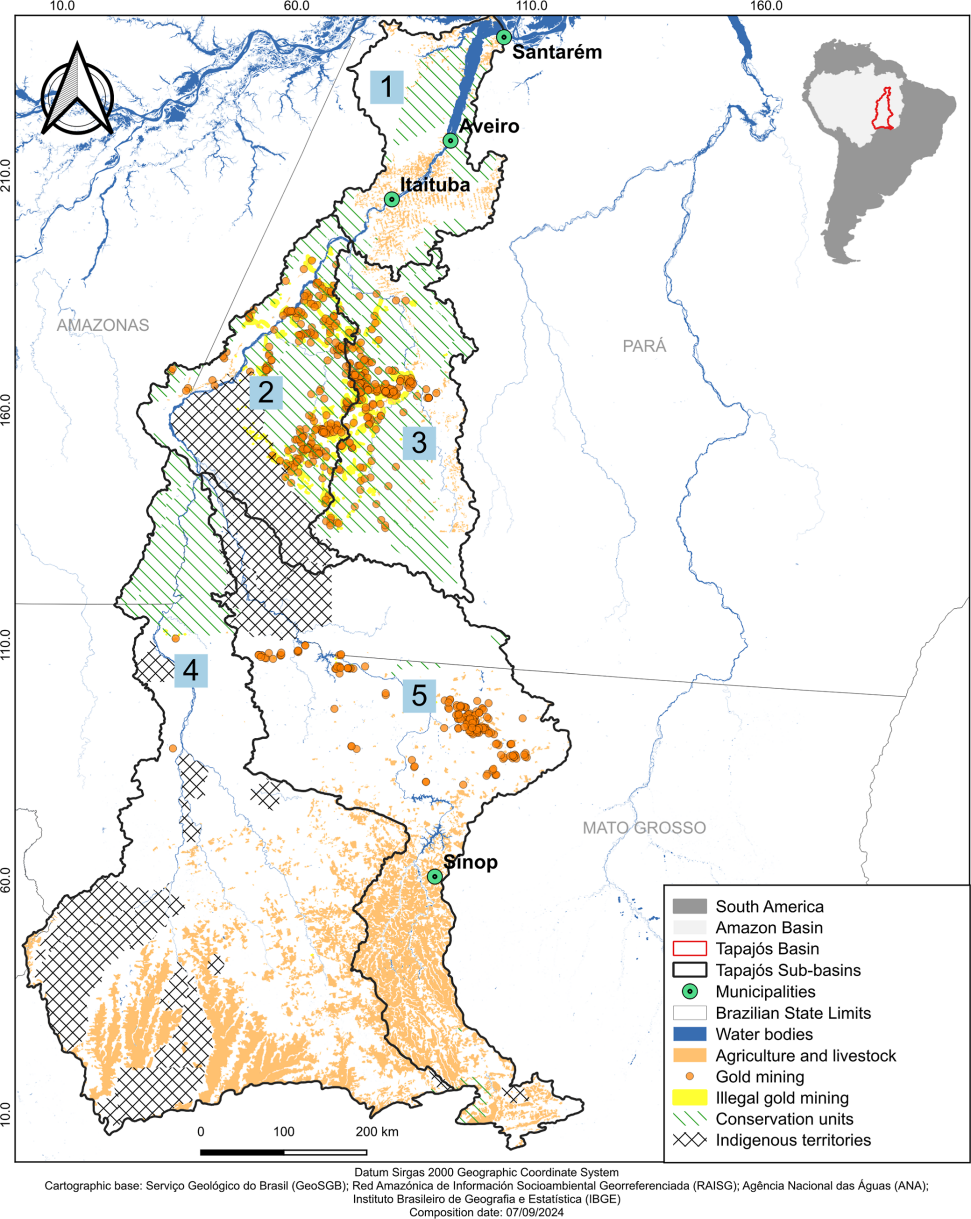
Patterns and elements of the landscape in the Tapajós River basin, with a focus on the distribution of geographical and environmental aspects, including illegal gold mining sites, gold mining points, indigenous territories, CU, and areas of agriculture and livestock. Sub-basins: (1) Lower Tapajós, (2) Upper Tapajós, (3) Jamanxim, (4) Juruena, and (5) Teles Pires. The map was done with QGIS (v.3.30).

The Tapajós River is the fifth largest tributary of the Amazon River, with a drainage area covering 492,263 km^2^, spanning territories in the states of Mato Grosso, Pará, Rondônia and Amazonas, extending between the Central-West and Northern regions of Brazil. Its main tributaries include the Teles Pires, Juruena, Jamanxim and Crepori rivers.^28,29^ The area is characterized by a diversity of landscapes, including indigenous territories, CU, urban areas, gold mining, and agricultural expansion. The Tapajós River basin is divided, according to the classification of the Agência Nacional das Águas (ANA), into five sub-basins: (1) Lower Tapajós, (2) Upper Tapajós, (3) Jamanxim, (4) Juruena, and (5) Teles Pires (Figure 1). The most populous city in the basin is Santarém (PA), located in the Lower Tapajós, with 308,339 inhabitants, followed by Sinop (MT) on the Teles Pires River with 148,960 inhabitants, and Itaituba (PA) with 101,541 inhabitants, covering the Middle and Upper Tapajós and part of the Jamanxim River sub-basin.^30^

### 2.2 Systematic Literature Review

Studies focusing on aquatic mercury pollution in the Tapajós River basin were obtained through a systematic search following the PRISMA guideline^31^ (Figure S1). The bibliographic material was collected until February 22, 2023, using the following databases: Web of Science, PubMed, SCOPUS and the “Observatório do mercúrio”, which is a georeferenced data platform gathering studies on mercury contamination in the Pan-Amazon from 1980 to 2021.^32^ The following keywords were used: “Tapajós”, “mercury” and “fish”, combined using Boolean operators. Additionally, reference lists of articles were reviewed and original data provided by the Laboratório de Bioprospecção e Biologia Experimental at Universidade Federal do Oeste do Pará (UFOPA) were included.

### 2.3 Database

To create the database, we adopted the following inclusion criteria: (1) the field research should have sampled the Tapajós River basin area, (2) the data should include geographical coordinates of the sampling points or the municipality-level location where the fish were collected, with the possibility of approximation if coordinates were not available, (3) the statistical data should include values of THg (total mercury) or MeHg (methylmercury) for muscle tissue per species or, if not available, consider the trophic level (TL), (4) the sampling year was taken as a reference, or, in the absence of this information, the manuscript receipt date or the publication date were considered. The muscle tissue was chosen because almost all the documents analyzed report on this material. After screening and eligibility of bibliographic sources, the following data were extracted: collection site, geographical coordinates, sampling year, species, trophic level, sample size, mean mercury concentration, and first author’s name (Table S2). The taxonomic nomenclature of the species was reviewed, validated, or updated according to Eschmeyer’s Catalog of Fishes.^33^

### 2.4 Trophic Level Classification

**Figure 2:**
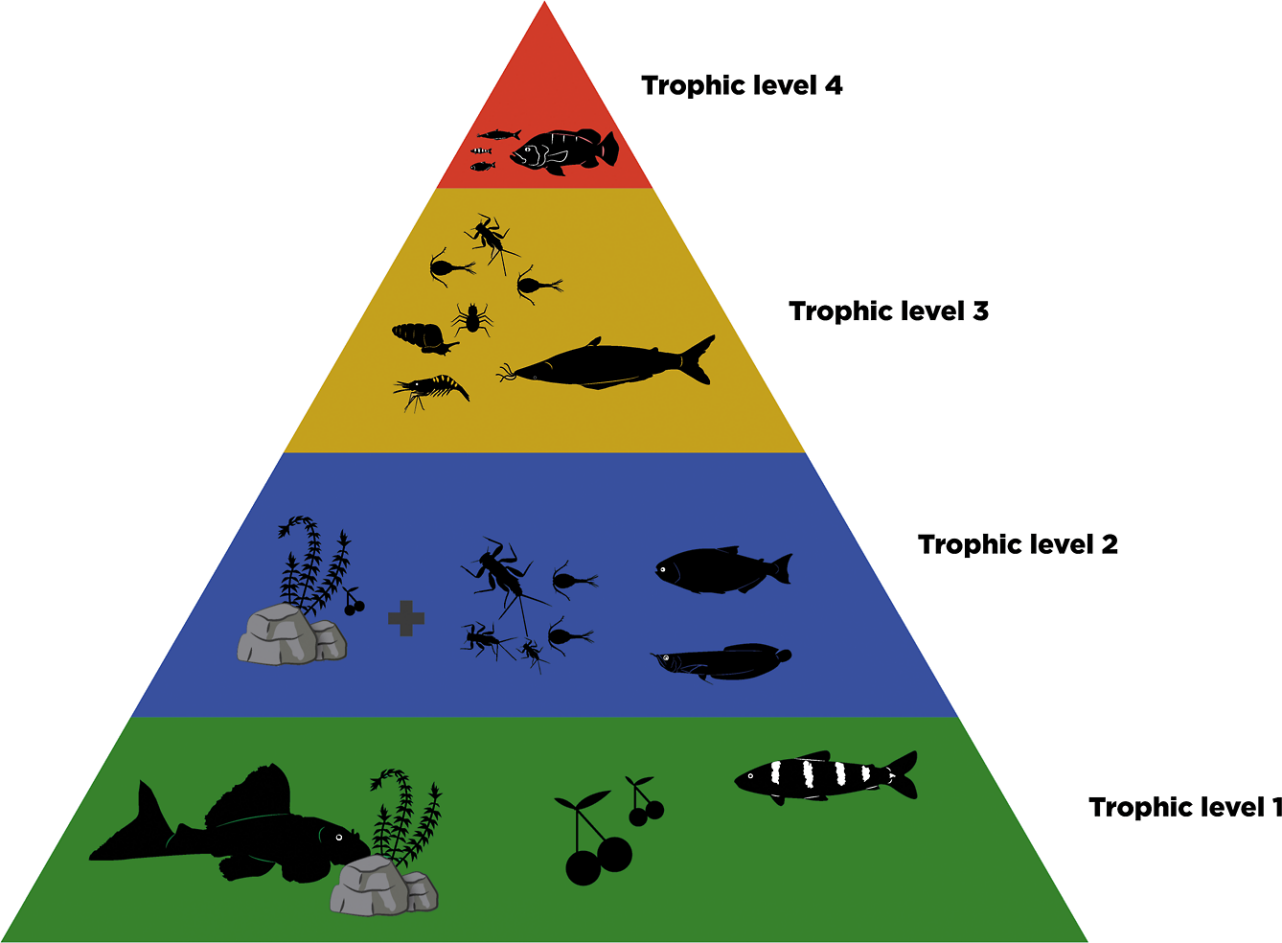
Classification of fish species according to the four trophic levels: TL1: includes herbivorous and detritivorous species, TL2: omnivorous species, TL3: invertivorous and planktivorous species, and TL4: piscivorous species.

The species were classified according to their feeding strategies based on relevant literature.^34−45^ The evaluated species were classified into six different trophic categories: detritivorous, herbivorous, invertivorous, omnivorous, piscivorous, and planktivorous. We used Röpke et al. (2017)^39^ to define feeding habits within each category but applied our own numerical classification for the trophic levels, using a scale of 1 to 4: Trophic level 1 (TL1)—herbivorous and detritivorous species that primarily ingest plant material (seeds, fruits, or leaves), filamentous algae, and fine particulate organic matter originating mainly from periphyton; Trophic level 2 (TL2)—omnivorous species that ingest combinations of animals, plants, and detritus; Trophic level 3 (TL3)—invertivorous and planktivorous species that predominantly feed on insects (adults or immature forms of aquatic and terrestrial insects), benthic or water column microcrustaceans, spiders, shrimp, and/or mollusks; Trophic level 4 (TL4)—piscivorous species that ingest adult, juvenile, or larval fish, whole or in pieces, such as scales and fins (Figure 2). This adjustment from the original scale of 1, 1.5, 2, and 3^39^ was made to simplify the classification process and improve interpretability given the context of our study.

### 2.5 Spatial Variation

The total mercury (THg) values found in fish were converted to mg/kg and compared to the reference values established by the Agência Nacional de Vigilância Sanitária of Brazil (ANVISA) for piscivorous and nonpiscivorous fish^46^ and the WHO. The mean Hg concentration values per species and per locality were converted into classes and mapped using the Inverse Distance Weighting interpolation method, performed in Quantum GIS software (version 3.30). In this study, we use the term “locality” to refer to specific sampling points where data were collected. Each locality represents a distinct georeferenced point within the study area. For this mapping, all mean values found at each georeferenced point in each of the four investigated trophic levels were considered. This analysis was conducted considering the Tapajós river basin as a whole. However, to enhance the visualization and interpretation of the results, the data were mapped by separating them into sub-basins.

The normality of the data was assessed using the Shapiro–Wilk test and did not show normality (*p* > 0.05). Thus, spatial differences in mercury concentrations were analyzed using the nonparametric Kruskal–Wallis ANOVA and the posthoc Bonferroni test at a significance level of *p* < 0.05. Statistical analyses and visualization graphics were performed using Statgraphics v.19 software (https://www.statgraphics.com/download19) and Microsoft Office Excel 2019.

To categorize and compare sub-basins in the Tapajós River basin, we adopted the classification of areas according to the Agência Nacional das Águas (ANA) (Figure 1). However, in our study, the “lower Tapajós”, as defined by ANA, were further subdivided into “lower Tapajós” and “middle Tapajós”. We delimited the lower Tapajós River as the widest portion of the river from the municipality of Aveiro to its confluence with the Amazon River in the municipality of Santarém, while the “middle Tapajós” encompasses the area from the upstream limit defined by ANA to the municipality of Aveiro (Figure S3). The Juruena River sub-basin was not included in the analysis because there was only one available data point in the literature for this area.^47^

To analyze the spatial variation of mercury (Hg) bioaccumulation in the piscivorous fish group, we applied Moran’s I index as a measure of spatial autocorrelation. This analysis was conducted to identify the presence of spatial patterns in the distribution of Hg concentrations, using only locations with species having *n* ≥ 10.

### 2.6 Temporal Variation

The variations in total mercury (THg) concentrations over time and across different trophic levels were assessed based on all articles published from 1992 to 2022. These articles were divided into three decades (1992–2001, 2002–2011, 2012–2022). The analysis also considered changes in the number of studies over time and examined potential associations between temporal factors and the number of articles published on mercury concentrations. Statistical analyses included Kruskal–Wallis tests for nonparametric data, Bonferroni posthoc tests at a significance level of *p* < 0.05, and simple linear regression to determine the most appropriate model fit (double square). Statistical analyses were performed using Statgraphics v.19 software (https://www.statgraphics.com/download19).

### 2.7 Risk Assessment of Mercury Contamination

#### 2.7.1 Estimation of Food Consumption

Following the Household Budget Survey of 2017–2018 conducted by the Instituto Brasileiro de Geografia e Estatística (IBGE), the consumption of freshwater fish in Brazil in 2018 was reported as 0.903 kg/person/year. In the North region of Brazil, it was 5.450 kg/person/year, and in the Midwest, it was 0.705 kg/person/year.^48^ However, these values may not reflect the reality of local populations in the Tapajós River basin, as considerable differences are observed in different available studies.^25,49−51^ The total fish consumption per adult in the Tapajós River basin can vary from 8 g/person/day^50^ to 217 g/person/day,^25^ depending on the locality. Therefore, for the calculation of the Target Hazard Quotient, the per capita fish consumption (g) (IRd) considered was the average value from available studies in the Tapajós River basin, which was 116.25 g per capita per day. An average of 70 kg body weight (BW) is assumed for an adult person, and a year consists of 365 days.

#### 2.7.2 Estimation of Noncarcinogenic Risk

The THQ (Target Hazard Quotient) is a method for assessing the potential health risk posed by any chemical contaminant over a lifetime of exposure.^52^ The value of this index depends not only on the level of the contamination of the food, but also on the rate of food intake, the frequency and duration of exposure, the average BW and the reference oral dose. A THQ value ≥1 indicates a potential health risk to the human body.^52−54^ We used THQ to evaluate the noncarcinogenic risk for the population of the Tapajós River basin, through the following equation
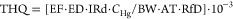
where EF is the exposure frequency (365 days per year); ED is the exposure duration equivalent to the average age of the Brazilian adult (77 years); IRd is the per capita fish consumption (g); *C*_Hg_, is the mercury concentration (mg/kg); BW, is the average human BW; AT (=EF. ED), is the exposure time for noncarcinogens, reference dose (RfD), is the oral RfD (mg/kg/day). The oral RfD for MeHg used was 0.0001 (mg/kg/day).^55^

#### 2.7.3 Maximum Safe Consuming Quantity

The Maximum Safe Consuming Quantity (MSCQ) index allows for determining the maximum allowable rates of potentially contaminated fish (food) that could be safely ingested daily.^52^ For adults, the MSCQ index was estimated using the equation below
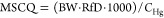
where BW is the human BW (in kg), RfD is the oral RfD, and *C*_Hg_ is the mercury concentration expressed in (mg/kg). When the MSCQ is greater than the average daily fish intake (in grams), the food does not pose a health risk. The daily fish consumption considered was 116.25 g per capita for an adult person in the Tapajós River basin.

## 3 Results

### 3.1 Literature Review

**Figure 3:**
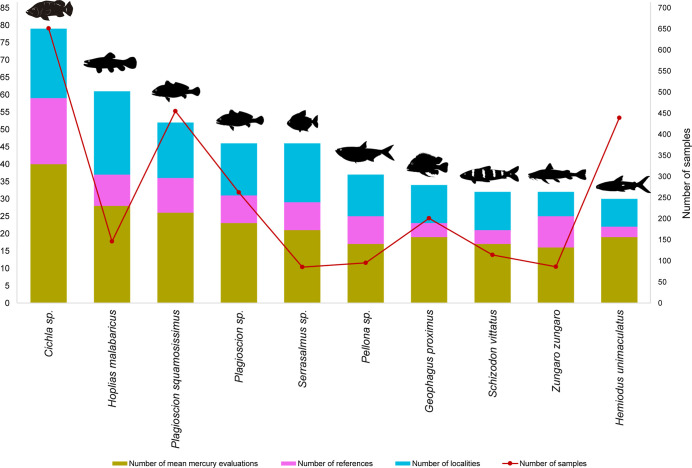
Total of average mercury (Hg) values for the most evaluated fish species.

**Figure 4:**
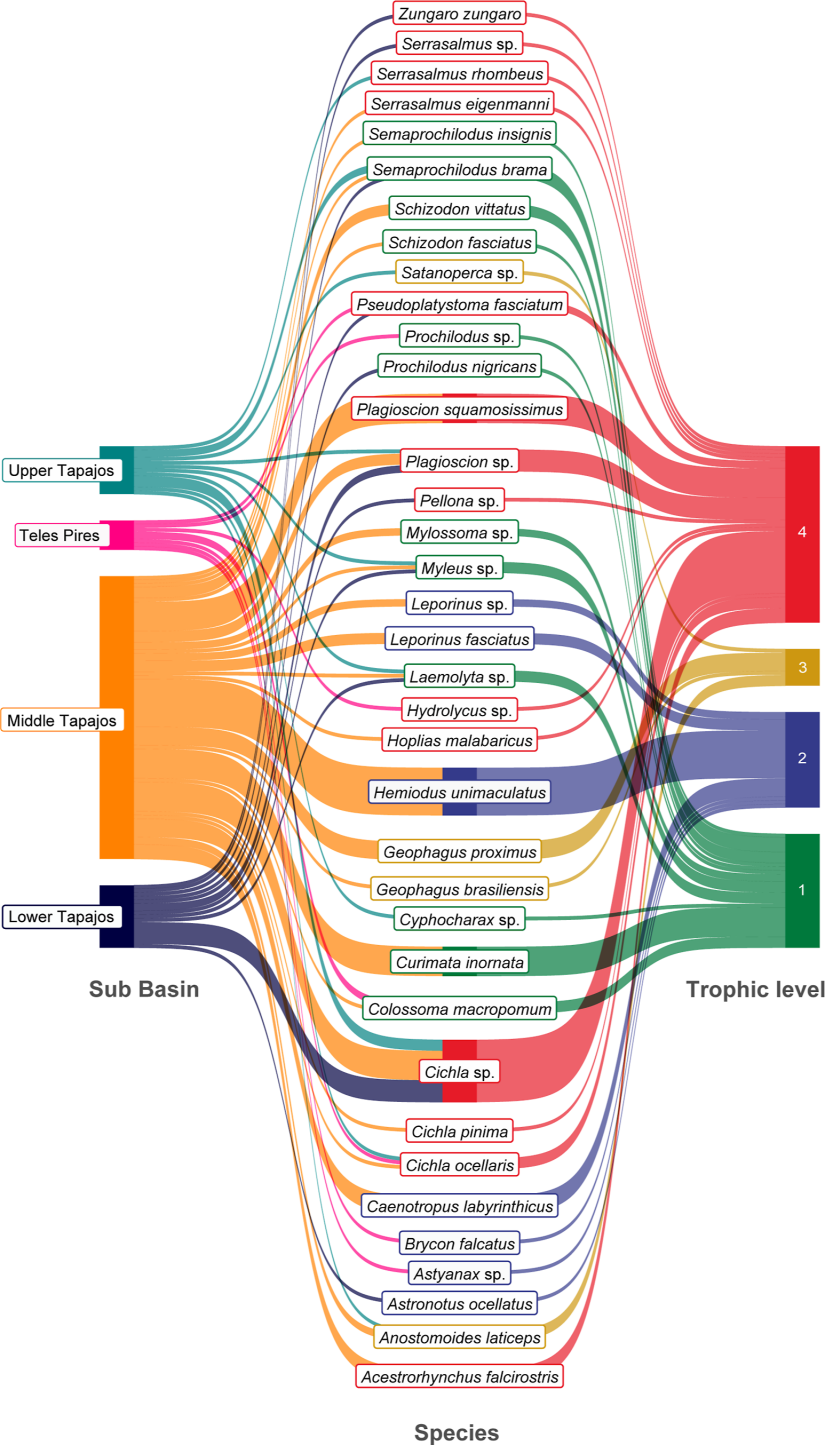
Sankey diagram illustrating the distribution of fish species with a sample size of at least 15 individuals per study across different sub-basins of the Tapajós River and their respective trophic levels.

The bibliographic search, after filtering and duplicate removal, resulted in 36 documents published between 1992 to 2022. The data set is representative of 735 average Hg values from 143 species and 14,113 individuals evaluated. *Cichla* sp. (tucunaré) is the most reported taxon, *n* = 651 samples (39 average Hg values), cited in 19 papers (Figure 3, Table S4). Although the majority of species were subsampled (*n* < 10 individuals for paper), we observed 37 species that were investigated with a minimum of 15 individuals per sample, the majority focus is on TL4 species in the middle Tapajós (Figure 4, Tables S2, S4). A total of 19 species had a single individual analyzed (Table S4).

The Hg concentration was assessed in fishes from 59 sampling sites (ss) along the Tapajós river basin. The middle Tapajós was the most studied (*n* = 111 sp., 7479 indiv., ss = 27), followed by upper Tapajós (*n* = 48 sp., 2026 indiv., ss = 13), lower Tapajós (*n* = 37 sp., 2507 indiv., ss = 6), Teles Pires (*n* = 34 sp., 909 indiv., ss = 11), Jamanxim (*n* = 11 sp., 30 indiv., ss = 1) and Juruena (70 indiv., ss = 1) (Tables S2–S4). *Hoplias malabaricus* was the most widely studied taxon along the basin area (ss = 24).

An exploratory analysis of the literature showed a clear discrepancy in the study density about the sub-basins of the Tapajós river basin. The middle Tapajós has received the most attention, with 21 publications. In contrast, the lower Tapajós have 12 publications, while both the upper Tapajós and Teles Pires have only 10 each. Research in Jamanxim and Juruena is particularly limited, with just one publication in each (Table S4).

### 3.2 Hg Bioaccumulation and Trophic Categories

**Figure 5:**
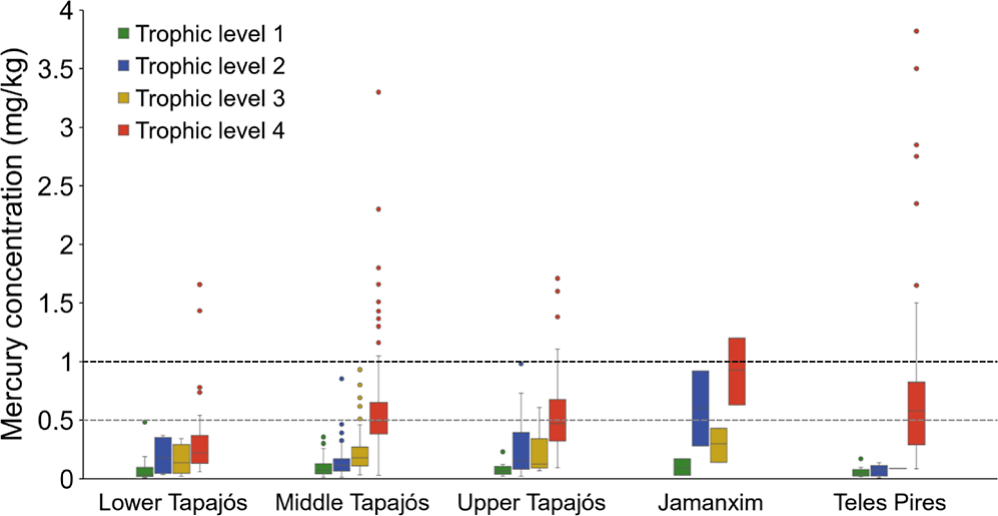
Dispersion of Hg concentration measurements (mg/kg) in fish from Tapajós river basin.

Fish taxa were sorted according to their ecological diet attributes into four trophic levels: TL1—herbivorous and detritivorous (42 sp., 29, 17%), TL2—omnivorous (26 sp., 18,18%), TL3—invertivorous and planktivorous (21 sp., 14,58%) and TL4—piscivorous (54 sp., 37,5%). The Hg concentration observed across all samples analyzed ranged from 0.01 to 3.82 mg/kg (Figure 5). The lowest averages (0.01) were measured in species of TL1 (*Prochilodus* sp., *Schizodon vittatus*, *Myleus* sp. e *Piaractus brachypomus*) and TL2 (*Pachypops* sp. and *Brycon* sp.), while the largest averages (3.82) in the TL4 (*Brachyplatystoma filamentosum*) (Figure S5). Hg concentration overall range observed across all samples analyzed varied significantly between trophic levels (KW = 379.061, *p* < 0.05), with exception between the levels 1–2 and 2–3.

Based on the average mercury levels measured across all samples, a total of 16 piscivorous species (29%) had mercury concentrations above the limit established by ANVISA. ANVISA’s limit for piscivorous fish is 1 mg/kg, whereas for nonpiscivorous fish it is 0.5 mg/kg. In contrast, the WHO standard applies a limit of 0.5 mg/kg for all fish species. Considering the WHO regulation, the number of piscivorous species exceeding the limit rises to 36 (67%). The nonpiscivorous species *Hemiodus unimaculatus*, *Cetopsis candiru*, *Pimelodus blochii* of TL2 and *Auchenipterus* sp., *Osteoglossum bicirrhosum*, *Geophagus* sp. and *Hypophthalmus* sp. of TL3 had a mercury concentration above that allowed by ANVISA and WHO (>0.5 mg/kg) (Figure S5). All the TL1 species had Hg concentration below 0.5 mg/kg. However, *Mylossoma duriventre* and *Schizodon fasciatus* from the lower Tapajós had values close to the limit (0.48 and 0.49 mg/kg, respectively).

### 3.3 Spatial Data Analysis

**Figure 6:**
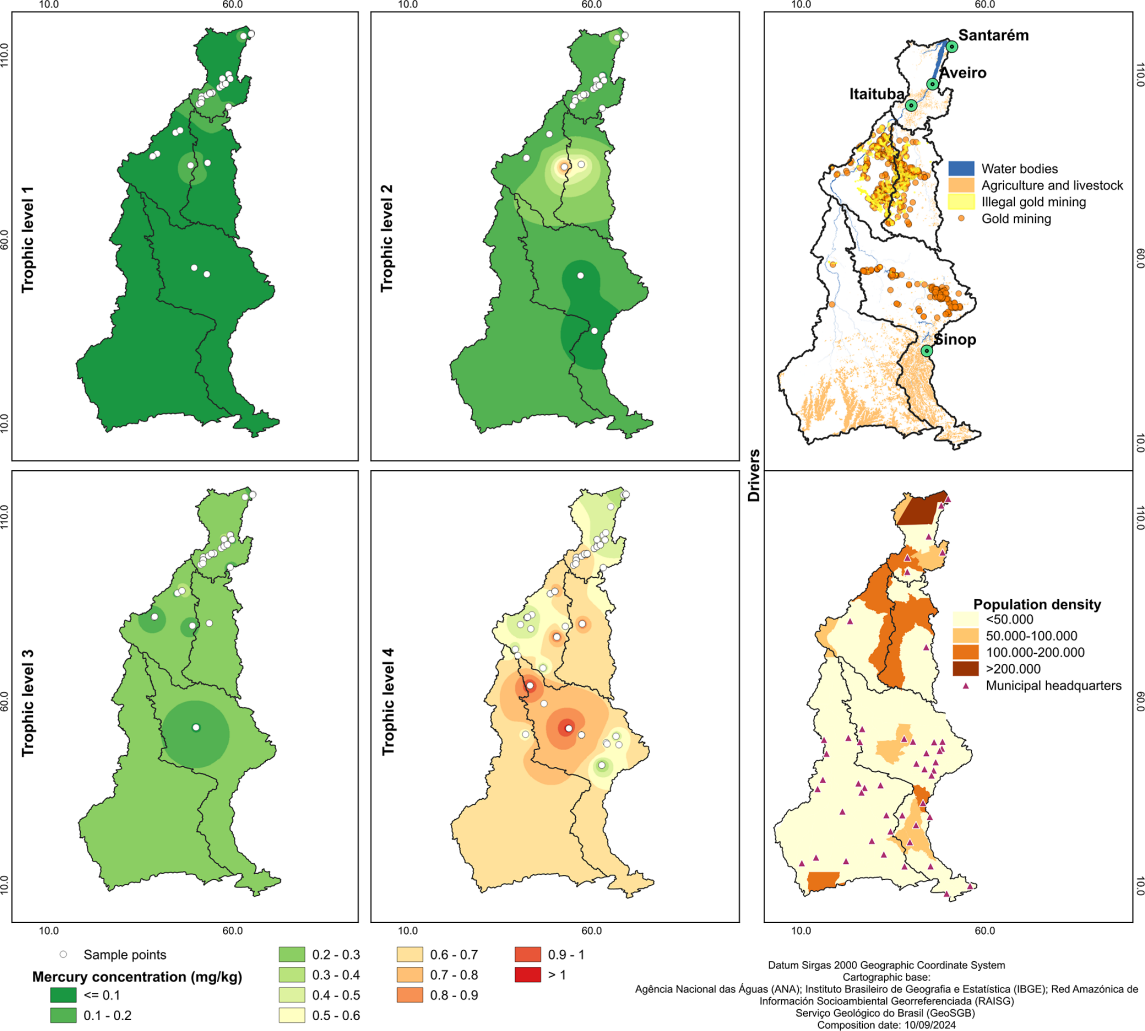
Spatial pattern of Hg bioaccumulation (mg/kg) in fish from Tapajós river basin in relation to trophic levels. Color gradients were obtained from interpolation processing. Gold mining (garimpos), land use and human population density are shown in driver boxes. Maps were done with QGIS (v.3.30).

Georeferenced average Hg values of fish species plotted on a map and processed by interpolation method revealed patterns of Hg contamination along the Tapajós river basin and through the distinct trophic levels. While herbivorous and detritivorous (TL1) did not show variation across the entire basin, the omnivorous (TL2) had a punctual increase of Hg bioaccumulation in the border of upper Tapajós and Jamanxim sub-basins. Invertivorous and planktivorous fish (TL3) showed a discrete Hg bioaccumulation increase compared to level 1 and level 2 fishes, however as similar to level 1 it did not exhibit variation along the basin. The piscivorous (TL4) showed a markedly higher Hg bioaccumulation along the Tapajós basin, that were statistically distinct between the sub-basins (KW = 42.687, *p* < 0.001) where Teles Pires sub-basin presented the higher values of Hg in piscivorous, while Lower Tapajós sub-basin had the lowest ones (Figure 6). Once Hg bioaccumulation in the piscivorous group exhibited a clear spatial variation we tested it for spatial autocorrelation with Moran *I* index. We found a moderate correlation (*I* = 0.360) that indicates a clustering of points with high average Hg values in fish from the middle Tapajós (Figure S6).

The statistical analysis in Hg concentration did not vary between the sub-basins for the TL1 (KW = 9.495, *p* > 0.05), TL2 (KW = 7.988, *p* > 0.05) and TL3 (KW = 3.198, *p* > 0.05) along the Tapajós river basin, in contrast the bioaccumulation in piscivorous fish (TL4) varied significantly along the study area (KW = 42.687, *P* < 0.001). For TL2, while the Kruskal–Wallis test did not show significant variation, the Bonferroni test revealed significant differences in Hg bioaccumulation specifically in the Jamanxim sub-basin compared to Lower Tapajós, Middle Tapajós and Teles Pires (Table S7).

### 3.4 Temporal Variation

For the temporal variation analyses, the first step was to evaluate THg concentrations by decade. No statistically significant differences were found between the decades (KW: 0.13, *p* > 0.05). Although the past decade shows greater data dispersion, it has the smallest mercury number measurements (*n* = 105). In comparison, the first decade includes 384 measurements, and the second decade has 246 measurem ents of Hg, highlighting a clear disparity in the number of published articles between the first period (*n* = 18) and the following two decades, with 8 articles in the second decade and 10 in the most recent decade (KW: 457.81, *p* < 0.001). Moreover, relatively weak associations were found between the decades and the number of articles published on Hg (*r*^2^: 0.09, *p* < 0.05, β: 0.03; *r*^2^: 0.22, β: −0.78), indicating minimal influence on this variable (Table S8).

Regarding variations in different trophic levels (TL) over time, only TL1 showed statistical differences between the first and second decades (KW: 15.13, *p* < 0.001). No statistical differences were found for TL2 and TL3 (KW: 4.14, *p* > 0.05; KW: 1.22, *p* > 0.05, respectively). Although differences were observed for TL4, but the Bonferroni post hoc test did not reveal significant variations (KW: 10.18, *p* < 0.05) (Table S9).

### 3.5 Risk Assessment for Human Health

**Figure 7:**
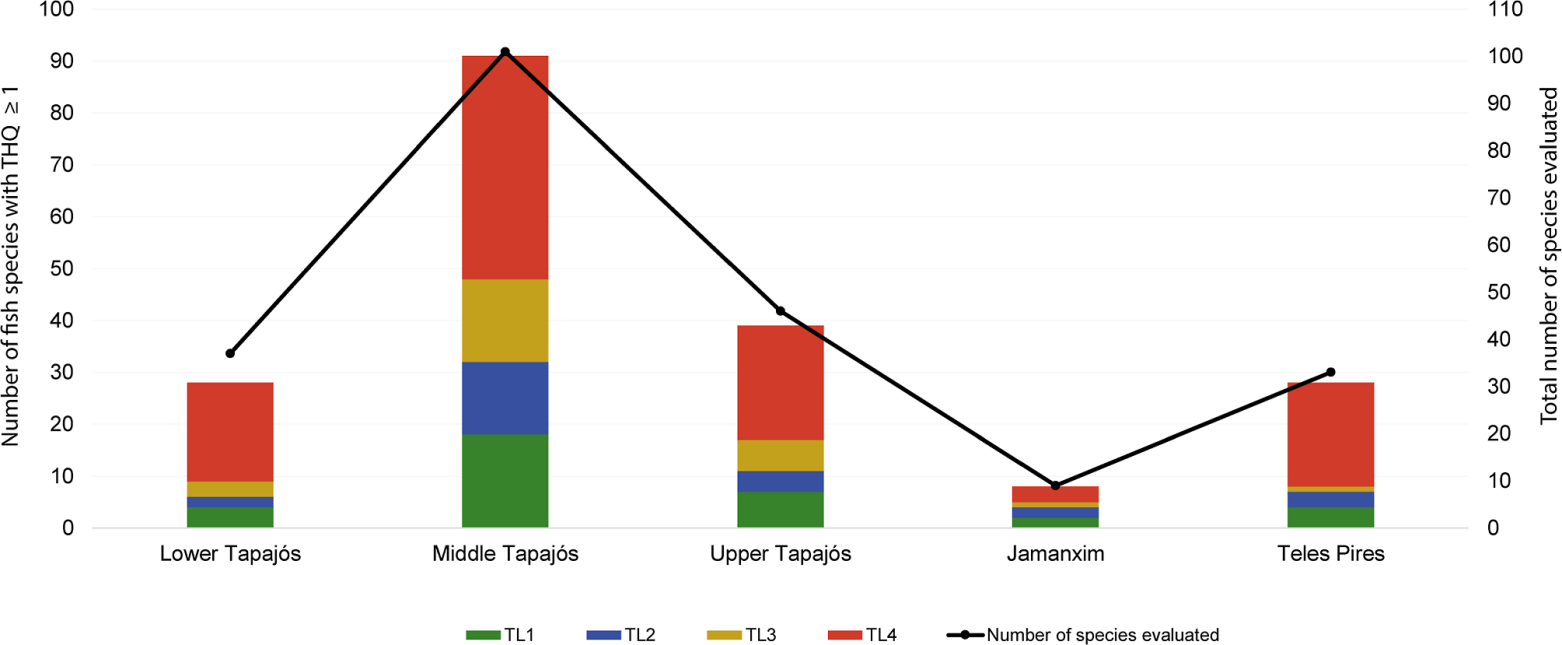
Assessment of fish species for human health risk based on Hazard Quotient (THQ) for Hg exposure by ingestion of contaminated fish from Tapajós river basin.

We evaluated 129 fish species reported as important for human consumption. Based on the Target Hazard Quotient (THQ), 115 species (89%) in at least one of the evaluated sub-basins showed potential risk to human health. Only 14 species did not present THQ values ≥1, and these species were distributed across trophic levels 1 and 2. The middle Tapajós sub-basin is the most dangerous to human health since 90% (*n* = 91) of fish species have THQ ≥1. In both the adjacent areas, lower (*n* = 28) and upper Tapajós (*n* = 39), the human risk for mercury ingestion remains elevated because 76% and 85% of the fish evaluated, respectively, presented THQ ≥1. Jamanxim and Teles Pires sub-basins counted 8 (89%) and 28 (85%) species, respectively, potentially harmful for human consumption (Figure 7). A complete and detailed table with the minimum and maximum values for each species found in each sub-basin can be found in Table S10. For safe fish intake in the Tapajós river basin, we listed 32 species in at least one of the sub-basins for which a daily portion can exceed 116.25 g per capita, to a person with a body mass of 70 kg (Table S11).

## 4 Discussion

In this study we revised the literature on Hg pollution in the Tapajós river basin, period 1992–2022, focusing on the bioaccumulation in fish and the associated risk to human health via ingestion of contaminated species. Trends in Hg bioaccumulation was investigated from spatial (sub-basins) and ecological (trophic levels) perspectives. The phenomenon of environmental pollution in the Tapajós River linked to Hg disposal from gold mining operations has been well documented in the last decades, however, it is noteworthy that soils along the Tapajós Valley are naturally rich in Hg minerals and the land use changes coupled with biomass burden is the major drive of Hg mobilization in this region.^56,57^ The evidence presented in the scientific literature shows that several fish species are contaminated with high levels of mercury concentration (above 1 mg/kg) and such events can be recorded anywhere in the Tapajós basin. These pieces of evidence can be found in the literature: in lower Tapajós;^23,27,58,59^ in middle Tapajós;^60−62^ in upper Tapajós;^25,49,63^ in Jamanxim;^24^ in Teles Pires^64−66^ and Juruena.^47^

The qualitative analysis of the literature revealed high dissimilarity in the studies, resulting from large variation of sampling sizes, a minimum of one individual and a maximum of 75 individuals. The majority of the reported species were from studies with less than 15 individuals. Additionally, the sampling sites are not homogeneously distributed in the study area, being localized preferentially on the Tapajós riverbanks along the lower and middle river course. A large portion of the Tapajós river basin, delimited by the drainages of its main tributaries (Jamanxim, Teles Pires and Juruena) is poorly studied and virtually uncovered by scientific data. We interpret these results as evidence highlighting taxonomic and spatial gaps. Further studies should prioritize criteria for standardized sampling designs, we also recommend rigorous treatment for taxonomic species identifications, especially for groups with taxonomic issues, for example, the recent description of *Hoplias auri* as a distinct species from the *H. malabaricus* complex highlights the need for such rigorous approaches.^67^*H. auri* is recorded only in the Crepori River and is endemic to the Tapajós River basin.

The importance of accurate taxonomic assignments may be illustrated by the particular case of *“Cichlasoma spectabile”*, cited in Castilhos et al. (2015)^24^ from material collected in the Jamanxim sub-basin and presenting high levels of Hg concentration. Such a nominal taxon and its nomenclatural history could not be recovered in the specialized literature,^33^ which raised doubts about its taxonomic validity. Apart from the Castilhos study^24^ we only found this name reporting on fishes from Tocantins River basin.^68,69^ Due to this taxonomic confusion, we could not assign its trophic category and chose to exclude this taxon from the comparative Hg biomagnification analysis through the ecological hierarchical food chains is widely known in the literature^24,70^ and supports our observation of the highest mercury concentration in piscivorous fish from Tapajós basin. Spatial analysis indicates that middle/upper Tapajós and Teles Pires with the highest mercury contamination, and that more species had Hg concentration higher than 0.5 mg/kg, which is the safe limit determined by the Brazilian Agency (ANVISA), for nonpiscivorous fish. Therefore, we may assume these areas as “hotspots” of mercury contamination in the Tapajós basin. This spatial pattern is probably associated with Hg emissions derived from gold mining operations which are spread in the upper Tapajós, Jamanxim and Teles Pires sub-basins.^24,58,71^ Hosseini et al. (2013)^72^ found that mercury concentration in fish can be influenced by the habitat more than by the food habits. The significant increase of Hg in piscivorous and omnivorous fish from habitats affected by gold mines (Jamanxim sub-basin) corroborates Hosseini’s hypothesis.

It is possible to establish associations between mercury concentrations in fish not only based on their trophic level but also in relation to the influence of increasing mining activities in the Tapajós River basin, where the main tributaries have been impacted. Studies, such as those by Telmer et al. (2006),^73^ have demonstrated that the release of sediments into water bodies due to mining is associated with increases in Hg concentrations, both in particulate and dissolved forms, up to 600 times higher than in pristine areas. This increase is attributed to the high clay content in sediments and fine particles that remain suspended in the water for longer periods. The input of suspended sediments from mining activities in the Teles Pires, Creporí, and Jamanxim rivers creates a sediment plume that disperses along the Tapajós River basin. This sediment mixing can potentially alter mercury concentrations in specific locations, depending on factors such as geography, topography, and seasonal variations in mining activities or local mercury inputs. Such conditions may lead to the transport of sediments to lentic and/or shallow areas, where they promote methylation processes, making mercury more bioavailable to aquatic biota.^23,62,73−76^

The sub-basins of middle Tapajós, upper Tapajós, Jamanxin and Teles Pires showed a spatial pattern of mercury bioaccumulation, which can be observed in fish at levels 2 and 4. These regions are notably those with the highest concentration of “garimpos” and high population density, which in turn implies greater anthropogenic pressure on the territory, consequently leading to rapid processes of landscape change, often associated with deforestation and fires, both mechanisms acting in the transport of mercury to bodies of water. The anthropogenic emission of Hg and the mobilization of natural Hg present in the soil^56,57,77^ could enhance the processes of metabolization and bioavailability of mercury in the upper Tapajós, Jamanxim and Teles Pires sub-basins, which would explain the observed spatial pattern. Observations in our results, certain areas with mining activity show that TL4 organisms do not always exhibit high mercury concentrations (portions of the upper Tapajós and Teles Pires), whereas in areas without apparent mining activity, higher concentrations can occur. This suggests that the environmental characteristics of each location, such as sediment type, water flow, land use, and other local factors, play a role in the bioaccumulation of mercury in the biota, beyond just the direct input from mining. On the other hand, it is important to recognize that the lack of homogeneous spatiotemporal data, inconsistencies in data collection methods (such as lack of data such as organism size and weight), and the absence of a good taxonomic classification and continuous biomonitoring projects both in fish and considering general environmental parameters, could be hindering the establishment of clear cause-effect relationships over time. Since this is a literature review, we cannot standardize the data between the different sub-basins. Addressing these gaps through standardized practices and monitoring could improve our understanding of the dynamics, real effects, and intensity of risks associated with mercury contamination and exposure.

The temporal analyses conducted with the available data from 1992 to 2022, along with the number of articles published during this period, showed relatively weak associations without significant influence on the variations in THg concentrations. However, a difference was observed over time in the number of articles published: the first decade had the highest number of publications (*n* = 18) and 384 reported Hg values concentrations; the second decade had the fewest studies (*n* = 8) with 246 Hg reported values; and the third decade had ten studies with 105 Hg values. Throughout the study period, there has been a clear expansion of areas designated for artisanal gold mining^78,79^ and a trend of converting forested areas for agricultural and urban activities in the Tapajós region.^80−82^ These transformations are the main forces responsible for the mobilization of mercury in aquatic environments, suggesting that the bioaccumulation of this metal in fish should increase over time. However, in light of the analyzed bibliographic data, we did not detect any temporal variation in mercury bioaccumulation in fish from the Tapajós basin. Notably, organisms from TL1 were the only group to show temporal variations between the first decade (1992–2001) and the second decade (2002–2011), with the highest values (>0.3 mg/kg) reported in the second decade. This was observed in species such as *Shizodon* spp. and *M. duriventre*.

The lack of periodic data distribution over time is also reflected in its distribution among the sub-basins. A point-by-point data review indicates that samples are rarely collected at the same points in different periods, making it difficult to identify patterns of increase or decrease in THg values. Thus, it is not possible to perceive any trends in THg values, even when there are reports of an increase in human activities such as mining, burning, and the redistribution of land use for agriculture and livestock over time, in addition to the physicochemical environmental characteristics of the regions that affect mercury.

Considering the complete data set, nonpiscivorous species considered important for human consumption in riverine communities, e.g. *H. unimaculatus*, *P. blochii*, *Auchenipterus* sp., *O. bicirrhosum*, *Geophagus* sp. and *Hypophthalmus* sp., presented mercury concentrations above the limit established by regulatory agencies ANVISA and WHO, mainly in locations influenced by gold mining in the middle, upper Tapajós and Jamanxim sub-basins. This can be attributed, in part, to their feeding habits and the increased availability of mercury-associated particles and minerals in the water column resulting from higher turbidity near mining sites (with reduced forest cover). Additionally, the greater presence of aquatic plants may facilitate the adsorption and/or absorption of mercury on their surfaces.^62,74^ Although it is recommended to consume species at lower trophic levels, special care is required for these species due to the high levels of mercury found.

The biomonitoring of Hg in fish from Tapajós is critically important in the lower portion of the basin since this area has the largest population and a big urban center at Santarém. While piscivorous species (e.g., *Cichla* sp., *Pellona* sp., *Arapaima gigas*, *Brachyplatystoma vaillantii*) recorded low values of Hg bioaccumulation in this area; this is not the case for omnivorous *M. duriventre* and *S. fasciatus* (Bourdineaud et al. 2015). It is an intriguing observation because the primary sources of Hg emissions are to be more than 200 km upstream. However, Hg bioaccumulation analyzed from fish collected in Santarém may be biased, as the sampling approaches included specimens bought in markets (see Bourdineaud et al. 2015^23^). The Santarém fish markets commercialize species originating from several localities, even outside of Tapajós basin.^83^

Human exposure to mercury in the Amazon region is strongly associated with fish consumption.^24,25,84^ In many rural and peri-urban communities throughout the Amazon basin, fish is a crucial food source, especially among poorer socioeconomic strata and indigenous populations.^85^ One of the main challenges between monitoring human exposure to mercury and food security in riverine communities lies in understanding the heterogeneity and complexity of Hg bioaccumulation among different ecological groups of fish.^86^ Fish consumption patterns by Tapajós riverside populations are shaped by cultural, spatial, social differences, seasonality and fish availability.^49,51,87,88^

Health risk assessment consists of quantifying the probability of adverse effects on human health due to exposure to a specific toxic agent.^89^ Daily consumption can range from 8g per capita/day^50^ to 217g per capita/day.^25^ Although the values adopted as reference by regulatory agencies such as ANVISA and WHO are often cited as safe or unsafe levels for human consumption, these limits do not consider the adverse health effects produced by the ingestion of methylmercury in fish.^25^ Additionally, it is crucial to recognize that the amount of fish consumed by the population is as relevant as the concentration of mercury (Hg) present in the fish themselves. In regions of the Amazon, where fish consumption rates are high, a Risk Assessment that is based exclusively on the concentration of Hg in fish may underestimate the real amount of methylmercury (MeHg) ingested through the diet and, consequently, the risks to health associated with chronic exposure.^90^

In this study, high THQ values were found across all trophic levels, with only 14 species presenting THQ values <1 in the entire data set evaluated. When assessed separately by sub-basin, we observed that among these 14 species, only one acari species (*Hypostomus* sp.), was evaluated in more than one sub-basin and consistently maintained a THQ value <1. Furthermore, when considering the number of Hg values assessed, the sample size was always based on a single measurement. In cases where data were available for more than one sub-basin, at least one sub-basin showed elevated THQ indices (S9). These data suggest that the consumption of fish from the Tapajós River basin poses a potential health risk to local residents who consume these fish. However, we recognize that the estimation of human health risk based on this method may not accurately reflect the actual risk for the entire basin. For example, it is possible that urban populations, particularly those located in areas with higher population density (e.g., Santarém), consume less than 100 g per capita of fish per day, which could result in lower health risks. On the other hand, for riverside populations, even in localities with low population density, such as those living on the banks of the Tapajós River, consumption may exceed 200 g per capita per day, increasing health risks and making the situation more severe than described in this study. For this reason, considering the complexity of the issue, these results should be interpreted with caution.

The fish species consumed in the Tapajós River basin are variable, and the exact proportion of each species is not known. Among the most consumed fish in the Tapajós River basin are piscivorous fish such as tucunaré (*Cichla* spp.), surubim (*Pseudoplatystoma* spp.), piraíba (*B. filamentosum*), pescada (*Plagioscion* spp.), apapá (*Pellona* spp.), pirarucu (*A. gigas*), and nonpiscivorous fish such as jaraqui (*Semaprochilodus* spp.), caratinga (*Geophagus* spp.), curimatá (*Prochilodus* spp.), pacu (*Mylossoma* spp.).^25,49,51,87,91^ Piscivorous species had average mercury levels ranging from 0.03 to 3.82 mg/kg in muscle. In contrast, mercury levels of 0.01 to 0.98 mg/kg were found in nonpiscivorous species. In this study, we present an updated table of the safe quantity in grams for each trophic level and each subunit of the Tapajós River basin. In general, considering the average per capita daily fish consumption in the basin (116.25 g), 75% of the evaluated species should be consumed in smaller quantities in at least one of the analyzed sub-basins to avoid health risks. Among the evaluated trophic levels, all the piscivorous species (*n* = 54) should be consumed below 116.25 g, even if the Hg levels in some species are below the recommended international guidelines. Thus, opting for nonpiscivorous fish species would be more suitable for human consumption as they present lower contamination levels.

## 5 Conclusion

This review provides an update on mercury (Hg) human exposure and fish bioaccumulation in the Tapajós river basin. After three decades of scientific investigations some important issues could be clearly stated: (1) there is a spatial bias in the sampling, where most of the studies concentrate on middle/upper Tapajós, while the largest area of the drainage basin including lower Tapajós, Jamanxin, Juruena and Teles Pires were undersampled; (2) piscivorous fish (TL4) are the most contaminated and show spatial correlation along the study area; in this group *Cichla* spp. (tucunaré) and *Hoplias* spp. (traíras) were the species most investigated and may constitute suitable indicator organisms for basin Hg monitoring; (3) the regular consumption of piscivorous species poses a risk of Hg poisoning for the human population from the Tapajós basin.

To advance scientific knowledge about mercury contamination in the Tapajós River basin, it is essential to establish a permanent monitoring program for mercury bioaccumulation that employs standardized sampling and involves the active participation of the population nongovernment and government agencies (citizen science). Additionally, the role of local higher education institutions should be strengthened as analytical centers, integrating multidisciplinary teams that can address the issue comprehensively. Finally, it is essential to induce the creation of public policies that establish an exclusive fund to finance research and environmental monitoring related to mercury pollution in the Tapajós River basin. These actions will ensure the collection of more representative data and may foster community awareness and engagement, resulting in a more effective approach to tackling the challenges of mercury contamination in the Tapajós River basin. Spatial analysis of critical contamination areas in relation to important fish habitats can provide the tools needed to mitigate these threats. We propose that future research and monitoring programs prioritize a more equitable distribution of samples throughout the Tapajós River basin, considering not only areas impacted by human activities, such as mining, but also areas that are less explored and potentially vulnerable to mercury contamination. We recommend that the consumption of piscivorous species be strictly monitored and regulated by competent public governmental agencies. This precaution can protect and contribute to public health, especially for populations living on the banks of the Tapajós River and close to gold mining in the basin.

## Supporting Information Available

Flow diagram of the study selection process; data extracted from eligible bibliographic sources including site, coordinates, year, species, trophic level, sample size, mean mercury concentration, and first author; classification of areas to compare sub-basins in the Tapajós River basin; number of individuals analyzed per species and sub-basin; Hg concentration in fishes from Tapajós river basin by trophic level and sub-basin; spatial autocorrelation analysis of Hg bioaccumulation in piscivorous fish; range values (maximum–minimum) of Hg concentration by trophic level and sub-basin; Hg measurement counts and publication trends across decades; statistical analysis of Hg concentration variations by trophic level; Hazard Quotient (THQ) evaluation values for human health risk; and safe daily consumption quantity in grams for 129 fish species (PDF)
