# Supplementary material for: Systematic Review and Spatiotemporal Assessment of Mercury Concentration in Fish from the Tapajós River Basin: Implications for Environmental and Human Health
Source: ACS Environ Au. 2024 Oct 30;5(1):86–100. doi: 10.1021/acsenvironau.4c00053 (PMC11741060; doi:10.1021/acsenvironau.4c00053)
Supplement: Supplementary file 1 — vg4c00053_si_001.pdf [file vg4c00053_si_001.pdf]

Supporting Information

Systematic review and spatiotemporal assessment of mercury concentration in fish from the Tapajós River Basin: implications for environmental and human health

Karen L. Auzier Guimarães[a,b,*], Sarah J. do Nascimento Andrade[b], Ahieska A. Liscano-Carreño[a,b,c], Ricardo B. de Oliveira[d], Luís R. Ribeiro Rodrigues[a,b]

[a] Programa de Pós-Graduação em Biodiversidade e Biotecnologia (REDE BIONORTE), Instituto de Saúde Coletiva (ISCO), Universidade Federal do Oeste do Pará (UFOPA), Rua Vera Paz, s/nº, Salé, CEP 68040-255, Santarém, Pará, Brazil.
[b] Laboratório de Genética & Biodiversidade (LGBio), Instituto de Ciências da Educação (ICED), Universidade Federal do Oeste do Pará (UFOPA), Rua Vera Paz, s/nº, Salé, CEP 68040-255, Santarém, Pará, Brazil.
[c] Departamento de Biología, Universidad de Oriente (UDO), Avenida Universidad, s/n°, Cod Postal 6101, Cumaná, Sucre, Venezuela
[d] Laboratório de Bioprospecção e Biologia Experimental, Instituto de Ciências da Educação (ICED), Universidade Federal do Oeste do Pará (UFOPA), Rua Vera Paz, s/nº, Salé, CEP 68040-255, Santarém, Pará, Brazil.
[*] Corresponding author: karen.guimaraes.bio@gmail.com

The Supporting Information contains figures and tables showing the flow diagram of the study selection process (Figure S1), data extracted from eligible bibliographic sources including site, coordinates, year, species, trophic level, sample size, mean mercury concentration, and first author (Table S2), classification of areas to compare sub-basins in the Tapajós River basin (Figure S3), number of individuals analyzed per species and sub-basin (Table S4), Hg concentration in fishes from Tapajós river basin by trophic level and sub-basin (Figure S5), spatial autocorrelation analysis of Hg bioaccumulation in piscivorous fish (Figure S6), range values (maximum-minimum) of Hg concentration by trophic level and sub-basin (Table S7), Hg measurement counts and publication trends across decades (Table S8), statistical analysis of Hg concentration variations by trophic level (Table S9), Hazard Quotient (THQ) evaluation values for human health risk (Table S10), and safe daily consumption quantity in grams for 129 fish species (Table S11).

Figure S1. Flow diagram (PRISMA format) of study selection process.

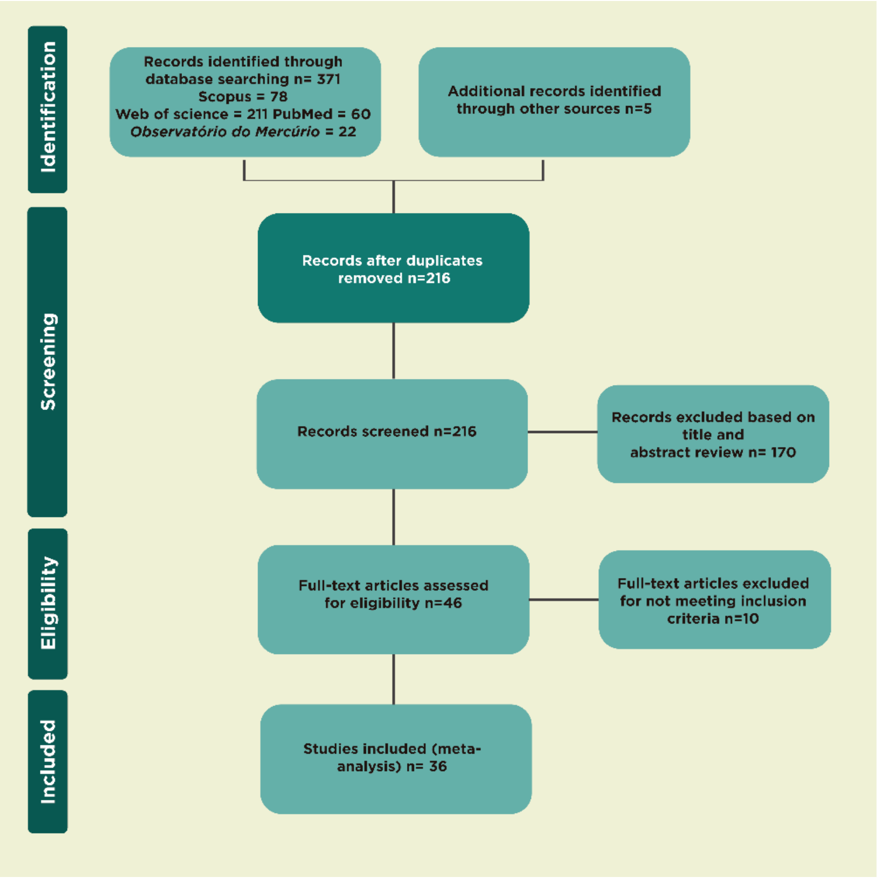

Table S2. Data extracted from eligible bibliographic sources, including collection site, geographical coordinates, sampling year, species, trophic level, sample size, mean mercury concentration, and first author's name.

**Collection location**	**Sub-basin**	**Lat S**	**Long W**	**Year**	***Species***	**Trophic level**	**N**	**Mercury concentration (mg/kg)**	**Reference**
Barreiras	Middle Tapajós	-4,09608	-55,68831	2014	*Brachyplatystoma filamentosum*	4	3	0,69	Faial et al. 2014
Barreiras	Middle Tapajós	-4,09608	-55,68831	2014	*Plagioscion squamosissimus*	4	75	0,68	Faial et al. 2014
Barreiras	Middle Tapajós	-4,09608	-55,68831	2014	*Calophysus macropterus*	4	14	0,58	Faial et al. 2014
Barreiras	Middle Tapajós	-4,09608	-55,68831	2014	*Cichla sp.*	4	13	0,64	Faial et al. 2014
Barreiras	Middle Tapajós	-4,09608	-55,68831	2014	*Leporinus sp.*	2	46	0,12	Faial et al. 2014
Barreiras	Middle Tapajós	-4,09608	-55,68831	2014	*Geophagus brasiliensis*	3	21	0,11	Faial et al. 2014
Barreiras	Middle Tapajós	-4,09608	-55,68831	2014	*Hemiodus sp.*	2	3	0,09	Faial et al. 2014
Barreiras	Middle Tapajós	-4,09608	-55,68831	2014	*Mylossoma sp.*	1	55	0,05	Faial et al. 2014
Barreiras	Middle Tapajós	-4,09608	-55,68831	2014	*Astronotus crassipinnis*	2	2	0,34	Faial et al. 2014
São Luis do Tapajós	Middle Tapajós	-4,46011	-56,24997	2003	*Plagioscion sp.*	4		0,65	Passos et al. 2008
Nova Canaã	Middle Tapajós	-4,36965	-56,23723	2003	*Plagioscion sp.*	4		0,57	Passos et al. 2008
Santo Antônio	Middle Tapajós	-4,25169	-55,91727	2003	*Plagioscion sp.*	4		0,60	Passos et al. 2008
Vista Alegre	Middle Tapajós	-3,6832	-55,39722	2003	*Plagioscion sp.*	4		0,45	Passos et al. 2008
Mussum	Middle Tapajós	-3,68619	-55,41147	2003	*Plagioscion sp.*	4		0,45	Passos et al. 2008
Açaituba	Middle Tapajós	-3,8275	-55,35698	2003	*Plagioscion sp.*	4		0,39	Passos et al. 2008
São Luis do Tapajós	Middle Tapajós	-4,46011	-56,24997	2003	*Schizodon sp.*	1		0,20	Passos et al. 2008
Nova Canaã	Middle Tapajós	-4,36965	-56,23723	2003	*Schizodon sp.*	1		0,17	Passos et al. 2008
Santo Antônio	Middle Tapajós	-4,25169	-55,91727	2003	*Schizodon sp.*	1		0,13	Passos et al. 2008
Vista Alegre	Middle Tapajós	-3,6832	-55,39722	2003	*Schizodon sp.*	1		0,10	Passos et al. 2008
Mussum	Middle Tapajós	-3,68619	-55,41147	2003	*Schizodon sp.*	1		0,10	Passos et al. 2008
Açaituba	Middle Tapajós	-3,8275	-55,35698	2003	*Schizodon sp.*	1		0,14	Passos et al. 2008
São Luis do Tapajós	Middle Tapajós	-4,46011	-56,24997	2003	*Geophagus sp.*	3		0,18	Passos et al. 2008
Nova Canaã	Middle Tapajós	-4,36965	-56,23723	2003	*Geophagus sp.*	3		0,18	Passos et al. 2008
Santo Antônio	Middle Tapajós	-4,25169	-55,91727	2003	*Geophagus sp.*	3		0,14	Passos et al. 2008

Vista Alegre	Middle Tapajós	-3,6832	-55,39722	2003	*Geophagus sp.*	3		0,07	Passos et al. 2008
Mussum	Middle Tapajós	-3,68619	-55,41147	2003	*Geophagus sp.*	3		0,07	Passos et al. 2008
Açaituba	Middle Tapajós	-3,8275	-55,35698	2003	*Geophagus sp.*	3		0,11	Passos et al. 2008
São Luis do Tapajós	Middle Tapajós	-4,46011	-56,24997	2003	*Cichla sp.*	4		2,30	Passos et al. 2008
Nova Canaã	Middle Tapajós	-4,36965	-56,23723	2003	*Cichla sp.*	4		0,60	Passos et al. 2008
Santo Antônio	Middle Tapajós	-4,25169	-55,91727	2003	*Cichla sp.*	4		0,39	Passos et al. 2008
Vista Alegre	Middle Tapajós	-3,6832	-55,39722	2003	*Cichla sp.*	4		0,52	Passos et al. 2008
Mussum	Middle Tapajós	-3,68619	-55,41147	2003	*Cichla sp.*	4		0,52	Passos et al. 2008
Açaituba	Middle Tapajós	-3,8275	-55,35698	2003	*Cichla sp.*	4		0,19	Passos et al. 2008
São Luis do Tapajós	Middle Tapajós	-4,46011	-56,24997	2003	*Pellona sp.*	4		0,43	Passos et al. 2008
Nova Canaã	Middle Tapajós	-4,36965	-56,23723	2003	*Pellona sp.*	4		0,61	Passos et al. 2008
Santo Antônio	Middle Tapajós	-4,25169	-55,91727	2003	*Pellona sp.*	4		0,66	Passos et al. 2008
Vista Alegre	Middle Tapajós	-3,6832	-55,39722	2003	*Pellona sp.*	4		0,77	Passos et al. 2008
Mussum	Middle Tapajós	-3,68619	-55,41147	2003	*Pellona sp.*	4		0,77	Passos et al. 2008
Açaituba	Middle Tapajós	-3,8275	-55,35698	2003	*Pellona sp.*	4		0,66	Passos et al. 2008
São Luis do Tapajós	Middle Tapajós	-4,46011	-56,24997	2003	*Serrasalmus sp.*	4		0,40	Passos et al. 2008
Nova Canaã	Middle Tapajós	-4,36965	-56,23723	2003	*Serrasalmus sp.*	4		0,57	Passos et al. 2008
Santo Antônio	Middle Tapajós	-4,25169	-55,91727	2003	*Serrasalmus sp.*	4		0,40	Passos et al. 2008
Vista Alegre	Middle Tapajós	-3,6832	-55,39722	2003	*Serrasalmus sp.*	4		0,40	Passos et al. 2008
Mussum	Middle Tapajós	-3,68619	-55,41147	2003	*Serrasalmus sp.*	4		0,40	Passos et al. 2008
Açaituba	Middle Tapajós	-3,8275	-55,35698	2003	*Serrasalmus sp.*	4		0,31	Passos et al. 2008
São Luis do Tapajós	Middle Tapajós	-4,46011	-56,24997	2003	*Curimata sp.*	1		0,19	Passos et al. 2008
Nova Canaã	Middle Tapajós	-4,36965	-56,23723	2003	*Curimata sp.*	1		0,17	Passos et al. 2008
Santo Antônio	Middle Tapajós	-4,25169	-55,91727	2003	*Curimata sp.*	1		0,10	Passos et al. 2008
Vista Alegre	Middle Tapajós	-3,6832	-55,39722	2003	*Curimata sp.*	1		0,06	Passos et al. 2008
Mussum	Middle Tapajós	-3,68619	-55,41147	2003	*Curimata sp.*	1		0,06	Passos et al. 2008
Açaituba	Middle Tapajós	-3,8275	-55,35698	2003	*Curimata sp.*	1		0,07	Passos et al. 2008

São Luis do Tapajós	Middle Tapajós	-4,46011	-56,24997	2003	*Hemiodus sp.*	2		0,09	Passos et al. 2008
Nova Canaã	Middle Tapajós	-4,36965	-56,23723	2003	*Hemiodus sp.*	2		0,08	Passos et al. 2008
Santo Antônio	Middle Tapajós	-4,25169	-55,91727	2003	*Hemiodus sp.*	2		0,14	Passos et al. 2008
Vista Alegre	Middle Tapajós	-3,6832	-55,39722	2003	*Hemiodus sp.*	2		0,05	Passos et al. 2008
Mussum	Middle Tapajós	-3,68619	-55,41147	2003	*Hemiodus sp.*	2		0,05	Passos et al. 2008
Açaituba	Middle Tapajós	-3,8275	-55,35698	2003	*Hemiodus sp.*	2		0,07	Passos et al. 2008
São Luis do Tapajós	Middle Tapajós	-4,46011	-56,24997	2003	*Auchenipterus sp.*	3		0,46	Passos et al. 2008
Nova Canaã	Middle Tapajós	-4,36965	-56,23723	2003	*Auchenipterus sp.*	3		0,33	Passos et al. 2008
Santo Antônio	Middle Tapajós	-4,25169	-55,91727	2003	*Auchenipterus sp.*	3		0,41	Passos et al. 2008
Vista Alegre	Middle Tapajós	-3,6832	-55,39722	2003	*Auchenipterus sp.*	3		0,69	Passos et al. 2008
Mussum	Middle Tapajós	-3,68619	-55,41147	2003	*Auchenipterus sp.*	3		0,69	Passos et al. 2008
Açaituba	Middle Tapajós	-3,8275	-55,35698	2003	*Auchenipterus sp.*	3		0,20	Passos et al. 2008
São Luis do Tapajós	Middle Tapajós	-4,46011	-56,24997	2003	*Semaprochilodus sp.*	1		0,06	Passos et al. 2008
Nova Canaã	Middle Tapajós	-4,36965	-56,23723	2003	*Semaprochilodus sp.*	1		0,09	Passos et al. 2008
Açaituba	Middle Tapajós	-3,8275	-55,35698	2003	*Semaprochilodus sp.*	1		0,05	Passos et al. 2008
São Luis do Tapajós	Middle Tapajós	-4,46011	-56,24997	2003	*Pseudoplatystoma sp.*	4		0,28	Passos et al. 2008
Nova Canaã	Middle Tapajós	-4,36965	-56,23723	2003	*Pseudoplatystoma sp.*	4		0,08	Passos et al. 2008
Açaituba	Middle Tapajós	-3,8275	-55,35698	2003	*Pseudoplatystoma sp.*	4		0,07	Passos et al. 2008
São Luis do Tapajós	Middle Tapajós	-4,46011	-56,24997	2003	*Rhaphiodon vulpinus*	4		0,63	Passos et al. 2008
Nova Canaã	Middle Tapajós	-4,36965	-56,23723	2003	*Rhaphiodon vulpinus*	4		0,58	Passos et al. 2008
Santo Antônio	Middle Tapajós	-4,25169	-55,91727	2003	*Rhaphiodon vulpinus*	4		0,91	Passos et al. 2008
Açaituba	Middle Tapajós	-3,8275	-55,35698	2003	*Rhaphiodon vulpinus*	4		0,42	Passos et al. 2008
São Luis do Tapajós	Middle Tapajós	-4,46011	-56,24997	2003	*Brycon sp.*	2		0,01	Passos et al. 2008
São Luis do Tapajós	Middle Tapajós	-4,46011	-56,24997	2003	*Mylossoma sp.*	1		0,02	Passos et al. 2008
Nova Canaã	Middle Tapajós	-4,36965	-56,23723	2003	*Mylossoma sp.*	1		0,02	Passos et al. 2008
Açaituba	Middle Tapajós	-3,8275	-55,35698	2003	*Mylossoma sp.*	1		0,02	Passos et al. 2008
Açaituba	Middle Tapajós	-3,8275	-55,35698	2003	*Colossoma macropomum*	1		0,04	Passos et al. 2008

Nova Canaã	Middle Tapajós	-4,36965	-56,23723	2003	*Pachypops sp.*	2		0,01	Passos et al. 2008
Nova Canaã	Middle Tapajós	-4,36965	-56,23723	2003	*Hoplias sp.*	4		0,03	Passos et al. 2008
Açaituba	Middle Tapajós	-3,8275	-55,35698	2003	*Hoplias sp.*	4		0,04	Passos et al. 2008
Açaituba	Middle Tapajós	-3,8275	-55,35698	2003	*Prochilodus sp.*	1		0,01	Passos et al. 2008
Nova Canaã	Middle Tapajós	-4,36965	-56,23723	2003	*Triportheus sp.*	2		0,02	Passos et al. 2008
Açaituba	Middle Tapajós	-3,8275	-55,35698	2003	*Triportheus sp.*	2		0,03	Passos et al. 2008
São Luis do Tapajós	Middle Tapajós	-4,46011	-56,24997	2003	*Osteoglossum bicirrhosum*	3		0,20	Passos et al. 2008
Santo Antônio	Middle Tapajós	-4,25169	-55,91727	2003	*Osteoglossum bicirrhosum*	3		0,80	Passos et al. 2008
Açaituba	Middle Tapajós	-3,8275	-55,35698	2003	*Osteoglossum bicirrhosum*	3		0,30	Passos et al. 2008
Nova Canaã	Middle Tapajós	-4,36965	-56,23723	2003	*Pterygoplichthys pardalis*	1		0,10	Passos et al. 2008
Santo Antônio	Middle Tapajós	-4,25169	-55,91727	2003	*Pterygoplichthys pardalis*	1		0,10	Passos et al. 2008
Açaituba	Middle Tapajós	-3,8275	-55,35698	2003	*Pterygoplichthys pardalis*	1		0,04	Passos et al. 2008
São Luis do Tapajós	Middle Tapajós	-4,46011	-56,24997	2003	*Hypophthalmus sp.*	3		0,10	Passos et al. 2008
Nova Canaã	Middle Tapajós	-4,36965	-56,23723	2003	*Hypophthalmus sp.*	3		0,20	Passos et al. 2008
Vista Alegre	Middle Tapajós	-3,6832	-55,39722	2003	*Hypophthalmus sp.*	3		0,30	Passos et al. 2008
Mussum	Middle Tapajós	-3,68619	-55,41147	2003	*Hypophthalmus sp.*	3		0,30	Passos et al. 2008
Açaituba	Middle Tapajós	-3,8275	-55,35698	2003	*Oxydoras sp.*	3		0,20	Passos et al. 2008
Nova Canaã	Middle Tapajós	-4,36965	-56,23723	2003	*Crenicichla sp.*	4		0,50	Passos et al. 2008
Cupari	Middle Tapajós	-4,68544	-55,41125	2004	*Curimata inornata*	1	2	0,10	Sampaio da Silva et al. 2009
Cupari	Middle Tapajós	-4,68544	-55,41125	2004	*Curimata inornata*	1	9	0,20	Sampaio da Silva et al. 2009
Cupari	Middle Tapajós	-4,68544	-55,41125	2004	*Geophagus proximus*	3	7	0,11	Sampaio da Silva et al. 2009
Cupari	Middle Tapajós	-4,68544	-55,41125	2004	*Geophagus proximus*	3	11	0,20	Sampaio da Silva et al. 2009
Cupari	Middle Tapajós	-4,68544	-55,41125	2004	*Leporinus fasciatus*	2	2	0,07	Sampaio da Silva et al. 2009
Cupari	Middle Tapajós	-4,68544	-55,41125	2004	*Leporinus fasciatus*	2	6	0,14	Sampaio da Silva et al. 2009
Cupari	Middle Tapajós	-4,68544	-55,41125	2004	*Schizodon vittatus*	1	3	0,18	Sampaio da Silva et al. 2009
Cupari	Middle Tapajós	-4,68544	-55,41125	2004	*Schizodon vittatus*	1	1	0,01	Sampaio da Silva et al. 2009
Cupari	Middle Tapajós	-4,68544	-55,41125	2004	*Anostomoides laticeps*	3	2	0,10	Sampaio da Silva et al. 2009

Cupari	Middle Tapajós	-4,68544	-55,41125	2004	*Caenotropus labyrinthicus*	2	10	0,39	Sampaio da Silva et al. 2009
Cupari	Middle Tapajós	-4,68544	-55,41125	2004	*Hemiodus unimaculatus*	2	42	0,07	Sampaio da Silva et al. 2009
Cupari	Middle Tapajós	-4,68544	-55,41125	2004	*Hemiodus unimaculatus*	2	19	0,13	Sampaio da Silva et al. 2009
Cupari	Middle Tapajós	-4,68544	-55,41125	2004	*Hoplias malabaricus*	4	4	0,40	Sampaio da Silva et al. 2009
Cupari	Middle Tapajós	-4,68544	-55,41125	2004	*Acestrorhynchus falcirostris*	4	11	0,74	Sampaio da Silva et al. 2009
Cupari	Middle Tapajós	-4,68544	-55,41125	2004	*Acestrorhynchus falcirostris*	4	1	0,42	Sampaio da Silva et al. 2009
Cupari	Middle Tapajós	-4,68544	-55,41125	2004	*Plagioscion squamosissimus*	4	17	0,39	Sampaio da Silva et al. 2009
Cupari	Middle Tapajós	-4,68544	-55,41125	2004	*Plagioscion squamosissimus*	4	6	0,66	Sampaio da Silva et al. 2009
Itapacurazinho	Middle Tapajós	-4,26864	-55,91003	2004	*Curimata inornata*	1	5	0,09	Sampaio da Silva et al. 2009
Itapacurazinho	Middle Tapajós	-4,26864	-55,91003	2004	*Curimata inornata*	1	17	0,10	Sampaio da Silva et al. 2009
Itapacurazinho	Middle Tapajós	-4,26864	-55,91003	2004	*Geophagus proximus*	3	4	0,14	Sampaio da Silva et al. 2009
Itapacurazinho	Middle Tapajós	-4,26864	-55,91003	2004	*Geophagus proximus*	3	18	0,26	Sampaio da Silva et al. 2009
Itapacurazinho	Middle Tapajós	-4,26864	-55,91003	2004	*Leporinus fasciatus*	2	2	0,16	Sampaio da Silva et al. 2009
Itapacurazinho	Middle Tapajós	-4,26864	-55,91003	2004	*Leporinus fasciatus*	2	2	0,30	Sampaio da Silva et al. 2009
Itapacurazinho	Middle Tapajós	-4,26864	-55,91003	2004	*Schizodon vittatus*	1	2	0,16	Sampaio da Silva et al. 2009
Itapacurazinho	Middle Tapajós	-4,26864	-55,91003	2004	*Schizodon vittatus*	1	3	0,30	Sampaio da Silva et al. 2009
Itapacurazinho	Middle Tapajós	-4,26864	-55,91003	2004	*Anostomoides laticeps*	3	13	0,19	Sampaio da Silva et al. 2009
Itapacurazinho	Middle Tapajós	-4,26864	-55,91003	2004	*Anostomoides laticeps*	3	7	0,46	Sampaio da Silva et al. 2009
Itapacurazinho	Middle Tapajós	-4,26864	-55,91003	2004	*Caenotropus labyrinthicus*	2	18	0,33	Sampaio da Silva et al. 2009
Itapacurazinho	Middle Tapajós	-4,26864	-55,91003	2004	*Caenotropus labyrinthicus*	2	5	0,32	Sampaio da Silva et al. 2009
Itapacurazinho	Middle Tapajós	-4,26864	-55,91003	2004	*Hemiodus unimaculatus*	2	48	0,14	Sampaio da Silva et al. 2009
Itapacurazinho	Middle Tapajós	-4,26864	-55,91003	2004	*Hemiodus unimaculatus*	2	20	0,17	Sampaio da Silva et al. 2009
Itapacurazinho	Middle Tapajós	-4,26864	-55,91003	2004	*Hoplias malabaricus*	4	12	1,40	Sampaio da Silva et al. 2009
Itapacurazinho	Middle Tapajós	-4,26864	-55,91003	2004	*Acestrorhynchus falcirostris*	4	15	0,87	Sampaio da Silva et al. 2009
Itapacurazinho	Middle Tapajós	-4,26864	-55,91003	2004	*Acestrorhynchus falcirostris*	4	24	1,66	Sampaio da Silva et al. 2009
Itapacurazinho	Middle Tapajós	-4,26864	-55,91003	2004	*Plagioscion squamosissimus*	4	9	0,60	Sampaio da Silva et al. 2009
Itapacurazinho	Middle Tapajós	-4,26864	-55,91003	2004	*Plagioscion squamosissimus*	4	2	1,37	Sampaio da Silva et al. 2009

Jacare	Middle Tapajós	-4,37136	-56,24353	2004	*Curimata inornata*	1	63	0,18	Sampaio da Silva et al. 2009
Jacare	Middle Tapajós	-4,37136	-56,24353	2004	*Curimata inornata*	1	28	0,11	Sampaio da Silva et al. 2009
Jacare	Middle Tapajós	-4,37136	-56,24353	2004	*Geophagus proximus*	3	16	0,18	Sampaio da Silva et al. 2009
Jacare	Middle Tapajós	-4,37136	-56,24353	2004	*Geophagus proximus*	3	7	0,16	Sampaio da Silva et al. 2009
Jacare	Middle Tapajós	-4,37136	-56,24353	2004	*Leporinus fasciatus*	2	18	0,15	Sampaio da Silva et al. 2009
Jacare	Middle Tapajós	-4,37136	-56,24353	2004	*Leporinus fasciatus*	2	2	0,15	Sampaio da Silva et al. 2009
Jacare	Middle Tapajós	-4,37136	-56,24353	2004	*Schizodon vittatus*	1	11	0,23	Sampaio da Silva et al. 2009
Jacare	Middle Tapajós	-4,37136	-56,24353	2004	*Schizodon vittatus*	1	15	0,09	Sampaio da Silva et al. 2009
Jacare	Middle Tapajós	-4,37136	-56,24353	2004	*Anostomoides laticeps*	3	6	0,23	Sampaio da Silva et al. 2009
Jacare	Middle Tapajós	-4,37136	-56,24353	2004	*Anostomoides laticeps*	3	2	0,23	Sampaio da Silva et al. 2009
Jacare	Middle Tapajós	-4,37136	-56,24353	2004	*Caenotropus labyrinthicus*	2	27	0,28	Sampaio da Silva et al. 2009
Jacare	Middle Tapajós	-4,37136	-56,24353	2004	*Caenotropus labyrinthicus*	2	13	0,26	Sampaio da Silva et al. 2009
Jacare	Middle Tapajós	-4,37136	-56,24353	2004	*Hemiodus unimaculatus*	2	25	0,08	Sampaio da Silva et al. 2009
Jacare	Middle Tapajós	-4,37136	-56,24353	2004	*Hemiodus unimaculatus*	2	31	0,06	Sampaio da Silva et al. 2009
Jacare	Middle Tapajós	-4,37136	-56,24353	2004	*Hoplias malabaricus*	4	1	0,27	Sampaio da Silva et al. 2009
Jacare	Middle Tapajós	-4,37136	-56,24353	2004	*Hoplias malabaricus*	4	5	0,81	Sampaio da Silva et al. 2009
Jacare	Middle Tapajós	-4,37136	-56,24353	2004	*Acestrorhynchus falcirostris*	4	3	0,67	Sampaio da Silva et al. 2009
Jacare	Middle Tapajós	-4,37136	-56,24353	2004	*Acestrorhynchus falcirostris*	4	12	1,18	Sampaio da Silva et al. 2009
Jacare	Middle Tapajós	-4,37136	-56,24353	2004	*Plagioscion squamosissimus*	4	31	0,57	Sampaio da Silva et al. 2009
Jacare	Middle Tapajós	-4,37136	-56,24353	2004	*Plagioscion squamosissimus*	4	23	0,53	Sampaio da Silva et al. 2009
Parana	Middle Tapajós	-4,45811	-56,24994	2004	*Curimata inornata*	1	15	0,12	Sampaio da Silva et al. 2009
Parana	Middle Tapajós	-4,45811	-56,24994	2004	*Geophagus proximus*	3	4	0,18	Sampaio da Silva et al. 2009
Parana	Middle Tapajós	-4,45811	-56,24994	2004	*Geophagus proximus*	3	11	0,10	Sampaio da Silva et al. 2009
Parana	Middle Tapajós	-4,45811	-56,24994	2004	*Leporinus fasciatus*	2	25	0,18	Sampaio da Silva et al. 2009
Parana	Middle Tapajós	-4,45811	-56,24994	2004	*Leporinus fasciatus*	2	1	0,13	Sampaio da Silva et al. 2009
Parana	Middle Tapajós	-4,45811	-56,24994	2004	*Schizodon vittatus*	1	10	0,19	Sampaio da Silva et al. 2009
Parana	Middle Tapajós	-4,45811	-56,24994	2004	*Schizodon vittatus*	1	5	0,13	Sampaio da Silva et al. 2009

Parana	Middle Tapajós	-4,45811	-56,24994	2004	*Anostomoides laticeps*	3	12	0,26	Sampaio da Silva et al. 2009
Parana	Middle Tapajós	-4,45811	-56,24994	2004	*Caenotropus labyrinthicus*	2	19	0,25	Sampaio da Silva et al. 2009
Parana	Middle Tapajós	-4,45811	-56,24994	2004	*Hemiodus unimaculatus*	2	46	0,09	Sampaio da Silva et al. 2009
Parana	Middle Tapajós	-4,45811	-56,24994	2004	*Hemiodus unimaculatus*	2	9	0,07	Sampaio da Silva et al. 2009
Parana	Middle Tapajós	-4,45811	-56,24994	2004	*Hoplias malabaricus*	4	15	0,30	Sampaio da Silva et al. 2009
Parana	Middle Tapajós	-4,45811	-56,24994	2004	*Acestrorhynchus falcirostris*	4	2	0,76	Sampaio da Silva et al. 2009
Parana	Middle Tapajós	-4,45811	-56,24994	2004	*Acestrorhynchus falcirostris*	4	12	0,50	Sampaio da Silva et al. 2009
Parana	Middle Tapajós	-4,45811	-56,24994	2004	*Plagioscion squamosissimus*	4	75	0,65	Sampaio da Silva et al. 2009
Parana	Middle Tapajós	-4,45811	-56,24994	2004	*Plagioscion squamosissimus*	4	8	0,47	Sampaio da Silva et al. 2009
Restinga	Middle Tapajós	-3,8285	-55,35589	2004	*Curimata inornata*	1	28	0,05	Sampaio da Silva et al. 2009
Restinga	Middle Tapajós	-3,8285	-55,35589	2004	*Curimata inornata*	1	20	0,03	Sampaio da Silva et al. 2009
Restinga	Middle Tapajós	-3,8285	-55,35589	2004	*Geophagus proximus*	3	25	0,07	Sampaio da Silva et al. 2009
Restinga	Middle Tapajós	-3,8285	-55,35589	2004	*Geophagus proximus*	3	16	0,08	Sampaio da Silva et al. 2009
Restinga	Middle Tapajós	-3,8285	-55,35589	2004	*Leporinus fasciatus*	2	17	0,11	Sampaio da Silva et al. 2009
Restinga	Middle Tapajós	-3,8285	-55,35589	2004	*Schizodon vittatus*	1	23	0,11	Sampaio da Silva et al. 2009
Restinga	Middle Tapajós	-3,8285	-55,35589	2004	*Schizodon vittatus*	1	2	0,21	Sampaio da Silva et al. 2009
Restinga	Middle Tapajós	-3,8285	-55,35589	2004	*Anostomoides laticeps*	3	3	0,12	Sampaio da Silva et al. 2009
Restinga	Middle Tapajós	-3,8285	-55,35589	2004	*Anostomoides laticeps*	3	33	0,12	Sampaio da Silva et al. 2009
Restinga	Middle Tapajós	-3,8285	-55,35589	2004	*Caenotropus labyrinthicus*	2	6	0,16	Sampaio da Silva et al. 2009
Restinga	Middle Tapajós	-3,8285	-55,35589	2004	*Caenotropus labyrinthicus*	2	38	0,09	Sampaio da Silva et al. 2009
Restinga	Middle Tapajós	-3,8285	-55,35589	2004	*Hemiodus unimaculatus*	2	66	0,04	Sampaio da Silva et al. 2009
Restinga	Middle Tapajós	-3,8285	-55,35589	2004	*Hemiodus unimaculatus*	2	2	0,12	Sampaio da Silva et al. 2009
Restinga	Middle Tapajós	-3,8285	-55,35589	2004	*Hoplias malabaricus*	4	8	0,50	Sampaio da Silva et al. 2009
Restinga	Middle Tapajós	-3,8285	-55,35589	2004	*Acestrorhynchus falcirostris*	4	1	0,20	Sampaio da Silva et al. 2009
Restinga	Middle Tapajós	-3,8285	-55,35589	2004	*Acestrorhynchus falcirostris*	4	45	0,48	Sampaio da Silva et al. 2009
Restinga	Middle Tapajós	-3,8285	-55,35589	2004	*Plagioscion squamosissimus*	4	12	0,45	Sampaio da Silva et al. 2009
Restinga	Middle Tapajós	-3,8285	-55,35589	2004	*Plagioscion squamosissimus*	4	14	0,55	Sampaio da Silva et al. 2009

Capitua	Middle Tapajós	-4,33219	-56,07897	2004	*Geophagus proximus*	3	6	0,18	Sampaio da Silva et al. 2009
Capitua	Middle Tapajós	-4,33219	-56,07897	2004	*Geophagus proximus*	3	32	0,16	Sampaio da Silva et al. 2009
Capitua	Middle Tapajós	-4,33219	-56,07897	2004	*Schizodon vittatus*	1	2	0,36	Sampaio da Silva et al. 2009
Capitua	Middle Tapajós	-4,33219	-56,07897	2004	*Anostomoides laticeps*	3	2	0,15	Sampaio da Silva et al. 2009
Capitua	Middle Tapajós	-4,33219	-56,07897	2004	*Anostomoides laticeps*	3	16	0,27	Sampaio da Silva et al. 2009
Capitua	Middle Tapajós	-4,33219	-56,07897	2004	*Caenotropus labyrinthicus*	2	52	0,26	Sampaio da Silva et al. 2009
Capitua	Middle Tapajós	-4,33219	-56,07897	2004	*Caenotropus labyrinthicus*	2	4	0,33	Sampaio da Silva et al. 2009
Capitua	Middle Tapajós	-4,33219	-56,07897	2004	*Hemiodus unimaculatus*	2	2	0,46	Sampaio da Silva et al. 2009
Capitua	Middle Tapajós	-4,33219	-56,07897	2004	*Hemiodus unimaculatus*	2	17	0,85	Sampaio da Silva et al. 2009
Capitua	Middle Tapajós	-4,33219	-56,07897	2004	*Acestrorhynchus falcirostris*	4	27	0,89	Sampaio da Silva et al. 2009
Capitua	Middle Tapajós	-4,33219	-56,07897	2004	*Plagioscion squamosissimus*	4	2	0,55	Sampaio da Silva et al. 2009
Capitua	Middle Tapajós	-4,33219	-56,07897	2004	*Plagioscion squamosissimus*	4	6	0,16	Sampaio da Silva et al. 2009
São Chico	Jamanxim	-6,42528	-56,06083	2003	*Potamotrygon motoro*	4	3	0,63	Castilhos et al. 2015
Creporizinho	Upper Tapajós	-6,50472	-56,585	2003	*Ageneiosus brevifilis*	4	1	0,27	Castilhos et al. 2015
São Chico	Jamanxim	-6,42528	-56,06083	2003	*Hemigrammus unilineatus*	3	4	0,43	Castilhos et al. 2015
São Chico	Jamanxim	-6,42528	-56,06083	2003	*Serrasalmus rhombeus*	4	2	0,93	Castilhos et al. 2015
Creporizinho	Upper Tapajós	-6,50472	-56,585	2003	*Serrasalmus rhombeus*	4	16	0,34	Castilhos et al. 2015
São Chico	Jamanxim	-6,42528	-56,06083	2003	*Phractocephalus hemioliopterus*	2	1	0,28	Castilhos et al. 2015
São Chico	Jamanxim	-6,42528	-56,06083	2003	*Pseudoplatystoma fasciatum*	4	1	1,20	Castilhos et al. 2015
Creporizinho	Upper Tapajós	-6,50472	-56,585	2003	*Hoplias malabaricus*	4	10	0,80	Castilhos et al. 2015
São Chico	Jamanxim	-6,42528	-56,06083	2003	*Hypostomus sp.*	1	2	0,03	Castilhos et al. 2015
Creporizinho	Upper Tapajós	-6,50472	-56,585	2003	*Hypostomus sp.*	1	13	0,05	Castilhos et al. 2015
São Chico	Jamanxim	-6,42528	-56,06083	2003	*Prochilodus nigricans*	1	8	0,17	Castilhos et al. 2015
Creporizinho	Upper Tapajós	-6,50472	-56,585	2003	*Prochilodus nigricans*	1	6	0,12	Castilhos et al. 2015
São Chico	Jamanxim	-6,42528	-56,06083	2003	*Myleus sp.*	1	1	0,09	Castilhos et al. 2015
São Chico	Jamanxim	-6,42528	-56,06083	2003	*Anostomoides laticeps*	3	1	0,14	Castilhos et al. 2015
Creporizinho	Upper Tapajós	-6,50472	-56,585	2003	*Anostomoides laticeps*	3	15	0,08	Castilhos et al. 2015
São Chico	Jamanxim	-6,42528	-56,06083	2003	*Sternopygus macrurus*	3	5	0,30	Castilhos et al. 2015
Creporizinho	Upper Tapajós	-6,50472	-56,585	2003	*Cyphocharax sp.*	1	50	0,23	Castilhos et al. 2015

Creporizinho	Upper Tapajós	-6,50472	-56,585	2003	*Cetopsis candiru*	2	4	0,73	Castilhos et al. 2015
São Chico	Jamanxim	-6,42528	-56,06083	2003	*Pimelodus blochii*	2	2	0,92	Castilhos et al. 2015
Creporizinho	Upper Tapajós	-6,50472	-56,585	2003	*Pimelodus blochii*	2	2	0,98	Castilhos et al. 2015
Rio Teles Pires	Teles Pires	-11,58	-55,65139	2016	*Brycon falcatus*	2	45	0,05	Matos et al. 2018
Itaituba	Middle Tapajós	-4,27583	-55,98389	2013	*Zungaro zungaro*	4	5	3,30	Martín-Doimeadios et al. 2014
Itaituba	Middle Tapajós	-4,27583	-55,98389	2013	*Brachyplatystoma filamentosum*	4	6	1,30	Martín-Doimeadios et al. 2014
Itaituba	Middle Tapajós	-4,27583	-55,98389	2013	*Plagioscion squamosissimus*	4	5	0,62	Martín-Doimeadios et al. 2014
Itaituba	Middle Tapajós	-4,27583	-55,98389	2013	*Pellona sp.*	4	4	2,30	Martín-Doimeadios et al. 2014
Itaituba	Middle Tapajós	-4,27583	-55,98389	2013	*Cichla sp.*	4	5	1,80	Martín-Doimeadios et al. 2014
Itaituba	Middle Tapajós	-4,27583	-55,98389	2013	*Pseudoplatystoma sp.*	4	5	0,66	Martín-Doimeadios et al. 2014
Itaituba	Middle Tapajós	-4,27583	-55,98389	2013	*Leporinus sp.*	2	5	0,13	Martín-Doimeadios et al. 2014
Itaituba	Middle Tapajós	-4,27583	-55,98389	2013	*Satanoperca sp.*	3	5	0,18	Martín-Doimeadios et al. 2014
Itaituba	Middle Tapajós	-4,27583	-55,98389	2013	*Colossoma macropomum*	1	5	0,14	Martín-Doimeadios et al. 2014
Itaituba	Middle Tapajós	-4,27583	-55,98389	2013	*Mylossoma sp.*	1	5	0,03	Martín-Doimeadios et al. 2014
Sai Cinza	Upper Tapajós	-6,44737	-57,97235	1999		4	71	0,25	Santos et al. 2003
Sai Cinza	Upper Tapajós	-6,44737	-57,97235	1999			90	0,11	Santos et al. 2003
Jacareacanga	Upper Tapajós	-6,2224	-57,75578	1999		4	770	0,36	Santos et al. 2003
Jacareacanga	Upper Tapajós	-6,2224	-57,75578	1999			307	0,12	Santos et al. 2003
São Luís do Tapajós	Middle Tapajós	-4,46011	-56,24997	1999		4	390	0,35	Santos et al. 2003
São Luís do Tapajós	Middle Tapajós	-4,46011	-56,24997	1999			138	0,09	Santos et al. 2003
Itaituba	Middle Tapajós	-4,26473	-55,99195	1999		4	431	0,40	Santos et al. 2003
Itaituba	Middle Tapajós	-4,26473	-55,99195	1999			80	0,06	Santos et al. 2003
Barreiras	Middle Tapajós	-4,09103	-55,68927	1999		4	657	0,45	Santos et al. 2003
Barreiras	Middle Tapajós	-4,09103	-55,68927	1999			466	0,09	Santos et al. 2003
Brasília Legal	Middle Tapajós	-3,81915	-55,60321	1999		4	728	0,44	Santos et al. 2003
Brasília Legal	Middle Tapajós	-3,81915	-55,60321	1999			135	0,07	Santos et al. 2003
Aveiro	Middle Tapajós	-3,60556	-55,33119	1999		4	415	0,26	Santos et al. 2003
Aveiro	Middle Tapajós	-3,60556	-55,33119	1999			163	0,04	Santos et al. 2003

Santarém	Lower Tapajós	-2,44104	-54,71352	1999		4	1167	0,16	Santos et al. 2003
Santarém	Lower Tapajós	-2,44104	-54,71352	1999			436	0,09	Santos et al. 2003
Alta Floresta	Teles Pires	-9,86818	-56,08353	1995	*Prochilodus nigricans*	1		0,17	Hacon et al. 2000
Alta Floresta	Teles Pires	-9,86818	-56,08353	1995	*Zungaro zungaro*	4		1,00	Hacon et al. 2000
Alta Floresta	Teles Pires	-9,86818	-56,08353	1995	*Brycon sp.*	2		0,01	Hacon et al. 2000
Alta Floresta	Teles Pires	-9,86818	-56,08353	1995	*Pseudoplatystoma fasciatum*	4		0,60	Hacon et al. 2000
Rio Kabitutu	Upper Tapajós	-6,58081	-57,65193	2003		4	6	0,20	Dorea et al. 2005
Rio Kabitutu	Upper Tapajós	-6,58081	-57,65193	2003			4	0,03	Dorea et al. 2005
Rio Cururu	Upper Tapajós	-7,79387	-57,27647	2003		4	7	0,20	Dorea et al. 2005
Rio Cururu	Upper Tapajós	-7,79387	-57,27647	2003			62	0,05	Dorea et al. 2005
Rio Cururuzinho	Teles Pires	-8,89503	-57,24593	2003		4	101	0,78	Dorea et al. 2005
Rio Cururuzinho	Teles Pires	-8,89503	-57,24593	2003			11	0,04	Dorea et al. 2005
Rio Tapajós	Upper Tapajós	-7,21318	-58,13335	2003		4	20	0,32	Dorea et al. 2005
Rio Tapajós	Upper Tapajós	-7,21318	-58,13335	2003			11	0,10	Dorea et al. 2005
Rio Teles Pires	Teles Pires	-7,40735	-58,05556	2003		4	56	0,67	Dorea et al. 2005
Rio Teles Pires	Teles Pires	-7,40735	-58,05556	2003			13	0,07	Dorea et al. 2005
Rio Tropas	Upper Tapajós	-6,14437	-57,62103	2003		4	49	0,26	Dorea et al. 2005
Rio Tropas	Upper Tapajós	-6,14437	-57,62103	2003		1	25	0,03	Dorea et al. 2005
Buburé	Middle Tapajós	-4,61041	-56,32814	2014	*Semaprochilodus insignis*	1	15	0,08	Lino et al. 2018
Buburé	Middle Tapajós	-4,61041	-56,32814	2014	*Schizodon vittatus*	1	4	0,09	Lino et al. 2018
Buburé	Middle Tapajós	-4,61041	-56,32814	2014	*Myloplus schomburgkii*	1	4	0,04	Lino et al. 2018
Buburé	Middle Tapajós	-4,61041	-56,32814	2014	*Leporinus friderici*	2	3	0,12	Lino et al. 2018
Buburé	Middle Tapajós	-4,61041	-56,32814	2014	*Rhytiodus sp.*	1	3	0,18	Lino et al. 2018
Buburé	Middle Tapajós	-4,61041	-56,32814	2014	*Triportheus auritus*	2	5	0,05	Lino et al. 2018
Buburé	Middle Tapajós	-4,61041	-56,32814	2014	*Geophagus proximus*	3	3	0,12	Lino et al. 2018
Buburé	Middle Tapajós	-4,61041	-56,32814	2014	*Hemisorubim platyrhynchos*	4	3	0,49	Lino et al. 2018
Buburé	Middle Tapajós	-4,61041	-56,32814	2014	*Serrasalmus calmoni*	4	3	0,40	Lino et al. 2018

Buburé	Middle Tapajós	-4,61041	-56,32814	2014	*Plagioscion squamosissimus*	4	3	1,51	Lino et al. 2018
Buburé	Middle Tapajós	-4,61041	-56,32814	2014	*Cichla monoculus*	4	5	0,74	Lino et al. 2018
Itaituba	Middle Tapajós	-4,26473	-55,99195	2014	*Psectrogaster rutiloides*	1	4	0,20	Lino et al. 2018
Itaituba	Middle Tapajós	-4,26473	-55,99195	2014	*Leiarius marmoratus*	4	6	0,11	Lino et al. 2018
Itaituba	Middle Tapajós	-4,26473	-55,99195	2014	*Hypophthalmus marginatus*	3	6	0,26	Lino et al. 2018
Itaituba	Middle Tapajós	-4,26473	-55,99195	2014	*Plagioscion squamosissimus*	4	7	0,91	Lino et al. 2018
Itaituba	Middle Tapajós	-4,26473	-55,99195	2014	*Cichla monoculus*	4	7	0,61	Lino et al. 2018
Itaituba	Middle Tapajós	-4,26473	-55,99195	2014	*Cichla pleiozona*	4	3	0,63	Lino et al. 2018
Alter do Chão	Lower Tapajós	-2,50683	-54,95046	2014	*Brycon amazonicus*	2	4	0,37	Lino et al. 2018
Alter do Chão	Lower Tapajós	-2,50683	-54,95046	2014	*Prochilodus nigricans*	1	4	0,19	Lino et al. 2018
Alter do Chão	Lower Tapajós	-2,50683	-54,95046	2014	*Hypophthalmus edentatus*	3	4	0,34	Lino et al. 2018
Santarém	Lower Tapajós	-2,44104	-54,71352	2014	*Brycon amazonicus*	2	7	0,29	Lino et al. 2018
Santarém	Lower Tapajós	-2,44104	-54,71352	2014	*Piaractus brachypomus*	1	4	0,03	Lino et al. 2018
Santarém	Lower Tapajós	-2,44104	-54,71352	2014	*Colossoma macropomum*	1	4	0,04	Lino et al. 2018
Santarém	Lower Tapajós	-2,44104	-54,71352	2014	*Semaprochilodus taeniurus*	1	6	0,13	Lino et al. 2018
Santarém	Lower Tapajós	-2,44104	-54,71352	2014	*Schizodon fasciatus*	1	8	0,11	Lino et al. 2018
Santarém	Lower Tapajós	-2,44104	-54,71352	2014	*Pseudoplatystoma fasciatum*	4	4	0,78	Lino et al. 2018
Itaituba	Middle Tapajós	-4,26473	-55,99195	2011	*Cichla pinima*	4	26	0,74	Azevedo et al. 2019
Comunidade Sai Cinza	Upper Tapajós	-6,235	-57,77528	1995	*Pinirampus pirinampu*	4	3	0,42	Brabo et al. 1999
Comunidade Sai Cinza	Upper Tapajós	-6,235	-57,77528	1995	*Pseudoplatystoma fasciatum*	4	1	0,39	Brabo et al. 1999
Comunidade Sai Cinza	Upper Tapajós	-6,235	-57,77528	1995	*Hoplias malabaricus*	4	7	0,32	Brabo et al. 1999
Comunidade Sai Cinza	Upper Tapajós	-6,235	-57,77528	1995	*Cichla ocellaris*	4	17	0,27	Brabo et al. 1999
Comunidade Sai Cinza	Upper Tapajós	-6,235	-57,77528	1995	*Serrasalmus sp.*	4	3	0,22	Brabo et al. 1999
Comunidade Sai Cinza	Upper Tapajós	-6,235	-57,77528	1995	*Osteoglossum bicirrhosum*	3	1	0,17	Brabo et al. 1999
Comunidade Sai Cinza	Upper Tapajós	-6,235	-57,77528	1995	*Satanoperca sp.*	3	18	0,12	Brabo et al. 1999
Comunidade Sai Cinza	Upper Tapajós	-6,235	-57,77528	1995	*Semaprochilodus brama*	1	22	0,11	Brabo et al. 1999
Comunidade Sai Cinza	Upper Tapajós	-6,235	-57,77528	1995	*Leporinus sp.*	2	2	0,11	Brabo et al. 1999

Comunidade Sai Cinza	Upper Tapajós	-6,235	-57,77528	1995	*Pimelodus ornatus*	2	1	0,10	Brabo et al. 1999
Comunidade Sai Cinza	Upper Tapajós	-6,235	-57,77528	1995	*Leiarius marmoratus*	4	3	0,09	Brabo et al. 1999
Comunidade Sai Cinza	Upper Tapajós	-6,235	-57,77528	1995	*Mylossoma sp.*	1	2	0,04	Brabo et al. 1999
Santarém	Lower Tapajós	-2,41972	-54,70444	1998	*Pellona sp.*	4	9	0,42	Castilhos et al. 1998
Itaituba	Middle Tapajós	-4,25639	-55,90917	1998	*Hydrolycus scomberoides*	4	5	0,69	Castilhos et al. 1998
Santarém	Lower Tapajós	-2,41972	-54,70444	1998	*Zungaro zungaro*	4	28	0,29	Castilhos et al. 1998
Santarém	Lower Tapajós	-2,41972	-54,70444	1998	*Brachyplatystoma filamentosum*	4	10	0,46	Castilhos et al. 1998
Itaituba	Middle Tapajós	-4,25639	-55,90917	1998	*Crenicichla sp.*	4	3	0,47	Castilhos et al. 1998
Itaituba	Middle Tapajós	-4,25639	-55,90917	1998	*Pimelodus blochii*	2	5	0,28	Castilhos et al. 1998
Santarém	Lower Tapajós	-2,41972	-54,70444	1998	*Plagioscion sp.*	4	29	0,22	Castilhos et al. 1998
Itaituba	Middle Tapajós	-4,25639	-55,90917	1998	*Plagioscion sp.*	4	33	0,43	Castilhos et al. 1998
Santarém	Lower Tapajós	-2,41972	-54,70444	1998	*Brachyplatystoma vaillantii*	4	11	0,06	Castilhos et al. 1998
Itaituba	Middle Tapajós	-4,25639	-55,90917	1998	*Brachyplatystoma vaillantii*	4	7	0,41	Castilhos et al. 1998
Santarém	Lower Tapajós	-2,41972	-54,70444	1998	*Serrasalmus sp.*	4	15	0,18	Castilhos et al. 1998
Itaituba	Middle Tapajós	-4,25639	-55,90917	1998	*Serrasalmus sp.*	4	6	0,10	Castilhos et al. 1998
Itaituba	Middle Tapajós	-4,25639	-55,90917	1998	*Rhaphiodon vulpinus*	4	4	0,62	Castilhos et al. 1998
Santarém	Lower Tapajós	-2,41972	-54,70444	1998	*Pseudoplatystoma fasciatum*	4	19	0,30	Castilhos et al. 1998
Itaituba	Middle Tapajós	-4,25639	-55,90917	1998	*Pseudoplatystoma fasciatum*	4	2	0,46	Castilhos et al. 1998
Santarém	Lower Tapajós	-2,41972	-54,70444	1998	*Hoplias sp.*	4	10	0,10	Castilhos et al. 1998
Santarém	Lower Tapajós	-2,41972	-54,70444	1998	*Cichla sp.*	4	28	0,12	Castilhos et al. 1998
Itaituba	Middle Tapajós	-4,25639	-55,90917	1998	*Cichla sp.*	4	33	0,42	Castilhos et al. 1998
Santarém	Lower Tapajós	-2,41972	-54,70444	1998	*Astronotus ocellatus*	2	17	0,04	Castilhos et al. 1998
Santarém	Lower Tapajós	-2,41972	-54,70444	1998	*Geophagus surinamensis*	3	11	0,02	Castilhos et al. 1998
Itaituba	Middle Tapajós	-4,25639	-55,90917	1998	*Geophagus surinamensis*	3	9	0,10	Castilhos et al. 1998
Santarém	Lower Tapajós	-2,41972	-54,70444	1998	*Laemolyta sp.*	1	26	0,05	Castilhos et al. 1998
Itaituba	Middle Tapajós	-4,25639	-55,90917	1998	*Laemolyta sp.*	1	39	0,06	Castilhos et al. 1998
Santarém	Lower Tapajós	-2,41972	-54,70444	1998	*Prochilodus nigricans*	1	20	0,02	Castilhos et al. 1998

Santarém	Lower Tapajós	-2,41972	-54,70444	1998	*Semaprochilodus brama*	1	26	0,04	Castilhos et al. 1998
Itaituba	Middle Tapajós	-4,25639	-55,90917	1998	*Semaprochilodus brama*	1	34	0,09	Castilhos et al. 1998
Santarém	Lower Tapajós	-2,41972	-54,70444	1998	*Hypophthalmus marginatus*	3	12	0,15	Castilhos et al. 1998
Itaituba	Middle Tapajós	-4,25639	-55,90917	1998	*Brycon sp.*	2	3	0,05	Castilhos et al. 1998
Santarém	Lower Tapajós	-2,41972	-54,70444	1998	*Myleus sp.*	1	32	0,01	Castilhos et al. 1998
Itaituba	Middle Tapajós	-4,25639	-55,90917	1998	*Myleus sp.*	1	50	0,04	Castilhos et al. 1998
Itaituba	Middle Tapajós	-4,25639	-55,90917	1998	*Colossoma macropomum*	1	5	0,08	Castilhos et al. 1998
Santarém	Lower Tapajós	-2,44104	-54,71352	2010	*Plagioscion squamosissimus*	4	5	1,43	Bourdineaud et al. 2015
Santarém	Lower Tapajós	-2,44104	-54,71352	2010	*Schizodon fasciatus*	1	5	0,49	Bourdineaud et al. 2015
Santarém	Lower Tapajós	-2,44104	-54,71352	2010	*Mylossoma duriventre*	1	5	0,48	Bourdineaud et al. 2015
Santarém	Lower Tapajós	-2,44104	-54,71352	2010	*Pellona castelnaeana*	4	5	1,66	Bourdineaud et al. 2015
Itaúba-Colíder	Teles Pires	-10,80374	-55,45544	2016	*Hydrolycus armatus*	4	6	0,23	Matos et al. 2021
Itaúba-Colíder	Teles Pires	-10,80374	-55,45544	2016	*Boulengerella cuvieri*	4	7	0,20	Matos et al. 2021
Itaúba-Colíder	Teles Pires	-10,80374	-55,45544	2016	*Serrasalmus rhombeus*	4	8	0,30	Matos et al. 2021
Sawré Muybu (Itaituba/Trairão)	Upper Tapajós	-5,49277	-57,05063	2019	*Serrasalmus rhombeus*	4	6	0,71	Vasconcellos et al. 2021
Sawré Muybu (Itaituba/Trairão)	Upper Tapajós	-5,49277	-57,05063	2019	*Pseudoplatystoma fasciatum*	4	6	0,24	Vasconcellos et al. 2021
Sawré Muybu (Itaituba/Trairão)	Upper Tapajós	-5,49277	-57,05063	2019	*Pinirampus pirinampu*	4	8	0,49	Vasconcellos et al. 2021
Sawré Muybu (Itaituba/Trairão)	Upper Tapajós	-5,49277	-57,05063	2019	*Cichla ocellaris*	4	6	0,33	Vasconcellos et al. 2021
Sawré Muybu (Itaituba/Trairão)	Upper Tapajós	-5,49277	-57,05063	2019	*Rhaphiodon vulpinus*	4	2	0,66	Vasconcellos et al. 2021
Sawré Muybu (Itaituba/Trairão)	Upper Tapajós	-5,49277	-57,05063	2019	*Ageneiosus inermis*	4	1	0,60	Vasconcellos et al. 2021
Sawré Muybu (Itaituba/Trairão)	Upper Tapajós	-5,49277	-57,05063	2019	*Pachyurus junki*	4	1	0,14	Vasconcellos et al. 2021
Sawré Muybu (Itaituba/Trairão)	Upper Tapajós	-5,49277	-57,05063	2019	*Geophagus proximus*	3	10	0,07	Vasconcellos et al. 2021
Sawré Muybu (Itaituba/Trairão)	Upper Tapajós	-5,49277	-57,05063	2019	*Pimelodus blochii*	2	7	0,20	Vasconcellos et al. 2021
Sawré Muybu (Itaituba/Trairão)	Upper Tapajós	-5,49277	-57,05063	2019	*Leporinus fasciatus*	2	5	0,09	Vasconcellos et al. 2021
Sawré Muybu (Itaituba/Trairão)	Upper Tapajós	-5,49277	-57,05063	2019	*Caenotropus labyrinthicus*	2	6	0,28	Vasconcellos et al. 2021
Sawré Muybu (Itaituba/Trairão)	Upper Tapajós	-5,49277	-57,05063	2019	*Hemiodus unimaculatus*	2	1	0,02	Vasconcellos et al. 2021
Sawré Muybu (Itaituba/Trairão)	Upper Tapajós	-5,49277	-57,05063	2019	*Schizodon vittatus*	1	4	0,03	Vasconcellos et al. 2021

Sawré Muybu (Itaituba/Trairão)	Upper Tapajós	-5,49277	-57,05063	2019	*Myloplus rubripinnis*	1	7	0,02	Vasconcellos et al. 2021
Sawré Muybu (Itaituba/Trairão)	Upper Tapajós	-5,49277	-57,05063	2019	*Semaprochilodus insignis*	1	6	0,11	Vasconcellos et al. 2021
Sawré Muybu (Itaituba/Trairão)	Upper Tapajós	-5,49277	-57,05063	2019	*Prochilodus nigricans*	1	6	0,07	Vasconcellos et al. 2021
Sawré Muybu (Itaituba/Trairão)	Upper Tapajós	-5,49277	-57,05063	2019	*Curimata sp.*	1	6	0,09	Vasconcellos et al. 2021
Lago Maicá (Santarém)	Lower Tapajós	-2,41972	-54,70444	1992	*Cichla sp.*	4	33	0,12	Vera et al. 2007
Rio Tapajós (Itaituba/Jacareacanga)	Middle Tapajós	-4,25639	-55,90917	1992	*Cichla sp.*	4	28	0,42	Vera et al. 2007
Lago Maicá (Santarém)	Lower Tapajós	-2,41972	-54,70444	2001	*Cichla sp.*	4	26	0,24	Vera et al. 2007
Rio Tapajós (Itaituba/Jacareacanga)	Middle Tapajós	-4,25639	-55,90917	2001	*Cichla sp.*	4	52	0,73	Vera et al. 2007
Rio Tapajós (Itaituba)	Middle Tapajós	-4,27556	-55,98219	2013	*Cichla sp.*	4	10	0,95	Oliveira et al. 2015
Rio Tapajós (Itaituba)	Middle Tapajós	-4,27556	-55,98219	2013	*Plagioscion squamosissimus*	4	10	0,53	Oliveira et al. 2015
Rio Tapajós (Itaituba)	Middle Tapajós	-4,27556	-55,98219	2013	*Leporinus sp.*	2	10	0,10	Oliveira et al. 2015
Rio Tapajós (Itaituba)	Middle Tapajós	-4,27556	-55,98219	2013	*Semaprochilodus sp.*	1	10	0,06	Oliveira et al. 2015
Paranaíta	Teles Pires	-9,66645	-56,47349	2007	*Acestrorhynchus sp.*	4	1	0,90	Farias 2007
Paranaíta	Teles Pires	-9,66645	-56,47349	2007	*Satanoperca sp.*	3	3	0,09	Farias 2007
Paranaíta	Teles Pires	-9,66645	-56,47349	2007	*Zungaro zungaro*	4	1	1,50	Farias 2007
Paranaíta	Teles Pires	-9,66645	-56,47349	2007	*Piaractus mesopotamicus*	1	1	0,02	Farias 2007
Paranaíta	Teles Pires	-9,66645	-56,47349	2007	*Pseudoplatystoma fasciatum*	4	1	0,63	Farias 2007
Paranaíta	Teles Pires	-9,66645	-56,47349	2007	*Brachyplatystoma filamentosum*	4	1	3,50	Farias 2007
Paranaíta	Teles Pires	-9,66645	-56,47349	2007	*Serrasalmus sp.*	4	11	0,37	Farias 2007
Paranaíta	Teles Pires	-9,66645	-56,47349	2007	*Colossoma macropomum*	1	16	0,04	Farias 2007
Paranaíta	Teles Pires	-9,66645	-56,47349	2007	*Hoplias malabaricus*	4	12	0,44	Farias 2007
Paranaíta	Teles Pires	-9,66645	-56,47349	2007	*Hoplias gr. lacerdae*	4	2	0,83	Farias 2007
Paranaíta	Teles Pires	-9,66645	-56,47349	2007	*Cichla ocellaris*	4	9	0,33	Farias 2007
Alta Floresta	Teles Pires	-9,86818	-56,08353	2007	*Piaractus mesopotamicus*	1	3	0,02	Farias 2007
Alta Floresta	Teles Pires	-9,86818	-56,08353	2007	*Prochilodus lineatus*	1	1	0,04	Farias 2007
Alta Floresta	Teles Pires	-9,86818	-56,08353	2007	*Leporinus sp.*	2	4	0,04	Farias 2007
Alta Floresta	Teles Pires	-9,86818	-56,08353	2007	*Pseudoplatystoma corruscans*	4	1	0,31	Farias 2007

Alta Floresta	Teles Pires	-9,86818	-56,08353	2007	*Colossoma macropomum*	1	35	0,02	Farias 2007
Alta Floresta	Teles Pires	-9,86818	-56,08353	2007	*Hoplias malabaricus*	4	2	0,18	Farias 2007
Alta Floresta	Teles Pires	-9,86818	-56,08353	2007	*Cichla ocellaris*	4	15	0,32	Farias 2007
Santarém	Lower Tapajós	-2,44104	-54,71352	1998	*Zungaro zungaro*	4	10	0,25	Lima et al. 2000
Santarém	Lower Tapajós	-2,44104	-54,71352	1998	*Hydrolycus sp.*	4	10	0,13	Lima et al. 2000
Santarém	Lower Tapajós	-2,44104	-54,71352	1998	*Plagioscion squamosissimus*	4	10	0,21	Lima et al. 2000
Santarém	Lower Tapajós	-2,44104	-54,71352	1998	*Pygocentrus nattereri*	4	5	0,28	Lima et al. 2000
Santarém	Lower Tapajós	-2,44104	-54,71352	1998	*Pellona sp.*	4	14	0,21	Lima et al. 2000
Santarém	Lower Tapajós	-2,44104	-54,71352	1998	*Pseudoplatystoma sp.*	4	10	0,20	Lima et al. 2000
Santarém	Lower Tapajós	-2,44104	-54,71352	1998	*Cichla sp.*	4	10	0,31	Lima et al. 2000
Santarém	Lower Tapajós	-2,44104	-54,71352	1998	*Mylossoma sp.*	1	10	0,03	Lima et al. 2000
Santarém	Lower Tapajós	-2,44104	-54,71352	1998	*Piaractus brachypomus*	1	10	0,01	Lima et al. 2000
Santarém	Lower Tapajós	-2,44104	-54,71352	1998	*Colossoma macropomum*	1	10	0,06	Lima et al. 2000
Santarém	Lower Tapajós	-2,44104	-54,71352	1998	*Leporinus sp.*	2	10	0,07	Lima et al. 2000
Itaituba	Middle Tapajós	-4,26473	-55,99195	1998	*Zungaro zungaro*	4	10	0,82	dos Santos et al. 2000
Itaituba	Middle Tapajós	-4,26473	-55,99195	1998	*Brachyplatystoma filamentosum*	4	10	0,42	dos Santos et al. 2000
Itaituba	Middle Tapajós	-4,26473	-55,99195	1998	*Pseudoplatystoma sp.*	4	10	0,84	dos Santos et al. 2000
Itaituba	Middle Tapajós	-4,26473	-55,99195	1998	*Plagioscion squamosissimus*	4	11	0,65	dos Santos et al. 2000
Itaituba	Middle Tapajós	-4,26473	-55,99195	1998	*Cichla sp.*	4	10	0,38	dos Santos et al. 2000
Itaituba	Middle Tapajós	-4,26473	-55,99195	1998	*Pellona sp.*	4	10	0,70	dos Santos et al. 2000
Itaituba	Middle Tapajós	-4,26473	-55,99195	1998	*Leporinus sp.*	2	13	0,07	dos Santos et al. 2000
Itaituba	Middle Tapajós	-4,26473	-55,99195	1998	*Prochilodus nigricans*	1	11	0,12	dos Santos et al. 2000
Itaituba	Middle Tapajós	-4,26473	-55,99195	1998	*Mylossoma sp.*	1	10	0,05	dos Santos et al. 2000
Itaituba	Middle Tapajós	-4,26473	-55,99195	1998	*Colossoma macropomum*	1	10	0,08	dos Santos et al. 2000
Santarém	Lower Tapajós	-2,44104	-54,71352	1996		1	6	0,01	Uryu et al. 2001
Rato River	Upper Tapajós	-5,42012	-56,90839	1996		1	2	0,05	Uryu et al. 2001
Santarém	Lower Tapajós	-2,44104	-54,71352	1996	*Semaprochilodus sp.*	1	1	0,03	Uryu et al. 2001

Brasília Legal	Middle Tapajós	-3,81915	- 55,60321	1996	*Semaprochilodus sp.*	1	2	0,03	Uryu et al. 2001
Santarém	Lower Tapajós	-2,44104	- 54,71352	1996		1	10	0,01	Uryu et al. 2001
Brasília Legal	Middle Tapajós	-3,81915	- 55,60321	1996		1	19	0,11	Uryu et al. 2001
São Luís do Tapajós	Middle Tapajós	-4,46011	- 56,24997	1996		1	2	0,06	Uryu et al. 2001
Santarém	Lower Tapajós	-2,44104	- 54,71352	1996	*Colossoma macropomum*	1	4	0,03	Uryu et al. 2001
Brasília Legal	Middle Tapajós	-3,81915	- 55,60321	1996	*Colossoma macropomum*	1	2	0,05	Uryu et al. 2001
Brasília Legal	Middle Tapajós	-3,81915	- 55,60321	1996	*Psectrogaster sp.*	1	4	0,07	Uryu et al. 2001
Brasília Legal	Middle Tapajós	-3,81915	- 55,60321	1996	*Geophagus sp.*	3	7	0,93	Uryu et al. 2001
Santarém	Lower Tapajós	-2,44104	- 54,71352	1996		1	30	0,03	Uryu et al. 2001
Brasília Legal	Middle Tapajós	-3,81915	- 55,60321	1996		1	8	0,06	Uryu et al. 2001
Itaituba	Middle Tapajós	-4,26473	- 55,99195	1996		1	1	0,08	Uryu et al. 2001
São Luís do Tapajós	Middle Tapajós	-4,46011	- 56,24997	1996		1	22	0,08	Uryu et al. 2001
Santarém	Lower Tapajós	-2,44104	- 54,71352	1996	*Prochilodus nigricans*	1	10	0,04	Uryu et al. 2001
Brasília Legal	Middle Tapajós	-3,81915	- 55,60321	1996	*Leporinus sp.*	2	2	0,11	Uryu et al. 2001
Jacareacanga	Upper Tapajós	-6,2224	- 57,75578	1996	*Anostomoides sp.*	3	1	0,12	Uryu et al. 2001
São Luís do Tapajós	Middle Tapajós	-4,46011	- 56,24997	1996	*Pachypops sp.*	2	2	0,15	Uryu et al. 2001
Brasília Legal	Middle Tapajós	-3,81915	- 55,60321	1996	*Brachyplatystoma vaillantii*	4	1	0,22	Uryu et al. 2001
Jacareacanga	Upper Tapajós	-6,2224	- 57,75578	1996	*Pseudoplatystoma sp.*	4	2	0,51	Uryu et al. 2001
Teles Pires River	Teles Pires	-8,33327	- 57,67678	1996	*Pseudoplatystoma sp.*	4	2	0,22	Uryu et al. 2001
Santarém	Lower Tapajós	-2,44104	- 54,71352	1996	*Arapaima gigas*	4	4	0,11	Uryu et al. 2001
Brasília Legal	Middle Tapajós	-3,81915	- 55,60321	1996	*Arapaima gigas*	4	1	0,32	Uryu et al. 2001
Itaituba	Middle Tapajós	-4,26473	- 55,99195	1996	*Arapaima gigas*	4	1	0,79	Uryu et al. 2001
Santarém	Lower Tapajós	-2,44104	- 54,71352	1996	*Hypophthalmus sp.*	3	5	0,13	Uryu et al. 2001
Itaituba	Middle Tapajós	-4,26473	- 55,99195	1996	*Hypophthalmus sp.*	3	1	0,62	Uryu et al. 2001
Santarém	Lower Tapajós	-2,44104	- 54,71352	1996	*Salminus sp.*	4	1	0,13	Uryu et al. 2001
Teles Pires River	Teles Pires	-8,33327	- 57,67678	1996	*Salminus sp.*	4	1	0,47	Uryu et al. 2001
Brasília Legal	Middle Tapajós	-3,81915	- 55,60321	1996	*Osteoglossum bicirrhosum*	3	1	0,51	Uryu et al. 2001

São Luís do Tapajós	Middle Tapajós	-4,46011	-56,24997	1996	*Osteoglossum bicirrhosum*	3	5	0,33	Uryu et al. 2001
Rato River	Upper Tapajós	-5,42012	-56,90839	1996	*Osteoglossum bicirrhosum*	3	2	0,61	Uryu et al. 2001
Jacareacanga	Upper Tapajós	-6,2224	-57,75578	1996	*Osteoglossum bicirrhosum*	3	1	0,40	Uryu et al. 2001
Santarém	Lower Tapajós	-2,44104	-54,71352	1996	*Zungaro zungaro*	4	1	0,09	Uryu et al. 2001
Itaituba	Middle Tapajós	-4,26473	-55,99195	1996	*Zungaro zungaro*	4	2	0,59	Uryu et al. 2001
Santarém	Lower Tapajós	-2,44104	-54,71352	1996	*Hoplias malabaricus*	4	6	0,13	Uryu et al. 2001
São Luís do Tapajós	Middle Tapajós	-4,46011	-56,24997	1996	*Hoplias malabaricus*	4	1	0,55	Uryu et al. 2001
Rato River	Upper Tapajós	-5,42012	-56,90839	1996	*Hoplias malabaricus*	4	1	1,11	Uryu et al. 2001
Jacareacanga	Upper Tapajós	-6,2224	-57,75578	1996	*Hoplias malabaricus*	4	2	0,72	Uryu et al. 2001
Teles Pires River	Teles Pires	-8,33327	-57,67678	1996	*Hoplias malabaricus*	4	2	0,82	Uryu et al. 2001
Santarém	Lower Tapajós	-2,44104	-54,71352	1996	*Zungaro zungaro*	4	1	0,23	Uryu et al. 2001
Teles Pires River	Teles Pires	-8,33327	-57,67678	1996	*Zungaro zungaro*	4	6	0,60	Uryu et al. 2001
Santarém	Lower Tapajós	-2,44104	-54,71352	1996	*Cichla sp.*	4	34	0,18	Uryu et al. 2001
Brasília Legal	Middle Tapajós	-3,81915	-55,60321	1996	*Cichla sp.*	4	9	0,79	Uryu et al. 2001
Itaituba	Middle Tapajós	-4,26473	-55,99195	1996	*Cichla sp.*	4	21	0,92	Uryu et al. 2001
Jacareacanga	Upper Tapajós	-6,2224	-57,75578	1996	*Cichla sp.*	4	20	0,32	Uryu et al. 2001
Santarém	Lower Tapajós	-2,44104	-54,71352	1996	*Serrasalmus sp.*	4	8	0,26	Uryu et al. 2001
Brasília Legal	Middle Tapajós	-3,81915	-55,60321	1996	*Serrasalmus sp.*	4	2	0,29	Uryu et al. 2001
São Luís do Tapajós	Middle Tapajós	-4,46011	-56,24997	1996	*Serrasalmus sp.*	4	2	0,85	Uryu et al. 2001
Rato River	Upper Tapajós	-5,42012	-56,90839	1996	*Serrasalmus sp.*	4	4	0,54	Uryu et al. 2001
Jacareacanga	Upper Tapajós	-6,2224	-57,75578	1996	*Serrasalmus sp.*	4	2	0,35	Uryu et al. 2001
Teles Pires River	Teles Pires	-8,33327	-57,67678	1996	*Serrasalmus sp.*	4	1	0,26	Uryu et al. 2001
Santarém	Lower Tapajós	-2,44104	-54,71352	1996	*Plagioscion sp.*	4	69	0,20	Uryu et al. 2001
Brasília Legal	Middle Tapajós	-3,81915	-55,60321	1996	*Plagioscion sp.*	4	7	0,54	Uryu et al. 2001
Itaituba	Middle Tapajós	-4,26473	-55,99195	1996	*Plagioscion sp.*	4	21	0,66	Uryu et al. 2001
São Luís do Tapajós	Middle Tapajós	-4,46011	-56,24997	1996	*Plagioscion sp.*	4	10	0,65	Uryu et al. 2001
Jacareacanga	Upper Tapajós	-6,2224	-57,75578	1996	*Plagioscion sp.*	4	2	0,54	Uryu et al. 2001

Teles Pires River	Teles Pires	-8,33327	-57,67678	1996	*Plagioscion sp.*	4	2	1,31	Uryu et al. 2001
Santarém	Lower Tapajós	-2,44104	-54,71352	1996		4	8	0,19	Uryu et al. 2001
Brasília Legal	Middle Tapajós	-3,81915	-55,60321	1996		4	6	0,60	Uryu et al. 2001
Jacareacanga	Upper Tapajós	-6,2224	-57,75578	1996		4	4	0,87	Uryu et al. 2001
Santarém	Lower Tapajós	-2,44104	-54,71352	1996	*Pellona sp.*	4	41	0,39	Uryu et al. 2001
Brasília Legal	Middle Tapajós	-3,81915	-55,60321	1996	*Pellona sp.*	4	2	0,51	Uryu et al. 2001
Itaituba	Middle Tapajós	-4,26473	-55,99195	1996	*Pellona sp.*	4	10	0,66	Uryu et al. 2001
Itaituba	Middle Tapajós	-4,26473	-55,99195	1996	*Brachyplatystoma platynema*	4	1	0,26	Uryu et al. 2001
São Luís do Tapajós	Middle Tapajós	-4,46011	-56,24997	1996	*Brachyplatystoma platynema*	4	2	1,05	Uryu et al. 2001
Jacareacanga	Upper Tapajós	-6,2224	-57,75578	1996	*Brachyplatystoma platynema*	4	2	1,06	Uryu et al. 2001
São Luís do Tapajós	Middle Tapajós	-4,46011	-56,24997	1996		4	8	0,76	Uryu et al. 2001
Rato River	Upper Tapajós	-5,42012	-56,90839	1996		4	2	1,38	Uryu et al. 2001
Teles Pires River	Teles Pires	-8,33327	-57,67678	1996		4	1	1,65	Uryu et al. 2001
Santarém	Lower Tapajós	-2,44104	-54,71352	1996	*Brachyplatystoma filamentosum*	4	2	0,43	Uryu et al. 2001
Itaituba	Middle Tapajós	-4,26473	-55,99195	1996	*Brachyplatystoma filamentosum*	4	13	1,03	Uryu et al. 2001
Teles Pires River	Teles Pires	-8,33327	-57,67678	1996	*Brachyplatystoma filamentosum*	4	4	2,35	Uryu et al. 2001
Santarém	Lower Tapajós	-2,44104	-54,71352	1996	*Ageneiosus sp.*	4	4	0,74	Uryu et al. 2001
São Luís do Tapajós	Middle Tapajós	-4,46011	-56,24997	1996	*Ageneiosus sp.*	4	2	1,43	Uryu et al. 2001
Rato River	Upper Tapajós	-5,42012	-56,90839	1996	*Ageneiosus sp.*	4	3	0,49	Uryu et al. 2001
próximo à Ilha do Ariosto, rio Teles Pires, Alta Floresta	Teles Pires	-9,86818	-56,08353	2012	*Argonectes sp.*	1	3	0,05	Castilhos et al. 2012
próximo à Ilha do Ariosto, rio Teles Pires, Alta Floresta	Teles Pires	-9,86818	-56,08353	2012	*Boulengerella sp.*	4	9	0,21	Castilhos et al. 2012
próximo à Ilha do Ariosto, rio Teles Pires, Alta Floresta	Teles Pires	-9,86818	-56,08353	2012	*Hydrolycus sp.*	4	18	0,19	Castilhos et al. 2012
próximo à Ilha do Ariosto, rio Teles Pires, Alta Floresta	Teles Pires	-9,86818	-56,08353	2012	*Prochilodus sp.*	1	20	0,10	Castilhos et al. 2012
próximo à Ilha do Ariosto, rio Teles Pires, Alta Floresta	Teles Pires	-9,86818	-56,08353	2012	*Astyanax sp.*	2	17	0,13	Castilhos et al. 2012
próximo à Ilha do Ariosto, rio Teles Pires, Alta Floresta	Teles Pires	-9,86818	-56,08353	2012	*Brycon sp.*	2	7	0,09	Castilhos et al. 2012
próximo à Ilha do Ariosto, rio Teles Pires, Alta Floresta	Teles Pires	-9,86818	-56,08353	2012	*Piaractus sp.*	1	7	0,07	Castilhos et al. 2012
próximo à Ilha do Ariosto, rio Teles Pires, Alta Floresta	Teles Pires	-9,86818	-56,08353	2012	*Leporinus sp.*	2	6	0,09	Castilhos et al. 2012

próximo à Ilha do Ariosto, rio Teles Pires, Alta Floresta	Teles Pires	-9,86818	-56,08353	2012		4	4	0,24	Castilhos et al. 2012
próximo à Ilha do Ariosto, rio Teles Pires, Alta Floresta	Teles Pires	-9,86818	-56,08353	2012	*Triportheus sp.*	2	7	0,14	Castilhos et al. 2012
próximo à Ilha do Ariosto, rio Teles Pires, Alta Floresta	Teles Pires	-9,86818	-56,08353	2012	*Hoplias sp.*	4	8	0,09	Castilhos et al. 2012
Itaituba	Middle Tapajós	-4,27611	-55,98361	2019	*Cichla sp.*	4	24	0,39	Oliveira et al. 2022
Itaituba	Middle Tapajós	-4,27611	-55,98361	2019	*Cichla sp.*	4	26	0,62	Oliveira et al. 2022
Itaituba	Middle Tapajós	-4,27611	-55,98361	2019	*Plagioscion squamosissimus*	4	24	0,54	Oliveira et al. 2022
Itaituba	Middle Tapajós	-4,27611	-55,98361	2019	*Plagioscion squamosissimus*	4	24	0,54	Oliveira et al. 2022
Jacareacanga/Itaituba	Upper Tapajós	-6,2224	-57,75578	1996	*Hydrolycus scomberoides*	4	5	0,69	Bidonet et al. 1997
Jacareacanga/Itaituba	Upper Tapajós	-6,2224	-57,75578	1996	*Crenicichla sp.*	4	3	0,47	Bidonet et al. 1997
Jacareacanga/Itaituba	Upper Tapajós	-6,2224	-57,75578	1996	*Pimelodus blochii*	2	5	0,28	Bidonet et al. 1997
Jacareacanga/Itaituba	Upper Tapajós	-6,2224	-57,75578	1996	*Plagioscion sp.*	4	33	0,43	Bidonet et al. 1997
Jacareacanga/Itaituba	Upper Tapajós	-6,2224	-57,75578	1996	*Brachyplatystoma vaillantii*	4	7	0,41	Bidonet et al. 1997
Jacareacanga/Itaituba	Upper Tapajós	-6,2224	-57,75578	1996	*Serrasalmus sp.*	4	6	0,10	Bidonet et al. 1997
Jacareacanga/Itaituba	Upper Tapajós	-6,2224	-57,75578	1996	*Hoplias sp.*	4	4	0,62	Bidonet et al. 1997
Jacareacanga/Itaituba	Upper Tapajós	-6,2224	-57,75578	1996	*Pseudoplatystoma fasciatum*	4	2	0,46	Bidonet et al. 1997
Jacareacanga/Itaituba	Upper Tapajós	-6,2224	-57,75578	1996	*Cichla sp.*	4	33	0,42	Bidonet et al. 1997
Jacareacanga/Itaituba	Upper Tapajós	-6,2224	-57,75578	1996	*Geophagus surinamensis*	3	9	0,10	Bidonet et al. 1997
Jacareacanga/Itaituba	Upper Tapajós	-6,2224	-57,75578	1996	*Semaprochilodus brama*	1	34	0,09	Bidonet et al. 1997
Jacareacanga/Itaituba	Upper Tapajós	-6,2224	-57,75578	1996	*Laemolyta sp.*	1	39	0,06	Bidonet et al. 1997
Jacareacanga/Itaituba	Upper Tapajós	-6,2224	-57,75578	1996	*Brycon sp.*	2	3	0,05	Bidonet et al. 1997
Jacareacanga/Itaituba	Upper Tapajós	-6,2224	-57,75578	1996	*Myleus sp.*	1	50	0,04	Bidonet et al. 1997
Jacareacanga/Itaituba	Upper Tapajós	-6,2224	-57,75578	1996	*Colossoma macropomum*	1	5	0,08	Bidonet et al. 1997
Lago Bom Intento	Middle Tapajós	-3,97806	-55,58944	2001	*Curimata inornata*	1	3	0,07	Sampaio da Silva et al. 2006
Lago Bom Intento	Middle Tapajós	-3,97806	-55,58944	2001	*Geophagus proximus*	3	5	0,06	Sampaio da Silva et al. 2006
Lago Bom Intento	Middle Tapajós	-3,97806	-55,58944	2001	*Schizodon fasciatus*	1	2	0,03	Sampaio da Silva et al. 2006
Lago Bom Intento	Middle Tapajós	-3,97806	-55,58944	2001	*Hemiodus unimaculatus*	2	25	0,06	Sampaio da Silva et al. 2006
Lago Bom Intento	Middle Tapajós	-3,97806	-55,58944	2001	*Catoprion mento*	2	1	0,09	Sampaio da Silva et al. 2006

Lago Bom Intento	Middle Tapajós	-3,97806	- 55,58944	2001	*Serrasalmus eigenmanni*	4	2	0,14	Sampaio da Silva et al. 2006
Lago Bom Intento	Middle Tapajós	-3,97806	- 55,58944	2001	*Pygocentrus nattereri*	4	4	0,37	Sampaio da Silva et al. 2006
Lago Bom Intento	Middle Tapajós	-3,97806	- 55,58944	2001	*Hoplias malabaricus*	4	4	0,15	Sampaio da Silva et al. 2006
Lago Bom Intento	Middle Tapajós	-3,97806	- 55,58944	2001	*Cichla temensis*	4	2	0,13	Sampaio da Silva et al. 2006
Lago Bom Intento	Middle Tapajós	-3,97806	- 55,58944	2001	*Cichla monoculus*	4	2	0,16	Sampaio da Silva et al. 2006
Lago Bom Intento	Middle Tapajós	-3,97806	- 55,58944	2001	*Potamorhina altamazonica*	1	5	0,03	Sampaio da Silva et al. 2006
Lago Bom Intento	Middle Tapajós	-3,97806	- 55,58944	2001	*Curimata inornata*	1	35	0,03	Sampaio da Silva et al. 2006
Lago Bom Intento	Middle Tapajós	-3,97806	- 55,58944	2001	*Semaprochilodus insignis*	1	2	0,02	Sampaio da Silva et al. 2006
Lago Bom Intento	Middle Tapajós	-3,97806	- 55,58944	2001	*Hoplosternum littorale*	3	1	0,14	Sampaio da Silva et al. 2006
Lago Bom Intento	Middle Tapajós	-3,97806	- 55,58944	2001	*Pterygoplichthys pardalis*	1	7	0,07	Sampaio da Silva et al. 2006
Lago Bom Intento	Middle Tapajós	-3,97806	- 55,58944	2001	*Cichlasoma amazonarum*	2	1	0,02	Sampaio da Silva et al. 2006
Lago Bom Intento	Middle Tapajós	-3,97806	- 55,58944	2001	*Satanoperca acuticeps*	3	5	0,15	Sampaio da Silva et al. 2006
Lago Bom Intento	Middle Tapajós	-3,97806	- 55,58944	2001	*Symphysodon aequifasciatus*	2	2	0,18	Sampaio da Silva et al. 2006
Lago Bom Intento	Middle Tapajós	-3,97806	- 55,58944	2001	*Astronotus crassipinnis*	2	1	0,09	Sampaio da Silva et al. 2006
Lago Bom Intento	Middle Tapajós	-3,97806	- 55,58944	2001	*Schizodon vittatus*	1	15	0,05	Sampaio da Silva et al. 2006
Lago Bom Intento	Middle Tapajós	-3,97806	- 55,58944	2001	*Schizodon fasciatus*	1	6	0,07	Sampaio da Silva et al. 2006
Lago Bom Intento	Middle Tapajós	-3,97806	- 55,58944	2001	*Geophagus proximus*	3	7	0,05	Sampaio da Silva et al. 2006
Lago Bom Intento	Middle Tapajós	-3,97806	- 55,58944	2001	*Hemiodus unimaculatus*	2	12	0,05	Sampaio da Silva et al. 2006
Lago Bom Intento	Middle Tapajós	-3,97806	- 55,58944	2001	*Platydoras costatus*	3	1	0,08	Sampaio da Silva et al. 2006
Lago Bom Intento	Middle Tapajós	-3,97806	- 55,58944	2001	*Osteoglossum bicirrhosum*	3	3	0,54	Sampaio da Silva et al. 2006
Lago Bom Intento	Middle Tapajós	-3,97806	- 55,58944	2001	*Hoplias malabaricus*	4	8	0,42	Sampaio da Silva et al. 2006
Lago Bom Intento	Middle Tapajós	-3,97806	- 55,58944	2001	*Pygocentrus nattereri*	4	8	0,41	Sampaio da Silva et al. 2006
Lago Bom Intento	Middle Tapajós	-3,97806	- 55,58944	2001	*Serrasalmus rhombeus*	4	3	0,38	Sampaio da Silva et al. 2006
Lago Bom Intento	Middle Tapajós	-3,97806	- 55,58944	2001	*Serrasalmus eigenmanni*	4	4	0,27	Sampaio da Silva et al. 2006
Lago Bom Intento	Middle Tapajós	-3,97806	- 55,58944	2001	*Cichla monoculus*	4	5	0,46	Sampaio da Silva et al. 2006
Lago Bom Intento	Middle Tapajós	-3,97806	- 55,58944	2001	*Cichla sp.*	4	4	0,24	Sampaio da Silva et al. 2006
Lago Bom Intento	Middle Tapajós	-3,97806	- 55,58944	2001	*Rhaphiodon vulpinus*	4	2	0,49	Sampaio da Silva et al. 2006

Lago Bom Intento	Middle Tapajós	-3,97806	-55,58944	2001	*Pseudoplatystoma tigrinum*	4	1	0,38	Sampaio da Silva et al. 2006
Lago Bom Intento	Middle Tapajós	-3,97806	-55,58944	2001	*Plagioscion squamosissimus*	4	7	0,33	Sampaio da Silva et al. 2006
Lago Cupu	Middle Tapajós	-4,03528	-55,60083	2001	*Curimata inornata*	1	2	0,05	Sampaio da Silva et al. 2006
Lago Cupu	Middle Tapajós	-4,03528	-55,60083	2001	*Geophagus proximus*	3	4	0,07	Sampaio da Silva et al. 2006
Lago Cupu	Middle Tapajós	-4,03528	-55,60083	2001	*Schizodon fasciatus*	1	12	0,07	Sampaio da Silva et al. 2006
Lago Cupu	Middle Tapajós	-4,03528	-55,60083	2001	*Schizodon vittatus*	1	5	0,13	Sampaio da Silva et al. 2006
Lago Cupu	Middle Tapajós	-4,03528	-55,60083	2001	*Leporinus friderici*	2	2	0,05	Sampaio da Silva et al. 2006
Lago Cupu	Middle Tapajós	-4,03528	-55,60083	2001	*Leporinus fasciatus*	2	4	0,15	Sampaio da Silva et al. 2006
Lago Cupu	Middle Tapajós	-4,03528	-55,60083	2001	*Hemiodus unimaculatus*	2	17	0,09	Sampaio da Silva et al. 2006
Lago Cupu	Middle Tapajós	-4,03528	-55,60083	2001	*Serrasalmus eigenmanni*	4	2	0,34	Sampaio da Silva et al. 2006
Lago Cupu	Middle Tapajós	-4,03528	-55,60083	2001	*Pygocentrus nattereri*	4	2	0,07	Sampaio da Silva et al. 2006
Lago Cupu	Middle Tapajós	-4,03528	-55,60083	2001	*Hoplias malabaricus*	4	1	0,25	Sampaio da Silva et al. 2006
Lago Cupu	Middle Tapajós	-4,03528	-55,60083	2001	*Cichla temensis*	4	11	0,45	Sampaio da Silva et al. 2006
Lago Cupu	Middle Tapajós	-4,03528	-55,60083	2001	*Cichla monoculus*	4	4	0,63	Sampaio da Silva et al. 2006
Lago Cupu	Middle Tapajós	-4,03528	-55,60083	2001	*Osteoglossum bicirrhosum*	3	1	0,82	Sampaio da Silva et al. 2006
Lago Cupu	Middle Tapajós	-4,03528	-55,60083	2001	*Pellona castelnaeana*	4	5	0,45	Sampaio da Silva et al. 2006
Lago Cupu	Middle Tapajós	-4,03528	-55,60083	2001	*Acestrorhynchus sp.*	4	3	0,44	Sampaio da Silva et al. 2006
Lago Cupu	Middle Tapajós	-4,03528	-55,60083	2001	*Potamorhina altamazonica*	1	1	0,03	Sampaio da Silva et al. 2006
Lago Cupu	Middle Tapajós	-4,03528	-55,60083	2001	*Pterygoplichthys pardalis*	1	2	0,08	Sampaio da Silva et al. 2006
Lago Cupu	Middle Tapajós	-4,03528	-55,60083	2001	*Schizodon fasciatus*	1	8	0,16	Sampaio da Silva et al. 2006
Lago Cupu	Middle Tapajós	-4,03528	-55,60083	2001	*Schizodon vittatus*	1	2	0,24	Sampaio da Silva et al. 2006
Lago Cupu	Middle Tapajós	-4,03528	-55,60083	2001	*Leporinus affinis*	2	3	0,12	Sampaio da Silva et al. 2006
Lago Cupu	Middle Tapajós	-4,03528	-55,60083	2001	*Hypophthalmus marginatus*	3	2	0,17	Sampaio da Silva et al. 2006
Lago Cupu	Middle Tapajós	-4,03528	-55,60083	2001	*Hemiodus unimaculatus*	2	20	0,05	Sampaio da Silva et al. 2006
Lago Cupu	Middle Tapajós	-4,03528	-55,60083	2001	*Metynnis argenteus*	1	2	0,07	Sampaio da Silva et al. 2006
Lago Cupu	Middle Tapajós	-4,03528	-55,60083	2001	*Serrasalmus eigenmanni*	4	14	0,20	Sampaio da Silva et al. 2006
Lago Cupu	Middle Tapajós	-4,03528	-55,60083	2001	*Pygocentrus nattereri*	4	1	0,52	Sampaio da Silva et al. 2006

Lago Cupu	Middle Tapajós	-4,03528	-55,60083	2001	*Serrasalmus rhombeus*	4	14	0,12	Sampaio da Silva et al. 2006
Lago Cupu	Middle Tapajós	-4,03528	-55,60083	2001	*Cichla monoculus*	4	3	0,43	Sampaio da Silva et al. 2006
Lago Cupu	Middle Tapajós	-4,03528	-55,60083	2001	*Cichla temensis*	4	8	0,49	Sampaio da Silva et al. 2006
Lago Cupu	Middle Tapajós	-4,03528	-55,60083	2001	*Cichla sp.*	4	2	0,41	Sampaio da Silva et al. 2006
Lago Cupu	Middle Tapajós	-4,03528	-55,60083	2001	*Acestrorhynchus falcirostris*	4	2	0,76	Sampaio da Silva et al. 2006
Lago Cupu	Middle Tapajós	-4,03528	-55,60083	2001	*Osteoglossum bicirrhosum*	3	1	0,13	Sampaio da Silva et al. 2006
Lago Cupu	Middle Tapajós	-4,03528	-55,60083	2001	*Pellona castelnaeana*	4	3	0,63	Sampaio da Silva et al. 2006
Lago Cupu	Middle Tapajós	-4,03528	-55,60083	2001	*Pseudoplatystoma tigrinum*	4	1	0,56	Sampaio da Silva et al. 2006
Lago Cupu	Middle Tapajós	-4,03528	-55,60083	2001	*Plagioscion squamosissimus*	4	6	0,39	Sampaio da Silva et al. 2006
Lago Pereira	Middle Tapajós	-4,035	-55,60111	2001	*Semaprochilodus insignis*	1	4	0,05	Sampaio da Silva et al. 2006
Lago Pereira	Middle Tapajós	-4,035	-55,60111	2001	*Schizodon fasciatus*	1	54	0,05	Sampaio da Silva et al. 2006
Lago Pereira	Middle Tapajós	-4,035	-55,60111	2001	*Leporinus fasciatus*	2	7	0,13	Sampaio da Silva et al. 2006
Lago Pereira	Middle Tapajós	-4,035	-55,60111	2001	*Leporinus friderici*	2	8	0,04	Sampaio da Silva et al. 2006
Lago Pereira	Middle Tapajós	-4,035	-55,60111	2001	*Geophagus proximus*	3	10	0,07	Sampaio da Silva et al. 2006
Lago Pereira	Middle Tapajós	-4,035	-55,60111	2001	*Mylossoma aureum*	1	13	0,04	Sampaio da Silva et al. 2006
Lago Pereira	Middle Tapajós	-4,035	-55,60111	2001	*Mylossoma sp.*	1	15	0,05	Sampaio da Silva et al. 2006
Lago Pereira	Middle Tapajós	-4,035	-55,60111	2001	*Hemiodus unimaculatus*	2	26	0,06	Sampaio da Silva et al. 2006
Lago Pereira	Middle Tapajós	-4,035	-55,60111	2001	*Triportheus albus*	2	2	0,22	Sampaio da Silva et al. 2006
Lago Pereira	Middle Tapajós	-4,035	-55,60111	2001	*Crenicichla sp.*	4	3	0,23	Sampaio da Silva et al. 2006
Lago Pereira	Middle Tapajós	-4,035	-55,60111	2001	*Serrasalmus eigenmanni*	4	16	0,47	Sampaio da Silva et al. 2006
Lago Pereira	Middle Tapajós	-4,035	-55,60111	2001	*Pygocentrus nattereri*	4	10	0,32	Sampaio da Silva et al. 2006
Lago Pereira	Middle Tapajós	-4,035	-55,60111	2001	*Serrasalmus rhombeus*	4	11	0,14	Sampaio da Silva et al. 2006
Lago Pereira	Middle Tapajós	-4,035	-55,60111	2001	*Hoplias malabaricus*	4	4	0,38	Sampaio da Silva et al. 2006
Lago Pereira	Middle Tapajós	-4,035	-55,60111	2001	*Cichla monoculus*	4	6	0,42	Sampaio da Silva et al. 2006
Lago Pereira	Middle Tapajós	-4,035	-55,60111	2001	*Cichla temensis*	4	8	0,35	Sampaio da Silva et al. 2006
Lago Pereira	Middle Tapajós	-4,035	-55,60111	2001	*Rhaphiodon vulpinus*	4	9	0,50	Sampaio da Silva et al. 2006
Lago Pereira	Middle Tapajós	-4,035	-55,60111	2001	*Pellona castelnaeana*	4	11	0,52	Sampaio da Silva et al. 2006

Lago Pereira	Middle Tapajós	-4,035	-55,60111	2001	*Plagioscion squamosissimus*	4	12	0,46	Sampaio da Silva et al. 2006
Lago Pereira	Middle Tapajós	-4,035	-55,60111	2001	*Acestrorhynchus sp.*	4	4	0,41	Sampaio da Silva et al. 2006
Lago Pereira	Middle Tapajós	-4,035	-55,60111	2001	*Potamorhina altamazonica*	1	8	0,03	Sampaio da Silva et al. 2006
Lago Pereira	Middle Tapajós	-4,035	-55,60111	2001	*Curimata inornata*	1	26	0,03	Sampaio da Silva et al. 2006
Lago Pereira	Middle Tapajós	-4,035	-55,60111	2001	*Schizodon fasciatus*	1	4	0,09	Sampaio da Silva et al. 2006
Lago Pereira	Middle Tapajós	-4,035	-55,60111	2001	*Schizodon vittatus*	1	7	0,04	Sampaio da Silva et al. 2006
Lago Pereira	Middle Tapajós	-4,035	-55,60111	2001	*Rhytiodus argenteofuscus*	1	12	0,13	Sampaio da Silva et al. 2006
Lago Pereira	Middle Tapajós	-4,035	-55,60111	2001	*Leporinus friderici*	2	4	0,13	Sampaio da Silva et al. 2006
Lago Pereira	Middle Tapajós	-4,035	-55,60111	2001	*Leporinus affinis*	2	1	0,08	Sampaio da Silva et al. 2006
Lago Pereira	Middle Tapajós	-4,035	-55,60111	2001	*Anostomoides laticeps*	3	1	0,45	Sampaio da Silva et al. 2006
Lago Pereira	Middle Tapajós	-4,035	-55,60111	2001	*Geophagus proximus*	3	5	0,03	Sampaio da Silva et al. 2006
Lago Pereira	Middle Tapajós	-4,035	-55,60111	2001	*Satanoperca acuticeps*	3	3	0,04	Sampaio da Silva et al. 2006
Lago Pereira	Middle Tapajós	-4,035	-55,60111	2001	*Hemiodus unimaculatus*	2	11	0,04	Sampaio da Silva et al. 2006
Lago Pereira	Middle Tapajós	-4,035	-55,60111	2001	*Triportheus albus*	2	10	0,15	Sampaio da Silva et al. 2006
Lago Pereira	Middle Tapajós	-4,035	-55,60111	2001	*Ageneiosus brevifilis*	4	6	0,62	Sampaio da Silva et al. 2006
Lago Pereira	Middle Tapajós	-4,035	-55,60111	2001	*Ageneiosus sp.*	4	2	0,44	Sampaio da Silva et al. 2006
Lago Pereira	Middle Tapajós	-4,035	-55,60111	2001	*Oxydoras niger*	3	1	0,17	Sampaio da Silva et al. 2006
Lago Pereira	Middle Tapajós	-4,035	-55,60111	2001	*Platydoras costatus*	3	8	0,18	Sampaio da Silva et al. 2006
Lago Pereira	Middle Tapajós	-4,035	-55,60111	2001	*Serrasalmus eigenmanni*	4	4	0,43	Sampaio da Silva et al. 2006
Lago Pereira	Middle Tapajós	-4,035	-55,60111	2001	*Pygocentrus nattereri*	4	5	0,42	Sampaio da Silva et al. 2006
Lago Pereira	Middle Tapajós	-4,035	-55,60111	2001	*Serrasalmus rhombeus*	4	1	0,88	Sampaio da Silva et al. 2006
Lago Pereira	Middle Tapajós	-4,035	-55,60111	2001	*Hoplias malabaricus*	4	2	0,35	Sampaio da Silva et al. 2006
Lago Pereira	Middle Tapajós	-4,035	-55,60111	2001	*Cichla monoculus*	4	1	0,38	Sampaio da Silva et al. 2006
Lago Pereira	Middle Tapajós	-4,035	-55,60111	2001	*Cichla temensis*	4	1	0,12	Sampaio da Silva et al. 2006
Lago Pereira	Middle Tapajós	-4,035	-55,60111	2001	*Pseudoplatystoma tigrinum*	4	3	0,37	Sampaio da Silva et al. 2006
Lago Pereira	Middle Tapajós	-4,035	-55,60111	2001	*Rhaphiodon vulpinus*	4	4	0,60	Sampaio da Silva et al. 2006
Lago Pereira	Middle Tapajós	-4,035	-55,60111	2001	*Plagioscion squamosissimus*	4	51	0,37	Sampaio da Silva et al. 2006

Itaituba	Middle Tapajós	-4,26473	-55,99195	1992	*Cichla sp.*	4		0,73	Lacerda e Pfeiffer 1992
Itaituba	Middle Tapajós	-4,26473	-55,99195	1992		1		0,15	Lacerda e Pfeiffer 1992
Alta Floresta	Teles Pires	-9,86818	-56,08353	1995	*Brachyplatystoma rousseauxii*	4	1	0,60	Akagi et al. 1995
Alta Floresta	Teles Pires	-9,86818	-56,08353	1995	*Zungaro zungaro*	4	1	0,61	Akagi et al. 1995
Alta Floresta	Teles Pires	-9,86818	-56,08353	1995	*Zungaro zungaro*	4	1	0,75	Akagi et al. 1995
Alta Floresta	Teles Pires	-9,86818	-56,08353	1995	*Zungaro zungaro*	4	1	1,03	Akagi et al. 1995
Alta Floresta	Teles Pires	-9,86818	-56,08353	1995	*Zungaro zungaro*	4	1	0,39	Akagi et al. 1995
Alta Floresta	Teles Pires	-9,86818	-56,08353	1995	*Brachyplatystoma filamentosum*	4	1	3,82	Akagi et al. 1995
Alta Floresta	Teles Pires	-9,86818	-56,08353	1995	*Brachyplatystoma filamentosum*	4	1	2,85	Akagi et al. 1995
Alta Floresta	Teles Pires	-9,86818	-56,08353	1995		4	1	0,29	Akagi et al. 1995
Rato River	Upper Tapajós	-5,42012	-56,90839	1995	*Osteoglossum bicirrhosum*	3	1	0,28	Akagi et al. 1995
Rato River	Upper Tapajós	-5,42012	-56,90839	1995	*Ageneiosus sp.*	4	1	0,53	Akagi et al. 1995
Rato River	Upper Tapajós	-5,42012	-56,90839	1995	*Ageneiosus sp.*	4	1	0,56	Akagi et al. 1995
Rato River	Upper Tapajós	-5,42012	-56,90839	1995	*Hydrolycus sp.*	4	1	1,60	Akagi et al. 1995
Rato River	Upper Tapajós	-5,42012	-56,90839	1995	*Hoplias sp.*	4	1	0,95	Akagi et al. 1995
Brasília Legal	Middle Tapajós	-3,81915	-55,60321	1995	*Geophagus sp.*	3	1	0,17	Akagi et al. 1995
Brasília Legal	Middle Tapajós	-3,81915	-55,60321	1995	*Pellona sp.*	4	1	0,60	Akagi et al. 1995
Brasília Legal	Middle Tapajós	-3,81915	-55,60321	1995		1	1	0,10	Akagi et al. 1995
Brasília Legal	Middle Tapajós	-3,81915	-55,60321	1995	*Plagioscion sp.*	4	1	0,41	Akagi et al. 1995
Brasília Legal	Middle Tapajós	-3,81915	-55,60321	1995	*Cichla sp.*	4	1	1,16	Akagi et al. 1995
Itaituba	Middle Tapajós	-4,26473	-55,99195	1995	*Pellona sp.*	4	1	0,54	Akagi et al. 1995
Itaituba	Middle Tapajós	-4,26473	-55,99195	1995	*Brachyplatystoma filamentosum*	4	1	1,00	Akagi et al. 1995
Itaituba	Middle Tapajós	-4,26473	-55,99195	1995	*Plagioscion sp.*	4	1	0,56	Akagi et al. 1995
Santarém	Lower Tapajós	-2,44104	-54,71352	1995	*Pellona sp.*	4	1	0,39	Akagi et al. 1995
Santarém	Lower Tapajós	-2,44104	-54,71352	1995	*Arapaima sp.*	4	1	0,08	Akagi et al. 1995
Brasília Legal	Middle Tapajós	-3,98333	-55,5	1995		1	3	0,03	Lebel et al. 1997
Brasília Legal	Middle Tapajós	-3,98333	-55,5	1995	*Aphanotorulus emarginatus*	1	1	0,05	Lebel et al. 1997

Brasília Legal	Middle Tapajós	-3,98333	-55,5	1995	*Pellona castelnaeana*	4	2	0,61	Lebel et al. 1997
Brasília Legal	Middle Tapajós	-3,98333	-55,5	1995	*Pellona flavipinnis*	4	4	0,54	Lebel et al. 1997
Brasília Legal	Middle Tapajós	-3,98333	-55,5	1995	*Leporinus sp.*	2	4	0,11	Lebel et al. 1997
Brasília Legal	Middle Tapajós	-3,98333	-55,5	1995	*Leporinus sp.*	2	32	0,08	Lebel et al. 1997
Brasília Legal	Middle Tapajós	-3,98333	-55,5	1995	*Leporinus sp.*	2	7	0,33	Lebel et al. 1997
Brasília Legal	Middle Tapajós	-3,98333	-55,5	1995	*Pinirampus pirinampu*	4	1	0,28	Lebel et al. 1997
Brasília Legal	Middle Tapajós	-3,98333	-55,5	1995	*Geophagus sp.*	3	14	0,09	Lebel et al. 1997
Brasília Legal	Middle Tapajós	-3,98333	-55,5	1995	*Astronotus sp.*	2	1	0,17	Lebel et al. 1997
Brasília Legal	Middle Tapajós	-3,98333	-55,5	1995	*Pachypops sp.*	2	1	0,10	Lebel et al. 1997
Brasília Legal	Middle Tapajós	-3,98333	-55,5	1995	*Brachyplatystoma rousseauxii*	4	1	0,31	Lebel et al. 1997
Brasília Legal	Middle Tapajós	-3,98333	-55,5	1995	*Brachyplatystoma filamentosum*	4	4	0,50	Lebel et al. 1997
Brasília Legal	Middle Tapajós	-3,98333	-55,5	1995	*Crenicichla sp.*	4	1	0,27	Lebel et al. 1997
Brasília Legal	Middle Tapajós	-3,98333	-55,5	1995	*Hoplerythrinus unitaeniatus*	4	1	0,38	Lebel et al. 1997
Brasília Legal	Middle Tapajós	-3,98333	-55,5	1995	*Hypophthalmus sp.*	3	1	0,26	Lebel et al. 1997
Brasília Legal	Middle Tapajós	-3,98333	-55,5	1995	*Pimelodus sp.*	2	2	0,30	Lebel et al. 1997
Brasília Legal	Middle Tapajós	-3,98333	-55,5	1995	*Ageneiosus sp.*	4	1	0,40	Lebel et al. 1997
Brasília Legal	Middle Tapajós	-3,98333	-55,5	1995	*Characidium fasciatum*	3	2	0,18	Lebel et al. 1997
Brasília Legal	Middle Tapajós	-3,98333	-55,5	1995		1	5	0,04	Lebel et al. 1997
Brasília Legal	Middle Tapajós	-3,98333	-55,5	1995	*Mylossoma sp.*	1	1	0,02	Lebel et al. 1997
Brasília Legal	Middle Tapajós	-3,98333	-55,5	1995	*Metynnis sp.*	1	1	0,03	Lebel et al. 1997
Brasília Legal	Middle Tapajós	-3,98333	-55,5	1995	*Hydrolycus sp.*	4	1	0,52	Lebel et al. 1997
Brasília Legal	Middle Tapajós	-3,98333	-55,5	1995	*Plagioscion sp.*	4	36	0,53	Lebel et al. 1997
Brasília Legal	Middle Tapajós	-3,98333	-55,5	1995	*Plagioscion sp.*	4	1	0,06	Lebel et al. 1997
Brasília Legal	Middle Tapajós	-3,98333	-55,5	1995	*Plagioscion sp.*	4	2	0,10	Lebel et al. 1997
Brasília Legal	Middle Tapajós	-3,98333	-55,5	1995	*Calophysus macropterus*	4	2	0,76	Lebel et al. 1997
Brasília Legal	Middle Tapajós	-3,98333	-55,5	1995	*Arapaima sp.*	4	1	0,17	Lebel et al. 1997
Brasília Legal	Middle Tapajós	-3,98333	-55,5	1995	*Serrasalmus sp.*	4	11	0,53	Lebel et al. 1997

Brasília Legal	Middle Tapajós	-3,98333	-55,5	1995	*Pygocentrus nattereri*	4	5	0,55	Lebel et al. 1997
Brasília Legal	Middle Tapajós	-3,98333	-55,5	1995	*Serrasalmus sp.*	4	1	0,09	Lebel et al. 1997
Brasília Legal	Middle Tapajós	-3,98333	-55,5	1995	*Serrasalmus sp.*	4	1	0,13	Lebel et al. 1997
Brasília Legal	Middle Tapajós	-3,98333	-55,5	1995	*Serrasalmus rhombeus*	4	11	0,40	Lebel et al. 1997
Brasília Legal	Middle Tapajós	-3,98333	-55,5	1995	*Cynodon gibbus*	4	2	0,37	Lebel et al. 1997
Brasília Legal	Middle Tapajós	-3,98333	-55,5	1995	*Pellona sp.*	4	2	0,80	Lebel et al. 1997
Brasília Legal	Middle Tapajós	-3,98333	-55,5	1995	*Triportheus sp.*	2	6	0,13	Lebel et al. 1997
Brasília Legal	Middle Tapajós	-3,98333	-55,5	1995	*Pseudoplatystoma sp.*	4	1	0,36	Lebel et al. 1997
Brasília Legal	Middle Tapajós	-3,98333	-55,5	1995	*Colossoma macropomum*	1	1	0,07	Lebel et al. 1997
Brasília Legal	Middle Tapajós	-3,98333	-55,5	1995	*Hoplias sp.*	4	1	0,49	Lebel et al. 1997
Brasília Legal	Middle Tapajós	-3,98333	-55,5	1995	*Cichla sp.*	4	6	0,40	Lebel et al. 1997
Alta Floresta	Teles Pires	-9,86818	-56,08353	1993	*Brachyplatystoma sp.*	4	7	2,75	Hacon et al. 1997
Alta Floresta	Teles Pires	-9,86818	-56,08353	1993	*Zungaro zungaro*	4	12	1,00	Hacon et al. 1997
Alta Floresta	Teles Pires	-9,86818	-56,08353	1993	*Plagioscion sp.*	4	4	0,77	Hacon et al. 1997
Alta Floresta	Teles Pires	-9,86818	-56,08353	1993	*Pseudoplatystoma fasciatum*	4	21	0,60	Hacon et al. 1997
Alta Floresta	Teles Pires	-9,86818	-56,08353	1993	*Zungaro zungaro*	4	6	0,53	Hacon et al. 1997
Alta Floresta	Teles Pires	-9,86818	-56,08353	1993		4	4	0,38	Hacon et al. 1997
Alta Floresta	Teles Pires	-9,86818	-56,08353	1993	*Hoplias malabaricus*	4	8	0,36	Hacon et al. 1997
Alta Floresta	Teles Pires	-9,86818	-56,08353	1993	*Serrasalmus sp.*	4	12	0,29	Hacon et al. 1997
Alta Floresta	Teles Pires	-9,86818	-56,08353	1993	*Cichla ocellaris*	4	11	0,28	Hacon et al. 1997
Alta Floresta	Teles Pires	-9,86818	-56,08353	1993	*Mylossoma sp.*	1	13	0,08	Hacon et al. 1997
Alta Floresta	Teles Pires	-9,86818	-56,08353	1993	*Prochilodus nigricans*	1	5	0,08	Hacon et al. 1997
Matupá	Teles Pires	-10,17078	-54,92558	2001		4	125	0,58	Hacon et al. 2003
Matupá	Teles Pires	-10,17078	-54,92558	2001			129	0,03	Hacon et al. 2003
Nova Monte Verde/Nova Bandeirante	Juruena	-9,85009	-57,81416	2001			38	0,04	Hacon et al. 2003
Nova Monte Verde/Nova Bandeirante	Juruena	-9,85009	-57,81416	2001		4	32	0,48	Hacon et al. 2003
Creporizão, Rio Crepori	Upper Tapajós	-6,81988	-56,85126	2016	*Hoplias auri*	4	2	0,76	Silva 2017

Creporizão, Rio Crepori	Upper Tapajós	-6,82014	-56,848	2016	*Hoplias auri*	4	9	1,71	Silva 2017
Creporizão, Rio Crepori	Upper Tapajós	-6,83369	-56,84747	2016	*Hoplias auri*	4	4	0,50	Silva 2017
Santarém, Lago Juá	Lower Tapajós	-2,4381	-54,7854	2016	*Hoplias malabaricus*	4	10	0,46	Silva 2017
Vila Parauá, Lago Inajatuba	Lower Tapajós	-2,80225	-55,17817	2016	*Hoplias malabaricus*	4	8	0,54	This Study
Novo Mundo, Rio Peixoto	Teles Pires	-10,10917	-55,31867	2021	*Hoplias malabaricus*	4	3	0,39	This Study
Novo Mundo, Rio Peixoto	Teles Pires	-10,14908	-55,29308	2021	*Hoplias malabaricus*	4	2	0,66	This Study
Santarém, Igarapé-Açu	Lower Tapajós	-2,38892	-54,73344	2020	*Hoplias malabaricus*	4	5	0,28	This Study
Guarantã do Norte, Rio Peixoto	Teles Pires	-9,90831	-55,01517	2021	*Hoplias malabaricus*	4	2	0,25	This Study
Vila Pimental, Rio Tapajós	Middle Tapajós	-4,56818	-56,26006	2015	*Ancistrus hoplogenys*	1	4	0,05	This Study
Vila Pimental, Rio Tapajós	Middle Tapajós	-4,56818	-56,26006	2015	*Baryancistrus sp.*	1	4	0,04	This Study
Vila Pimental, Rio Tapajós	Middle Tapajós	-4,56818	-56,26006	2015	*Cichla sp.*	4	2	0,56	This Study
Vila Pimental, Rio Tapajós	Middle Tapajós	-4,56818	-56,26006	2015	*Geophagus altifrons*	3	6	0,22	This Study
Vila Pimental, Rio Tapajós	Middle Tapajós	-4,56818	-56,26006	2015	*Hypostomus soniae*	1	4	0,03	This Study
Vila Pimental, Rio Tapajós	Middle Tapajós	-4,56818	-56,26006	2015	*Hoplias curupira*	4	1	0,36	This Study
Vila Pimental, Rio Tapajós	Middle Tapajós	-4,56818	-56,26006	2015	*Hoplias malabaricus*	4	1	0,23	This Study
Vila Pimental, Rio Tapajós	Middle Tapajós	-4,56818	-56,26006	2015	*Leporacanthicus cf. joselimai*	1	4	0,13	This Study
Vila Pimental, Rio Tapajós	Middle Tapajós	-4,56818	-56,26006	2015	*Myloplus torquatus*	1	1	0,08	This Study
Vila Pimental, Rio Tapajós	Middle Tapajós	-4,56818	-56,26006	2015	*Panaqolus sp.*	1	4	0,19	This Study
Vila Pimental, Rio Tapajós	Middle Tapajós	-4,56818	-56,26006	2015	*Peckoltia vittata*	1	4	0,32	This Study
Itaituba	Middle Tapajós	-4,27583	-55,98389	2009	*Cichla ocellaris*	4	50	0,36	Vieira et al. 2011
Itaituba	Middle Tapajós	-4,27583	-55,98389	2009	*Colossoma macropomum*	1	48	0,26	Vieira et al. 2011
Santarém	Lower Tapajós	-2,44104	-54,71352	2000	*Cichla sp.*	4	8	0,16	Kehrig et al. 2008
Lago Curi (Brasília Legal)	Middle Tapajós	-3,81915	-55,60321	2000	*Cichla sp.*	4	10	0,50	Kehrig et al. 2008
Itaituba	Middle Tapajós	-4,26473	-55,99195	2000	*Cichla sp.*	4	8	0,89	Kehrig et al. 2008
Jacareacanga	Upper Tapajós	-6,2224	-57,75578	2000	*Cichla sp.*	4	16	0,62	Kehrig et al. 2008
Itaituba/Jacareacanga	Middle Tapajós	-4,25639	-55,90917	1992	*Cichla sp.*	4	33	0,41	Castilhos et al. 2003
Itaituba	Middle Tapajós	-4,25639	-55,90917	2001	*Cichla sp.*	4	55	0,71	Castilhos et al. 2003

Santarém	Lower Tapajós	-2,41972	- 54,70444	1992	*Cichla sp.*	4	28	0,12	Castilhos et al. 2003
Santarém	Lower Tapajós	-2,41972	- 54,70444	2001	*Cichla sp.*	4	53	0,24	Castilhos et al. 2003
Itaituba	Middle Tapajós	-4,25639	- 55,90917	1996	*Plagioscion sp.*	4	5	0,63	Kehrig e Malm 1999
Santarém	Lower Tapajós	-2,41972	- 54,70444	1996	*Plagioscion sp.*	4	6	0,11	Kehrig e Malm 1999
Itaituba	Middle Tapajós	-4,25639	- 55,90917	1996	*Cichla sp.*	4	5	0,99	Kehrig e Malm 1999
Santarém	Lower Tapajós	-2,41972	- 54,70444	1996	*Cichla sp.*	4	5	0,18	Kehrig e Malm 1999

Figure S3. Classification of areas to compare sub-basins in the Tapajós River basin according to the *Agência Nacional das Águas* (ANA) and our study. Maps were done with QGIS (v.3.30).

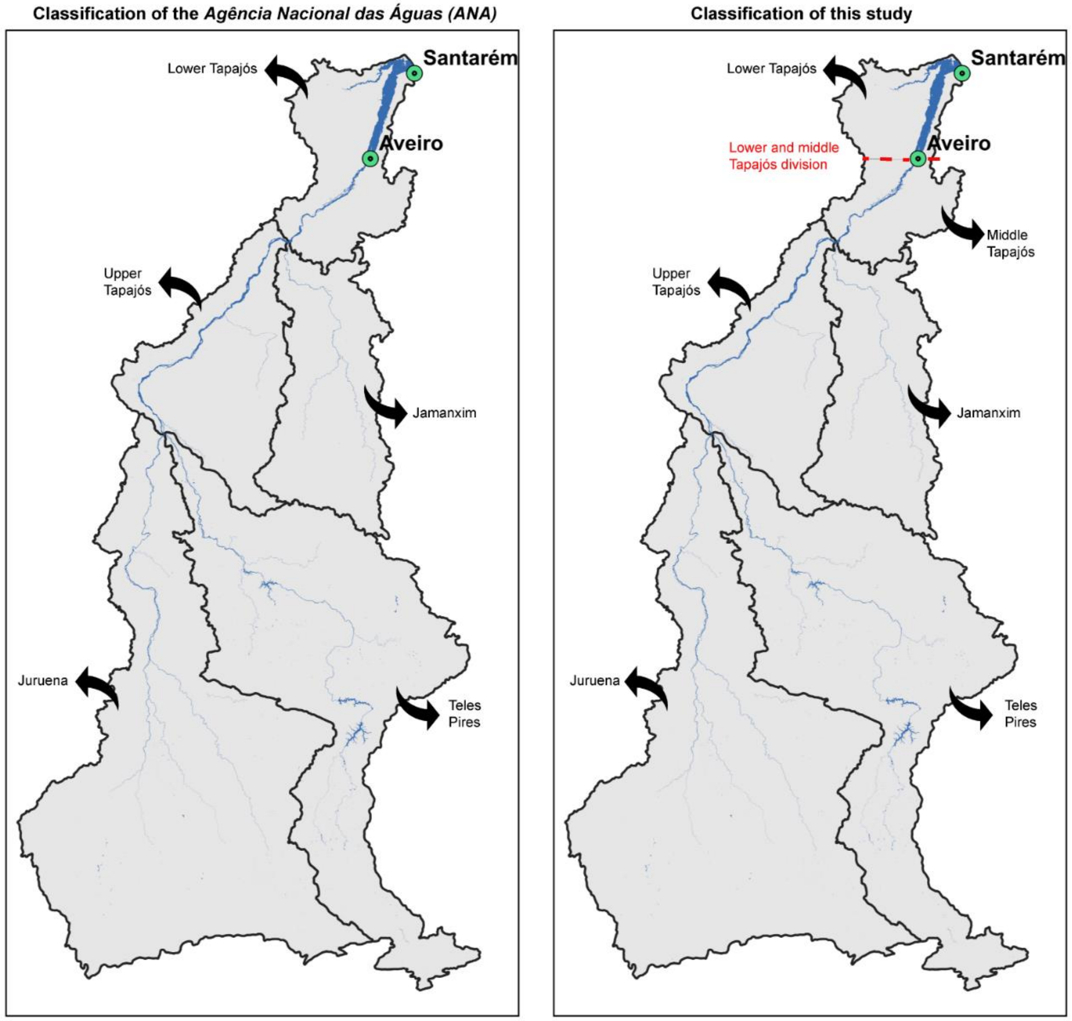

Table S4. Number of individuals analyzed per species in each paper evaluated, separated by sub-basin.

**Species**/Reference	**Lower Tapajós**	**Middle Tapajós**	**Upper Tapajós**	**Jamanxim**	**Juruena**	**Teles Pires**	**Total sample number**
**Acestrorhynchus falcirostris (Cuvier 1819)**		**155**					**155**
Sampaio da Silva et al. 2006		2					2
Sampaio da Silva et al. 2009		153					153
**Acestrorhynchus sp.**		**7**				**1**	**8**
Farias 2007						1	1
Sampaio da Silva et al. 2006		7					7
**Ageneiosus brevifilis Valenciennes 1840**		**6**	**1**				**7**
Castilhos et al. 2015			1				1
Sampaio da Silva et al. 2006		6					6
**Ageneiosus inermis (Linnaeus 1766)**			**1**				**1**
Vasconcellos et al. 2021			1				1
**Ageneiosus sp.**	**4**	**5**	**5**				**14**
Akagi et al. 1995			2				2
Lebel et al. 1997		1					1
Sampaio da Silva et al. 2006		2					2
Uryu et al. 2001	4	2	3				9
**Ancistrus hoplogenys (Günther 1864)**		**4**					**4**
This Study		4					4
**Anostomoides laticeps (Eigenmann 1912)**		**97**	**15**	**1**			**113**
Castilhos et al. 2015			15	1			16
Sampaio da Silva et al. 2006		1					1
Sampaio da Silva et al. 2009		96					96
**Anostomoides sp.**			**1**				**1**
Uryu et al. 2001			1				1
**Aphanotorulus emarginatus (Valenciennes 1840)**		**1**					**1**
Lebel et al. 1997		1					1
**Arapaima gigas (Schinz 1822)**	**4**	**2**					**6**
Uryu et al. 2001	4	2					6
**Arapaima sp.**	**1**	**1**					**2**
Akagi et al. 1995	1						1
Lebel et al. 1997		1					1
**Argonectes sp.**						**3**	**3**

Castilhos et al. 2012					3	3
**Astronotus crassipinnis (Heckel 1840)**		**3**				**3**
Faial et al. 2014		2				2
Sampaio da Silva et al. 2006		1				1
**Astronotus ocellatus (Agassiz 1831)**	**17**					**17**
Castilhos et al. 1998	17					17
**Astronotus sp.**		**1**				**1**
Lebel et al. 1997		1				1
**Astyanax sp.**					**17**	**17**
Castilhos et al. 2012					17	17
**Auchenipterus sp.**		**-**				**-**
Passos et al. 2008		-				-
**Baryancistrus sp.**		**4**				**4**
This Study		4				4
**Boulengerella cuvieri (Spix & Agassiz 1829)**					**7**	**7**
Matos et al. 2021					7	7
**Boulengerella sp.**					**9**	**9**
Castilhos et al. 2012					9	9
**Brachyplatystoma filamentosum (Lichtenstein 1819)**	**12**	**36**			**8**	**56**
Akagi et al. 1995					3	3
Castilhos et al. 1998	10					10
dos Santos et al. 2000		10				10
Faial et al. 2014		3				3
Farias 2007					1	1
Lebel et al. 1997		4				4
Martín-Doimeadios et al. 2014		6				6
Uryu et al. 2001	2	13			4	19
**Brachyplatystoma platynema Boulenger 1898**		**3**	**2**			**5**
Uryu et al. 2001		3	2			5
**Brachyplatystoma rousseauxii (Castelnau 1855)**		**1**			**1**	**2**
Akagi et al. 1995					1	1
Lebel et al. 1997		1				1
**Brachyplatystoma sp.**					**7**	**7**
Hacon et al. 1997					7	7
**Brachyplatystoma vaillantii (Valenciennes 1840)**	**11**	**8**	**7**			**26**

Bidone et al. 1997			7			7
Castilhos et al. 1998	11	7				18
Uryu et al. 2001		1				1
**Brycon amazonicus (Agassiz 1829)**	**11**					**11**
Lino et al. 2018	11					11
**Brycon falcatus Müller & Troschel 1844**					**45**	**45**
Matos et al. 2018					45	45
**Brycon sp.**		**3**	**3**		**7**	**13**
Bidone et al. 1997			3			3
Castilhos et al. 1998		3				3
Castilhos et al. 2012					7	7
Hacon et al. 2000					-	-
Passos et al. 2008		-				-
**Caenotropus labyrinthicus (Kner 1858)**		**192**	**6**			**198**
Sampaio da Silva et al. 2009		192				192
Vasconcellos et al. 2021			6			6
**Calophysus macropterus (Lichtenstein 1819)**		**16**				**16**
Faial et al. 2014		14				14
Lebel et al. 1997		2				2
**Catoprion mento (Cuvier 1819)**		**1**				**1**
Sampaio da Silva et al. 2006		1				1
**Cetopsis candiru Spix & Agassiz 1829**			**4**			**4**
Castilhos et al. 2015			4			4
**Characidium fasciatum Reinhardt 1867**		**2**				**2**
Lebel et al. 1997		2				2
**Cichla monoculus Spix & Agassiz 1831**		**33**				**33**
Lino et al. 2018		12				12
Sampaio da Silva et al. 2006		21				21
**Cichla ocellaris Bloch & Schneider 1801**		**50**	**23**		**35**	**108**
Brabo et al. 1999			17			17
Farias 2007					24	24
Hacon et al. 1997					11	11
Vasconcellos et al. 2021			6			6
Vieira et al. 2011		50				50
**Cichla pinima Kullander & Ferreira 2006**		**26**				**26**
Azevedo et al. 2019		26				26

**Cichla pleiozona Kullander & Ferreira 2006**		**3**				**3**
Lino et al. 2018		3				3
**Cichla sp.**	**225**	**357**	**69**			**651**
Akagi et al. 1995		1				1
Bidone et al. 1997			33			33
Castilhos et al. 1998	28	33				61
Castilhos et al. 2003	81	88				169
dos Santos et al. 2000		10				10
Faial et al. 2014		13				13
Kehrig e Malm 1999	5	5				10
Kehrig et al. 2008	8	18	16			42
Lacerda e Pfeiffer 1992		-				-
Lebel et al. 1997		6				6
Lima et al. 2000	10					10
Martín-Doimeadios et al. 2014		5				5
Oliveira et al. 2015		10				10
Oliveira et al. 2022		50				50
Passos et al. 2008		-				-
Sampaio da Silva et al. 2006		6				6
This Study		2				2
Uryu et al. 2001	34	30	20			84
Vera et al. 2007	59	80				139
**Cichla temensis Humboldt 1821**		**30**				**30**
Sampaio da Silva et al. 2006		30				30
**Cichlasoma amazonarum Kullander 1983**		**1**				**1**
Sampaio da Silva et al. 2006		1				1
**Colossoma macropomum (Cuvier 1816)**	**18**	**71**	**5**		**51**	**145**
Bidone et al. 1997			5			5
Castilhos et al. 1998		5				5
dos Santos et al. 2000		10				10
Farias 2007					51	51
Lebel et al. 1997		1				1
Lima et al. 2000	10					10
Lino et al. 2018	4					4
Martín-Doimeadios et al. 2014		5				5
Passos et al. 2008		-				-

Uryu et al. 2001	4	2			6
Vieira et al. 2011		48			48
**Crenicichla sp.**		**7**	**3**		**10**
Bidone et al. 1997			3		3
Castilhos et al. 1998		3			3
Lebel et al. 1997		1			1
Passos et al. 2008		-			-
Sampaio da Silva et al. 2006		3			3
**Curimata inornata Vari 1989**		**253**			**253**
Sampaio da Silva et al. 2006		66			66
Sampaio da Silva et al. 2009		187			187
**Curimata sp.**			**6**		**6**
Passos et al. 2008		-			-
Vasconcellos et al. 2021			6		6
**Cynodon gibbus (Spix & Agassiz 1829)**		**2**			**2**
Lebel et al. 1997		2			2
**Cyphocharax sp.**			**50**		**50**
Castilhos et al. 2015			50		50
**Geophagus altifrons Heckel 1840**		**6**			**6**
This Study		6			6
**Geophagus brasiliensis (Quoy & Gaimard 1824)**		**21**			**21**
Faial et al. 2014		21			21
**Geophagus proximus (Castelnau 1855)**		**191**	**10**		**201**
Lino et al. 2018		3			3
Sampaio da Silva et al. 2006		31			31
Sampaio da Silva et al. 2009		157			157
Vasconcellos et al. 2021			10		10
**Geophagus sp.**		**22**			**22**
Akagi et al. 1995		1			1
Lebel et al. 1997		14			14
Passos et al. 2008		-			-
Uryu et al. 2001		7			7
**Geophagus surinamensis (Bloch 1791)**	**11**	**9**	**9**		**29**
Bidone et al. 1997			9		9
Castilhos et al. 1998	11	9			20
**Hemigrammus unilineatus (Gill 1858)**				**4**	**4**

Castilhos et al. 2015				4		4
**Hemiodus sp.**		**3**				**3**
Faial et al. 2014		3				3
Passos et al. 2008		-				-
**Hemiodus unimaculatus (Bloch 1794)**		**438**	**1**			**439**
Sampaio da Silva et al. 2006		111				111
Sampaio da Silva et al. 2009		327				327
Vasconcellos et al. 2021			1			1
**Hemisorubim platyrhynchos (Valenciennes 1840)**		**3**				**3**
Lino et al. 2018		3				3
**Hoplerythrinus unitaeniatus (Spix & Agassiz 1829)**		**1**				**1**
Lebel et al. 1997		1				1
**Hoplias auri Guimarães, Rosso, González-Castro, Souza, Díaz de Astarloa & Rodrigues 2021**			**15**			**15**
Silva 2017			15			15
**Hoplias curupira Oyakawa & Mattox 2009**		**1**				**1**
This Study		1				1
**Hoplias lacerdae Miranda Ribeiro 1908**					**2**	**2**
Farias 2007					2	2
**Hoplias malabaricus (Bloch 1794)**	**29**	**66**	**20**		**31**	**146**
Brabo et al. 1999			7			7
Castilhos et al. 2015			10			10
Farias 2007					14	14
Hacon et al. 1997					8	8
Sampaio da Silva et al. 2006		19				19
Sampaio da Silva et al. 2009		45				45
Silva 2017	10					10
This Study	13	1			7	21
Uryu et al. 2001	6	1	3		2	12
**Hoplias sp.**	**10**	**1**	**5**		**8**	**24**
Akagi et al. 1995			1			1
Bidone et al. 1997			4			4
Castilhos et al. 1998	10					10
Castilhos et al. 2012					8	8
Lebel et al. 1997		1				1
Passos et al. 2008		-				-

**Hoplosternum littorale (Hancock 1828)**		**1**				**1**
Sampaio da Silva et al. 2006		1				1
**Hydrolycus armatus (Jardine 1841)**					**6**	**6**
Matos et al. 2021					6	6
**Hydrolycus scomberoides (Cuvier 1819)**		**5**	**5**			**10**
Bidone et al. 1997			5			5
Castilhos et al. 1998		5				5
**Hydrolycus sp.**	**10**	**1**	**1**		**18**	**30**
Akagi et al. 1995			1			1
Castilhos et al. 2012					18	18
Lebel et al. 1997		1				1
Lima et al. 2000	10					10
**Hypophthalmus edentatus Spix & Agassiz 1829**	**4**					**4**
Lino et al. 2018	4					4
**Hypophthalmus marginatus Valenciennes 1840**	**12**	**8**				**20**
Castilhos et al. 1998	12					12
Lino et al. 2018		6				6
Sampaio da Silva et al. 2006		2				2
**Hypophthalmus sp.**	**5**	**2**				**7**
Lebel et al. 1997		1				1
Passos et al. 2008		-				-
Uryu et al. 2001	5	1				6
**Hypostomus soniae Hollanda Carvalho & Weber 2005**		**4**				**4**
This Study		4				4
**Hypostomus sp.**			**13**	**2**		**15**
Castilhos et al. 2015			13	2		15
**Laemolyta sp.**	**26**	**39**	**39**			**104**
Bidone et al. 1997			39			39
Castilhos et al. 1998	26	39				65
**Leiarius marmoratus (Gill 1870)**		**6**	**3**			**9**
Brabo et al. 1999			3			3
Lino et al. 2018		6				6
**Leporacanthicus joselimai Isbrücker & Nijssen 1989**		**4**				**4**
This Study		4				4
**Leporinus affinis Günther 1864**		**4**				**4**
Sampaio da Silva et al. 2006		4				4

**Leporinus fasciatus (Bloch 1794)**		**86**	**5**			**91**
Sampaio da Silva et al. 2006		11				11
Sampaio da Silva et al. 2009		75				75
Vasconcellos et al. 2021			5			5
**Leporinus friderici (Bloch 1794)**		**17**				**17**
Lino et al. 2018		3				3
Sampaio da Silva et al. 2006		14				14
**Leporinus sp.**	**10**	**119**	**2**		**10**	**141**
Brabo et al. 1999			2			2
Castilhos et al. 2012					6	6
dos Santos et al. 2000		13				13
Faial et al. 2014		46				46
Farias 2007					4	4
Lebel et al. 1997		43				43
Lima et al. 2000	10					10
Martín-Doimeadios et al. 2014		5				5
Oliveira et al. 2015		10				10
Uryu et al. 2001		2				2
**Metynnis argenteus Ahl 1923**		**2**				**2**
Sampaio da Silva et al. 2006		2				2
**Metynnis sp.**		**1**				**1**
Lebel et al. 1997		1				1
**Myleus sp.**	**32**	**50**	**50**	**1**		**133**
Bidone et al. 1997			50			50
Castilhos et al. 1998	32	50				82
Castilhos et al. 2015				1		1
**Myloplus rubripinnis (Müller & Troschel 1844)**			**7**			**7**
Vasconcellos et al. 2021			7			7
**Myloplus schomburgkii (Jardine 1841)**		**4**				**4**
Lino et al. 2018		4				4
**Myloplus torquatus (Kner 1858)**		**1**				**1**
This Study		1				1
**Mylossoma aureum (Spix & Agassiz 1829)**		**13**				**13**
Sampaio da Silva et al. 2006		13				13
**Mylossoma duriventre (Cuvier 1818)**	**5**					**5**
Bourdineaud et al. 2015	5					5

**Mylossoma sp.**	**10**	**86**	**2**		**13**	**111**
Brabo et al. 1999			2			2
dos Santos et al. 2000		10				10
Faial et al. 2014		55				55
Hacon et al. 1997					13	13
Lebel et al. 1997		1				1
Lima et al. 2000	10					10
Martín-Doimeadios et al. 2014		5				5
Passos et al. 2008		-				-
Sampaio da Silva et al. 2006		15				15
**Osteoglossum bicirrhosum (Cuvier 1829)**		**11**	**5**			**16**
Akagi et al. 1995			1			1
Brabo et al. 1999			1			1
Passos et al. 2008		-				-
Sampaio da Silva et al. 2006		5				5
Uryu et al. 2001		6	3			9
**Oxydoras niger (Valenciennes 1821)**		**1**				**1**
Sampaio da Silva et al. 2006		1				1
**Oxydoras sp.**		**-**				**-**
Passos et al. 2008		-				-
**Pachypops sp.**		**3**				**3**
Lebel et al. 1997		1				1
Passos et al. 2008		-				-
Uryu et al. 2001		2				2
**Pachyurus junki Soares & Casatti 2000**			**1**			**1**
Vasconcellos et al. 2021			1			1
**Panaqolus sp.**		**4**				**4**
This Study		4				4
**Peckoltia vittata (Steindachner 1881)**		**4**				**4**
This Study		4				4
**Pellona castelnaeana Valenciennes 1847**	**5**	**21**				**26**
Bourdineaud et al. 2015	5					5
Lebel et al. 1997		2				2
Sampaio da Silva et al. 2006		19				19
**Pellona flavipinnis (Valenciennes 1837)**		**4**				**4**
Lebel et al. 1997		4				4

**Pellona sp.**	**65**	**30**				**95**
Akagi et al. 1995	1	2				3
Castilhos et al. 1998	9					9
dos Santos et al. 2000		10				10
Lebel et al. 1997		2				2
Lima et al. 2000	14					14
Martín-Doimeadios et al. 2014		4				4
Passos et al. 2008		-				-
Uryu et al. 2001	41	12				53
**Phractocephalus hemioliopterus (Bloch & Schneider 1801)**				**1**		**1**
Castilhos et al. 2015				1		1
**Piaractus brachypomus (Cuvier 1818)**	**14**					**14**
Lima et al. 2000	10					10
Lino et al. 2018	4					4
**Piaractus mesopotamicus (Holmberg 1887)**					**4**	**4**
Farias 2007					4	4
**Piaractus sp.**					**7**	**7**
Castilhos et al. 2012					7	7
**Pimelodus blochii Valenciennes 1840**		**5**	**14**	**2**		**21**
Bidone et al. 1997			5			5
Castilhos et al. 1998		5				5
Castilhos et al. 2015			2	2		4
Vasconcellos et al. 2021			7			7
**Pimelodus ornatus Kner 1858**			**1**			**1**
Brabo et al. 1999			1			1
**Pimelodus sp.**		**2**				**2**
Lebel et al. 1997		2				2
**Pinirampus pirinampu (Spix & Agassiz 1829)**		**1**	**11**			**12**
Brabo et al. 1999			3			3
Lebel et al. 1997		1				1
Vasconcellos et al. 2021			8			8
**Plagioscion sp.**	**104**	**117**	**35**		**6**	**262**
Akagi et al. 1995		2				2
Bidone et al. 1997			33			33
Castilhos et al. 1998	29	33				62
Hacon et al. 1997					4	4

Kehrig e Malm 1999	6	5				11
Lebel et al. 1997		39				39
Passos et al. 2008		-				-
Uryu et al. 2001	69	38	2		2	111
**Plagioscion squamosissimus (Heckel 1840)**	**15**	**440**				**455**
Bourdineaud et al. 2015	5					5
dos Santos et al. 2000		11				11
Faial et al. 2014		75				75
Lima et al. 2000	10					10
Lino et al. 2018		10				10
Martín-Doimeadios et al. 2014		5				5
Oliveira et al. 2015		10				10
Oliveira et al. 2022		48				48
Sampaio da Silva et al. 2006		76				76
Sampaio da Silva et al. 2009		205				205
**Platydoras costatus (Linnaeus 1758)**		**9**				**9**
Sampaio da Silva et al. 2006		9				9
**Potamorhina altamazonica (Cope 1878)**		**14**				**14**
Sampaio da Silva et al. 2006		14				14
**Potamotrygon motoro (Müller & Henle 1841)**				**3**		**3**
Castilhos et al. 2015				3		3
**Prochilodus lineatus (Valenciennes 1837)**					**1**	**1**
Farias 2007					1	1
**Prochilodus nigricans Spix & Agassiz 1829**	**34**	**11**	**12**	**8**	**5**	**70**
Castilhos et al. 1998	20					20
Castilhos et al. 2015			6	8		14
dos Santos et al. 2000		11				11
Hacon et al. 1997					5	5
Hacon et al. 2000					-	-
Lino et al. 2018	4					4
Uryu et al. 2001	10					10
Vasconcellos et al. 2021			6			6
**Prochilodus sp.**					**20**	**20**
Castilhos et al. 2012					20	20
Passos et al. 2008		-				-
**Psectrogaster rutiloides (Kner 1858)**		**4**				**4**

Lino et al. 2018		4				4
**Psectrogaster sp.**		**4**				**4**
Uryu et al. 2001		4				4
**Pseudoplatystoma corruscans (Spix & Agassiz 1829)**					**1**	**1**
Farias 2007					1	1
**Pseudoplatystoma fasciatum (Linnaeus 1766)**	**23**	**2**	**9**	**1**	**22**	**57**
Bidone et al. 1997			2			2
Brabo et al. 1999			1			1
Castilhos et al. 1998	19	2				21
Castilhos et al. 2015				1		1
Farias 2007					1	1
Hacon et al. 1997					21	21
Hacon et al. 2000					-	-
Lino et al. 2018	4					4
Vasconcellos et al. 2021			6			6
**Pseudoplatystoma sp.**	**10**	**16**	**2**		**2**	**30**
dos Santos et al. 2000		10				10
Lebel et al. 1997		1				1
Lima et al. 2000	10					10
Martín-Doimeadios et al. 2014		5				5
Passos et al. 2008		-				-
Uryu et al. 2001			2		2	4
**Pseudoplatystoma tigrinum (Valenciennes 1840)**		**5**				**5**
Sampaio da Silva et al. 2006		5				5
**Pterygoplichthys pardalis (Castelnau 1855)**		**9**				**9**
Passos et al. 2008		-				-
Sampaio da Silva et al. 2006		9				9
**Pygocentrus nattereri Kner 1858**	**5**	**35**				**40**
Lebel et al. 1997		5				5
Lima et al. 2000	5					5
Sampaio da Silva et al. 2006		30				30
**Rhaphiodon vulpinus Spix & Agassiz 1829**		**19**	**2**			**21**
Castilhos et al. 1998		4				4
Passos et al. 2008		-				-
Sampaio da Silva et al. 2006		15				15
Vasconcellos et al. 2021			2			2

**Rhytiodus argenteofuscus Kner 1858**		**12**				**12**
Sampaio da Silva et al. 2006		12				12
**Rhytiodus sp.**		**3**				**3**
Lino et al. 2018		3				3
**Salminus sp.**	**1**				**1**	**2**
Uryu et al. 2001	1				1	2
**Satanoperca acuticeps (Heckel 1840)**		**8**				**8**
Sampaio da Silva et al. 2006		8				8
**Satanoperca sp.**		**5**	**18**		**3**	**26**
Brabo et al. 1999			18			18
Farias 2007					3	3
Martín-Doimeadios et al. 2014		5				5
**Schizodon fasciatus Spix & Agassiz 1829**	**13**	**86**				**99**
Bourdineaud et al. 2015	5					5
Lino et al. 2018	8					8
Sampaio da Silva et al. 2006		86				86
**Schizodon sp.**		**-**				**-**
Passos et al. 2008		-				-
**Schizodon vittatus (Valenciennes 1850)**		**110**	**4**			**114**
Lino et al. 2018		4				4
Sampaio da Silva et al. 2006		29				29
Sampaio da Silva et al. 2009		77				77
Vasconcellos et al. 2021			4			4
**Semaprochilodus brama (Valenciennes 1850)**	**26**	**34**	**56**			**116**
Bidone et al. 1997			34			34
Brabo et al. 1999			22			22
Castilhos et al. 1998	26	34				60
**Semaprochilodus insignis (Jardine 1841)**		**21**	**6**			**27**
Lino et al. 2018		15				15
Sampaio da Silva et al. 2006		6				6
Vasconcellos et al. 2021			6			6
**Semaprochilodus sp.**	**1**	**12**				**13**
Oliveira et al. 2015		10				10
Passos et al. 2008		-				-
Uryu et al. 2001	1	2				3
**Semaprochilodus taeniurus (Valenciennes 1821)**	**6**					**6**

Lino et al. 2018	6					6
**Serrasalmus calmoni Steindachner 1908**		**3**				**3**
Lino et al. 2018		3				3
**Serrasalmus eigenmanni Norman 1929**		**42**				**42**
Sampaio da Silva et al. 2006		42				42
**Serrasalmus rhombeus (Linnaeus 1766)**		**40**	**22**	**2**	**8**	**72**
Castilhos et al. 2015			16	2		18
Lebel et al. 1997		11				11
Matos et al. 2021					8	8
Sampaio da Silva et al. 2006		29				29
Vasconcellos et al. 2021			6			6
**Serrasalmus sp.**	**23**	**23**	**15**		**24**	**85**
Bidone et al. 1997			6			6
Brabo et al. 1999			3			3
Castilhos et al. 1998	15	6				21
Farias 2007					11	11
Hacon et al. 1997					12	12
Lebel et al. 1997		13				13
Passos et al. 2008		-				-
Uryu et al. 2001	8	4	6		1	19
**Sternopygus macrurus (Bloch & Schneider 1801)**				**5**		**5**
Castilhos et al. 2015				5		5
**Symphysodon aequifasciatus Pellegrin 1904**		**2**				**2**
Sampaio da Silva et al. 2006		2				2
**Triportheus albus Cope 1872**		**12**				**12**
Sampaio da Silva et al. 2006		12				12
**Triportheus auritus (Valenciennes 1850)**		**5**				**5**
Lino et al. 2018		5				5
**Triportheus sp.**		**6**			**7**	**13**
Castilhos et al. 2012					7	7
Lebel et al. 1997		6				6
Passos et al. 2008		-				-
**Zungaro zungaro (Humboldt 1821)**	**40**	**17**			**29**	**86**
Akagi et al. 1995					4	4
Castilhos et al. 1998	28					28
dos Santos et al. 2000		10				10

Farias 2007						1	1
Hacon et al. 1997						18	18
Hacon et al. 2000						-	-
Lima et al. 2000	10						10
Martín-Doimeadios et al. 2014		5					5
Uryu et al. 2001	2	2				6	10
**Unspecified species**	**1657**	**3678**	**1430**		**70**	**445**	**7280**
Akagi et al. 1995		1				1	2
Castilhos et al. 2012						4	4
Dorea et al. 2005			184			181	365
Hacon et al. 1997						4	4
Hacon et al. 2003					70	254	324
Lacerda e Pfeiffer 1992		-					-
Lebel et al. 1997		8					8
Santos et al. 2003	1603	3603	1238				6444
Uryu et al. 2001	54	66	8			1	129
**Unspecified number of samples**		**1123**					**1123**
Passos et al. 2008		1123					1123
**Total**	**2509**	**8609**	**2031**	**30**	**70**	**864**	**14113**

Figure S5. Hg concentration (mg/kg) in fishes from Tapajós river basin, distributed in relation to trophic level (x-axis) and sub-basins(colors). Pointed lines delimit the threshold of Hg concentration permitted by Brazilian Government (ANVISA) for piscivorous (red) and non-piscivorous species (gray).

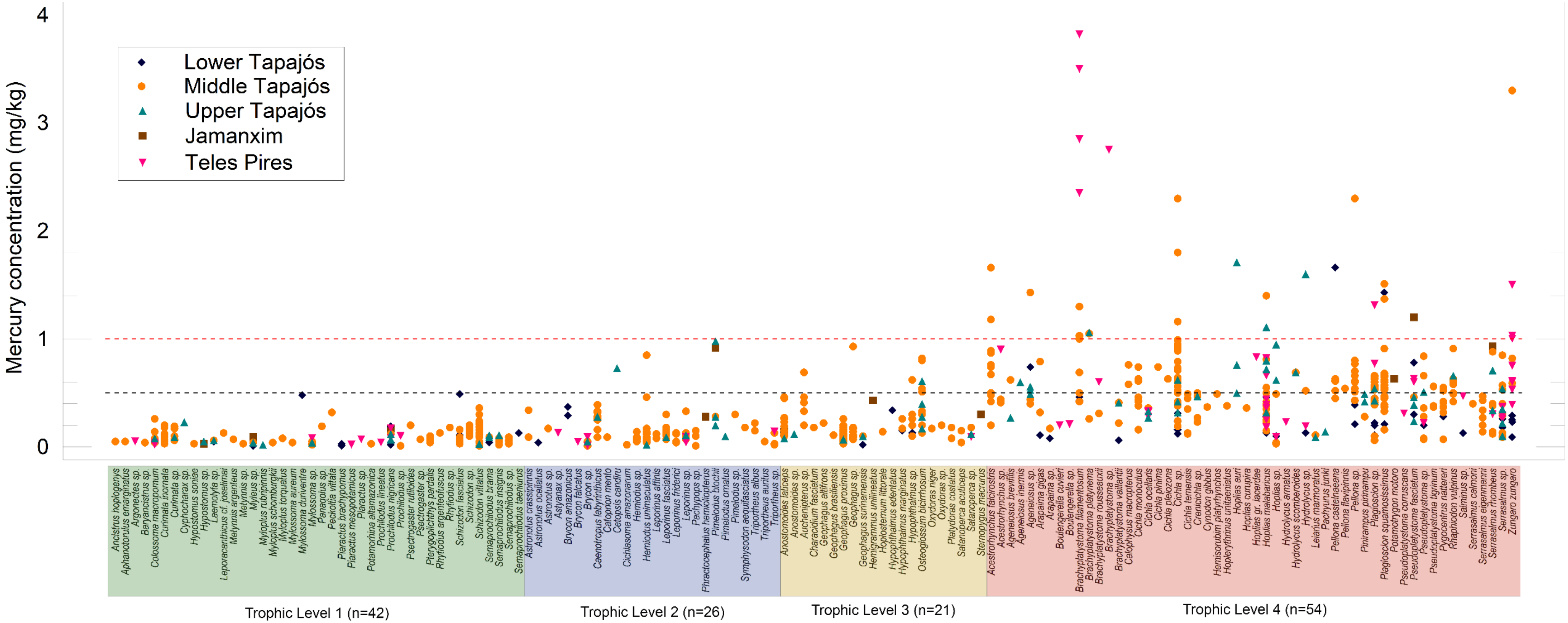

Figure S6. Spatial autocorrelation analysis of mercury (Hg) bioaccumulation in piscivorous fish across the Tapajós river basin, using the Moran's I index. A moderate correlation (I=0.360) indicates a clustering of high Hg concentration points, particularly in the middle Tapajós region. The figure was done with GeoDa (v.1.22.0.4).

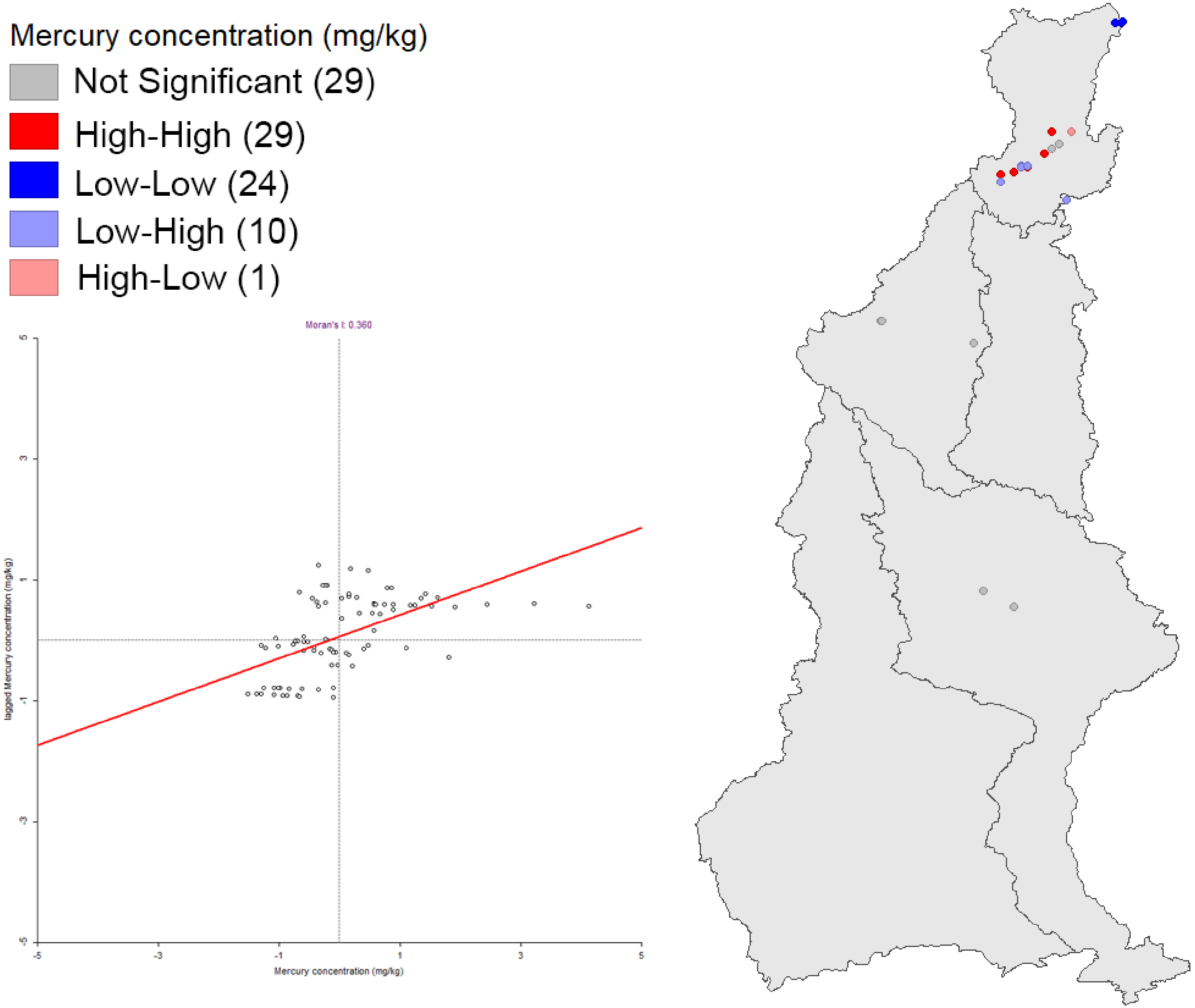

Table S7. Range values (maximum-minimum) of Hg concentration in fish from the Tapajós river basin, distributed by trophic level and sub-basin, with a statistical summary.

Sub-basin	Mercury concentration (mg/kg)											
	Trophic level 1 (min-max)	Median		Trophic level 2 (min-max)	Median		Trophic level 3 (min-max)	Median		Trophic level 4 (min-max)	Median		
Lower Tapajós	0.01 - 0.49	0.03	KW: 9.495, p=0.05	0.04 - 0.37	0.18	KW: 7.988, p=0.092	0.02 - 0.34	0.14	KW: 3.198, p=0.525	0.06 - 1.66	0.22	a	KW: 42,687, p=1.202 x 10-8
Middle Tapajós	0.01 - 0.36	0.07		0.01 - 0.85	0.12		0.03 - 0.93	0.18		0.03 - 3.30	0.49	b	
Upper Tapajós	0.02 - 0.23	0.07		0.02 - 0.98	0.16		0.07 - 0.61	0.12		0.09 - 1.71	0.47	abc	
Jamanxim	0.03 - 0.17	0.09		0.28 – 0.92	0.6		0.14 - 0.63	0.3		0.63 - 1.20	0.93	abc	
Teles Pires	0.02 - 0.17	0.05		0.01 - 0.14	0.08		0.09 - 0.09	0.09		0.09 - 3.82	0.58	c	

Table S8. Overview of Hg measurement counts and publication trends across decades, including statistical analysis of article numbers and their association with Hg measurements

	THg/Decade				Number of REF/Decade					Lineal Regresion	
**Dec**	**min-max**	**M**	**SD**		**min-max**	**M**	**SD**			**THg/Dec**	**THg/Ref**
**1**	0.008-3.82	0.26	0.40	KW: 0.13 p: 0.94	1-18	11.0	4,66	c	Kw: 457.81 p: 0	r^2: 0.09 β: 0.03 p: 0.000 cc:0.03	r^2: 0.22 β: -0.78 p: 0.000 cc: -0.05
**2**	0.01-3.5	0.2	0.40		1-8	4.0	1,62	a			
**3**	0.02-3.3	0.22	0.49		1-10	5.0	2,63	b			

Min-max: minimum and maximum values of THg concentration (mg/kg); M: median; SD: Standard deviation; β: Y intersection; cc: Correlation coefficient

Table S9. Statistical analysis of Hg concentration variations by trophic level.

	TL 1					TL 2				TL 3				TL 4			
**Dec**	**min-max**	**M**	**SD**			**min-max**	**M**	**SD**		**min-max**	**M**	**SD**		**min-max**	**M**	**SD**	
**1**	0.008-0.24	0.052	0.04	a	Kw: 15.13 p: 0.00	0.01-0.33	0.09	0.08	Kw: 4.14 p: 0.13	0.02-0.93	0.15	0.23	Kw: 1.22 p: 0.54	0.06-3.82	0.42	0.43	Kw: 10.18 p: 0.006
**2**	0.01-0.49	0.1	0.10	b		0.01-0.98	0.14	0.23		0.07-0.8	0.18	0.17		0.03-3.5	0.52	0.49	
**3**	0.02-0.32	0.08	0.07	ab		0.02-0.37	0.12	0.10		0.07-0.34	0.18	0.09		0.086-3.82	0.54	0.58	

Min-max: minimum and maximum values of THg concentration (mg/kg); M: median; SD: Standard deviation.

Table S10. Minimum and maximum Hazard Quotient (THQ) evaluation values of human health risk for Hg exposure by ingestion of contaminated fish from Tapajós river basin. The safe limit is THQ <1.

		Lower Tapajós	Middle Tapajós	Upper Tapajós	Jamanxim	Teles Pires
**Trophic Level**	Species	***Min-Max (Number of average Hg values)***	***Min-Max (Number of average Hg values)***	***Min-Max (Number of average Hg values)***	***Min-Max (Number of average Hg values)***	***Min-Max (Number of average Hg values)***
	Argonectes sp.	-	-	-	-	14.95 (1)
	Colossoma macropomum	**0.43-0.95 (3)**	0.66-4.32 (7)	1.4 (1)	-	**0.37-0.6 (2)**
	Curimata inornata	-	0.53-3.39 (13)	-	-	-
	Curimata sp.	-	1.0-3.16 (6)	1.49 (1)	-	-
	Cyphocharax sp.	-	-	3.82 (1)	-	-
	***Hypostomus* sp.**	-	-	**0.83 (1)**	**0.50 (1)**	-
	Laemolyta sp.	**0.86 (1)**	1.0 (1)	1.0 (1)	-	-
	Metynnis argenteus	-	1.11 (1)	-	-	-
	***Metynnis* sp.**	-	**0.5 (1)**	-	-	-
	***Myleus* sp.**	**0.2 (1)**	**0.61 (1)**	**0.61 (1)**	1.49 (1)	-
	Myloplus rubripinnis	-	-	**0.33 (1)**	-	-
	Myloplus schomburgkii	-	**0.66 (1)**	-	-	-
	Myloplus torquatus	-	1.33 (1)	-	-	-
**1**	***Mylossoma aureum***	-	**0.6 (1)**	-	-	-
	Mylossoma duriventre	7.99 (1)	-	-	-	-
	Mylossoma sp.	**0.49 (1)**	**0.33-0.85 (8)**	**0.7 (1)**	-	1.33 (1)
	Piaractus brachypomus	**0.14-0.5 (2)**	-	-	-	-
	Piaractus mesopotamicus	-	-	-	-	**0.25-0.33 (2)**
	Piaractus sp.	-	-	-	-	1.21 (1)
	Potamorhina altamazonica	-	**0.47-0.51 (3)**	-	-	-
	Prochilodus lineatus	-	-	-	-	**0.66 (1)**
	Prochilodus nigricans	0.27-3.16 (3)	2.01 (1)	1.16-1.99 (2)	2.82 (1)	1.33-2.82 (2)
	***Prochilodus* sp.**	-	**0.17 (1)**	-	-	1.63 (1)
	Psectrogaster rutiloides	-	3.32 (1)	-	-	-
	Psectrogaster sp.	-	1.16 (1)	-	-	-
	Pterygoplichthys pardalis	-	0.66 (1)	-	-	-

	Rhytiodus argenteofuscus	-	2.18 (1)	-	-	-
	Rhytiodus sp.	-	2.99 (1)	-	-	-
	Schizodon fasciatus	1.83-8.1 (2)	0.47-2.71 (6)	-	-	-
	Schizodon sp.	-	1.66-3.32 (6)	-	-	-
	Schizodon vittatus	-	0.18-5.9 (16)	**0.5 (1)**	-	-
	Semaprochilodus brama	**0.6 (1)**	1.44 (1)	1.44-1.86 (2)	-	-
	Semaprochilodus insignis	-	0.4-1.33 (3)	1.83 (1)	-	-
	Semaprochilodus sp.	**0.47 (1)**	0.5-1.49 (5)	-	-	-
	Semaprochilodus taeniurus	2.16 (1)	-	-	-	-
	Astronotus crassipinnis	-	1.49-5.65 (2)	-	-	-
	Astronotus ocellatus	**0.6 (1)**	-	-	-	-
	Astronotus sp.	-	2.82 (1)	-	-	-
	Brycon amazonicus	4.83-6.14 (2)	-	-	-	-
	Brycon falcatus	-	-	-	-	**0.86 (1)**
	Brycon sp.	-	**0.17-0.86 (2)**	**0.86 (1)**	-	0.17-1.43 (2)
	Catoprion mento	-	1.44 (1)	-	-	-
	Cichlasoma amazonarum	-	**0.25 (1)**	-	-	-
	Hemiodus sp.	-	0.83-2.33 (7)	-	-	-
	Hemiodus unimaculatus	-	0.58-14.13 (18)	**0.33 (1)**	-	-
	Leporinus affinis	-	1.33-1.94 (2)	-	-	-
**2**	*Leporinus fasciatus*	-	1.13-4.9 (11)	1.49 (1)	-	-
	Leporinus friderici	-	0.73-2.18 (4)	-	-	-
	Leporinus sp.	1.14 (1)	1.09-5.48 (8)	1.79 (1)	-	0.58-1.45 (2)
	Pachypops sp.	-	0.17-2.49 (3)	-	-	-
	Phractocephalus hemioliopterus	-	-	-	4.65 (1)	-
	Pimelodus blochii	-	4.65 (1)	3.32-16.28 (3)	15.28 (1)	-
	Pimelodus ornatus	-	-	1.58 (1)	-	-
	Pimelodus sp.	-	4.98 (1)	-	-	-
	Triportheus albus	-	2.54-3.62 (2)	-	-	-
	Triportheus auritus	-	**0.83 (1)**	-	-	-
	Triportheus sp.	-	0.33-2.16 (3)	-	-	2.26-2.26 (1)

3	*Anostomoides laticeps*	-	1.58-7.64 (11)	1.33 (1)	2.33 (1)	-
	Anostomoides sp.	-	-	2.04 (1)	-	-
	Auchenipterus sp.	-	3.32-11.46 (6)	-	-	-
	Geophagus altifrons	-	3.65 (1)	-	-	-
	Geophagus brasiliensis	-	1.83 (1)	-	-	-
	Geophagus proximus	-	0.55-4.25 (18)	1.16 (1)	-	-
	Geophagus sp.	-	1.16-15.44 (9)	-	-	-
	Geophagus surinamensis	**0.32 (1)**	1.66 (1)	1.66 (1)	-	-
	Hoplosternum littorale	-	2.39 (1)	-	-	-
	Hypophthalmus edentatus	5.65 (1)	-	-	-	-
	Hypophthalmus marginatus	2.47 (1)	2.84-4.32 (2)	-	-	-
	Hypophthalmus sp.	2.11 (1)	1.66-10.23 (6)	-	-	-
	Osteoglossum bicirrhosum	-	2.09-13.6 (8)	2.89-10.1 (4)	-	-
	Oxydoras niger	-	2.77 (1)	-	-	-
	Oxydoras sp.	-	3.32 (1)	-	-	-
	Platydoras costatus	-	1.25-2.99 (2)	-	-	-
	Satanoperca acuticeps	-	0.63-2.49 (2)	-	-	-
	Satanoperca sp.	-	2.99 (1)	1.99 (1)	-	1.46 (1)
4	*Acestrorhynchus falcirostris*	-	3.29-27.55 (12)	-	-	-
	Acestrorhynchus sp.	-	6.74-7.22 (2)	-	-	14.95 (1)
	Ageneiosus brevifilis	-	10.21 (1)	4.42 (1)	-	-
	Ageneiosus inermis	-	-	9.98 (1)	-	-
	Ageneiosus sp.	12.22 (1)	6.64-23.76 (3)	8.19-9.3 (3)	-	-
	Arapaima gigas	1.88 (1)	5.36-13.12 (2)	-	-	-
	Arapaima sp.	1.33 (1)	2.82 (1)	-	-	-
	Boulengerella cuvieri	-	-	-	-	3.3 (1)
	Boulengerella sp.	-	-	-	-	3.4 (1)
	Brachyplatystoma filamentosum	7.19-7.56 (2)	7.01-21.59 (6)	-	-	38.99-63.44 (4)
	Brachyplatystoma platynema	-	4.32-17.39 (2)	17.54 (1)	-	-
	Brachyplatystoma rousseauxii	-	5.15 (1)	-	-	9.96 (1)
	Brachyplatystoma sp.	-	-	-	-	45.67 (1)

Brachyplatystoma vaillantii	1.01 (1)	3.65-6.81 (2)	6.81 (1)	-	-
Calophysus macropterus	-	9.63-12.62 (2)	-	-	-
Cichla monoculus	-	2.71-12.29 (8)	-	-	-
Cichla ocellaris	-	5.98 (1)	4.43-5.48 (2)	-	4.65-5.4 (3)
Cichla pinima	-	12.22 (1)	-	-	-
Cichla pleiozona	-	10.46 (1)	-	-	-
Cichla sp.	1.92-5.08 (9)	3.16-38.2 (28)	5.26-10.3 (3)	-	-
Cichla temensis	-	1.91-8.14 (5)	-	-	-
Crenicichla sp.	-	3.74-8.3 (4)	7.81 (1)	-	-
Cynodon gibbus	-	6.14 (1)	-	-	-
Hemisorubim platyrhynchos	-	8.14 (1)	-	-	-
Hoplerythrinus unitaeniatus	-	6.31 (1)	-	-	-
Hoplias auri	-	-	8.22-28.4 (3)	-	-
Hoplias curupira	-	5.98 (1)	-	-	-
Hoplias gr. *lacerdae*	-	-	-	-	13.7 (1)
Hoplias malabaricus	2.21-8.97 (4)	2.52-23.22 (13)	5.35-18.35 (4)	-	2.94-13.53 (7)
Hoplias sp.	1.69 (1)	0.5-8.14 (3)	10.3-15.78 (2)	-	1.43 (1)
Hydrolycus armatus	-	-	-	-	3.8 (1)
Hydrolycus scomberoides	-	11.46 (1)	11.46 (1)	-	-
Hydrolycus sp.	2.09 (1)	8.64 (1)	26.57 (1)	-	3.21 (1)
Leiarius marmoratus	-	1.83 (1)	1.54 (1)	-	-
Pachyurus junki	-	-	2.33 (1)	-	-
Pellona castelnaeana	27.53 (1)	7.47-10.51 (4)	-	-	-
Pellona flavipinnis	-	8.97 (1)	-	-	-
Pellona sp.	3.52-7.01 (4)	7.14-38.2 (13)	-	-	-
Pinirampus pirinampu	-	4.65 (1)	6.96-8.14 (2)	-	-
Plagioscion sp.	1.83-3.72 (3)	1.0-10.98 (16)	7.14-9.0 (2)	-	12.79-21.76 (2)
Plagioscion squamosissimus	3.56-23.8 (2)	2.72-25.08 (24)	-	-	-
Potamotrygon motoro	-	-	-	10.46 (1)	-
Pseudoplatystoma corruscans	-	-	-	-	5.15 (1)
Pseudoplatystoma fasciatum	5.02-12.95 (2)	7.64 (1)	3.99-7.64 (3)	19.93 (1)	9.96-10.46 (3)

Pseudoplatystoma sp.	3.3 (1)	1.16-13.93 (6)	8.47 (1)	-	3.65 (1)
Pseudoplatystoma tigrinum	-	6.16-9.3 (3)	-	-	-
Pygocentrus nattereri	4.66 (1)	1.23-9.13 (7)	-	-	-
Rhaphiodon vulpinus	-	6.98-15.11 (8)	10.96 (1)	-	-
Salminus sp.	2.16 (1)	-	-	-	7.81-7.81 (1)
Serrasalmus calmoni	-	6.64 (1)	-	-	-
Serrasalmus eigenmanni	-	2.23-7.82 (6)	-	-	-
Serrasalmus rhombeus	-	2.03-14.63 (5)	5.65-11.79 (2)	15.44 (1)	5.05 (1)
Serrasalmus sp.	2.94-4.3 (2)	1.49-14.08 (12)	1.66-8.98 (4)	-	4.3-6.14 (3)
Zungaro zungaro	1.53-4.87 (4)	9.72-54.8 (3)	-	-	6.48-24.91 (9)

Table S11. Safe daily consumption quantity in grams (g) of 129 fish species of interest for human consumption in the Tapajós River basin, within each subunit of the basin, for each trophic level. Values above the reference value (116.25 g) are highlighted in bold.

**Trophic Level**	Species	Lower Tapajós	Middle Tapajós	Upper Tapajós	Jamanxim	Teles Pires
	***Argonectes* sp.**	-	-	-	-	**149**
	Colossoma macropomum	**189**	96	83	-	**256**
	Curimata inornata	-	112	-	-	-
	Curimata sp.	-	80	78	-	-
	Cyphocharax sp.	-	-	30	-	-
	***Hypostomus* sp.**	-	-	**140**	**233**	-
	***Laemolyta* sp.**	**135**	**117**	**117**	-	-
	Metynnis argenteus	-	104	-	-	-
**1**	***Metynnis* sp.**	-	**233**	-	-	-
	***Myleus* sp.**	**583**	**189**	**189**	78	-
	Myloplus rubripinnis	-	-	**350**	-	-
	Myloplus schomburgkii	-	**175**	-	-	-
	Myloplus torquatus	-	88	-	-	-
	Mylossoma aureum	-	**194**	-	-	-
	Mylossoma duriventre	15	-	-	-	-
	***Mylossoma* sp.**	**236**	**258**	**167**	-	88
	Piaractus brachypomus	**519**	-	-	-	-

	Piaractus mesopotamicus	-	-	-	-	**409**
	Piaractus sp.	-	-	-	-	96
	Potamorhina altamazonica	-	**242**	-	-	-
	Prochilodus lineatus	-	-	-	-	**175**
	Prochilodus nigricans	**223**	58	79	41	65
	***Prochilodus* sp.**	-	**700**	-	-	71
	Psectrogaster rutiloides	-	35	-	-	-
	Psectrogaster sp.	-	100	-	-	-
	Pterygoplichthys pardalis	-	101	-	-	-
	Rhytiodus argenteofuscus	-	53	-	-	-
	Rhytiodus sp.	-	39	-	-	-
	Schizodon fasciatus	39	116	-	-	-
	Schizodon sp.	-	53	-	-	-
	Schizodon vittatus	-	98	**233**	-	-
	Semaprochilodus brama	**194**	80	72	-	-
	Semaprochilodus insignis	-	**173**	64	-	-
	***Semaprochilodus* sp.**	**250**	**136**	-	-	-
	Semaprochilodus taeniurus	54	-	-	-	-
**2**	*Astronotus crassipinnis*	-	50	-	-	-
	Astronotus ocellatus	**194**	-	-	-	-
	***Astronotus* sp.**	-	**124**	-	-	-
	Brycon amazonicus	22	-	-	-	-
	Brycon falcatus	-	-	-	-	**135**
	***Brycon* sp.**	-	**418**	**135**	-	**391**
	Catoprion mento	-	80	-	-	-
	Cichlasoma amazonarum	-	**467**	-	-	-
	Hemiodus sp.	-	96	-	-	-
	Hemiodus unimaculatus	-	95	**350**	-	-
	Leporinus affinis	-	74	-	-	-
	Leporinus fasciatus	-	52	78	-	-
	Leporinus friderici	-	101	-	-	-
	***Leporinus* sp.**	102	65	65	-	**140**
	***Pachypops* sp.**	-	**272**	-	-	-
	Phractocephalus hemioliopterus	-	-	-	25	-

	Pimelodus blochii	-	25	22	8	-
	Pimelodus ornatus	-	-	74	-	-
	Pimelodus sp.	-	23	-	-	-
	Triportheus albus	-	39	-	-	-
	Triportheus auritus	-	**140**	-	-	-
	***Triportheus* sp.**	-	**212**	-	-	52
**3**	*Anostomoides laticeps*	-	38	88	50	-
	Anostomoides sp.	-	-	57	-	-
	Auchenipterus sp.	-	18	-	-	-
	Geophagus altifrons	-	32	-	-	-
	Geophagus brasiliensis	-	64	-	-	-
	Geophagus proximus	-	77	100	-	-
	Geophagus sp.	-	58	-	-	-
	Geophagus surinamensis	**368**	70	70	-	-
	Hoplosternum littorale	-	49	-	-	-
	Hypophthalmus edentatus	21	-	-	-	-
	Hypophthalmus marginatus	47	34	-	-	-
	Hypophthalmus sp.	55	32	-	-	-
	Osteoglossum bicirrhosum	-	23	24	-	-
	Oxydoras niger	-	42	-	-	-
	Oxydoras sp.	-	35	-	-	-
	Platydoras costatus	-	66	-	-	-
	Satanoperca acuticeps	-	116	-	-	-
	Satanoperca sp.	-	39	58	-	80
**4**	*Acestrorhynchus falcirostris*	-	12	-	-	-
	Acestrorhynchus sp.	-	17	-	-	8
	Ageneiosus brevifilis	-	11	26	-	-
	Ageneiosus inermis	-	-	12	-	-
	Ageneiosus sp.	10	13	13	-	-
	Arapaima gigas	62	16	-	-	-
	Arapaima sp.	88	41	-	-	-
	Boulengerella cuvieri	-	-	-	-	35
	Boulengerella sp.	-	-	-	-	34
	Brachyplatystoma filamentosum	16	10	-	-	2

Brachyplatystoma platynema	-	17	7	-	-
Brachyplatystoma rousseauxii	-	23	-	-	12
Brachyplatystoma sp.	-	-	-	-	8
Brachyplatystoma vaillantii	115	25	17	-	-
Calophysus macropterus	-	11	-	-	-
Cichla monoculus	-	18	-	-	-
Cichla ocellaris	-	19	24	-	23
Cichla pinima	-	10	-	-	-
Cichla pleiozona	-	11	-	-	-
Cichla sp.	43	13	17	-	-
Cichla temensis	-	33	-	-	-
Crenicichla sp.	-	22	15	-	-
Cynodon gibbus	-	19	-	-	-
Hemisorubim platyrhynchos	-	14	-	-	-
Hoplerythrinus unitaeniatus	-	18	-	-	-
Hoplias auri	-	-	9	-	-
Hoplias curupira	-	19	-	-	-
Hoplias gr. *lacerdae*	-	-	-	-	8
Hoplias malabaricus	27	21	12	-	20
***Hoplias* sp.**	69	**141**	9	-	81
Hydrolycus armatus	-	-	-	-	31
Hydrolycus scomberoides	-	10	10	-	-
Hydrolycus sp.	56	13	4	-	36
Leiarius marmoratus	-	64	75	-	-
Pachyurus junki	-	-	50	-	-
Pellona castelnaeana	4	13	-	-	-
Pellona flavipinnis	-	13	-	-	-
Pellona sp.	22	11	-	-	-
Pinirampus pirinampu	-	25	16	-	-
Plagioscion sp.	43	24	15	-	7
Plagioscion squamosissimus	19	14	-	-	-
Potamotrygon motoro	-	-	-	11	-
Pseudoplatystoma corruscans	-	-	-	-	23
Pseudoplatystoma fasciatum	16	15	21	6	12

Pseudoplatystoma sp.	35	42	14	-	32
Pseudoplatystoma tigrinum	-	17	-	-	-
Pygocentrus nattereri	25	28	-	-	-
Rhaphiodon vulpinus	-	12	11	-	-
Salminus sp.	54	-	-	-	15
Serrasalmus calmoni	-	18	-	-	-
Serrasalmus eigenmanni	-	27	-	-	-
Serrasalmus rhombeus	-	30	16	8	23
Serrasalmus sp.	34	30	34	-	23
Zungaro zungaro	40	8	-	-	10
